# Supplementary material for: Glycosylation of recombinant adeno-associated virus serotype 6
Source: Mol Ther Methods Clin Dev. 2024 Apr 25;32(2):101256. doi: 10.1016/j.omtm.2024.101256 (PMC11107246; doi:10.1016/j.omtm.2024.101256)
Supplement: Document S1. Figures S1–S13 and Tables S1 and S2 [file mmc1.pdf]

OMTM, Volume *32*

Supplemental information

Glycosylation of recombinant adeno-associated virus serotype 6

Yuki Yamaguchi, Kentaro Ishii, Sachiko Koizumi, Hiroaki Sakaue, Takahiro Maruno, Mitsuko Fukuhara, Risa Shibuya, Yasuo Tsunaka, Aoba Matsushita, Karin Bandoh, Tetsuo Torisu, Chie Murata-Kishimoto, Azusa Tomioka, Saho Mizukado, Hiroyuki Kaji, Yuji Kashiwakura, Tsukasa Ohmori, Atsushi Kuno, and Susumu Uchiyama

Table S1. Sample information of the lectin microarrays used for the PCA analysis shown in Figure 1B. The number and color correspond to the dot in PCA score plot.

The dot number	Sample	Amount (vg)
1	Sample 1	2.5×10^{9}
2	Sample 1	1×10^{10}
3	Sample 1	4×10^{10}
4	Sample 2	2.5×10^{9}
5	Sample 2	1×10^{10}
6	Sample 2	4×10^{10}
7	Sample 3	2.5×10^{9}
8	Sample 3	1×10^{10}
9	Sample 3	4×10^{10}
10	Sample 3	2.5×10^{9}
11	Sample 4	2.5×10^{9}
12	Sample 4	1×10^{10}
13	Sample 4	4×10^{10}
14	Sample 5	2.5×10^{9}
15	Sample 5	1×10^{10}
16	Sample 5	4×10^{10}
17	Sample 6	2.5×10^{9}
18	Sample 6	5×10^{9}

Table S2. The list of identified proteins contaminating Sample 4.

Several bovine serum proteins (colored blue) and human galectin 3-binding protein (colored red) were identified in Sample 4.

Accession	-10lgP	Coverage (%)	Area	#Peptides	Avg. Mass	Description
VP1_AAV6	215.39	74	3.81E+08	47	81411	VP1_AAV6
#CONTAM#Q7SIH1|A2MG_BOVIN	241.3	66	3.35E+08	77	167575	Alpha-2-macroglobulin OS=Bos taurus OX=9913 GN=A2M PE=1 SV=2
#CONTAM#A7E3W2|LG3BP_BOVIN	174.09	40	6.08E+07	18	62127	Galectin-3-binding protein OS=Bos taurus OX=9913 GN=LGALS3BP PE=1 SV=1
#CONTAM#Q3Y5Z3|ADIPO_BOVIN	115.09	32	2.42E+07	6	26133	Adiponectin OS=Bos taurus OX=9913 GN=ADIPOQ PE=1 SV=1
#CONTAM#P06748|NPM_HUMAN	147.68	46	2.23E+07	10	32575	Nucleophosmin OS=Homo sapiens OX=9606 GN=NPM1 PE=1 SV=2
#CONTAM#O46415|FRIL_BOVIN	129.49	63	2.01E+07	10	19988	Ferritin light chain OS=Bos taurus OX=9913 GN=FTL PE=2 SV=3
#CONTAM#P02792|FRIL_HUMAN	122.99	48	1.52E+07	9	20020	Ferritin light chain OS=Homo sapiens OX=9606 GN=FTL PE=1 SV=2
#CONTAM#P55072|TERA_HUMAN	199	68	8.51E+06	47	89322	Transitional endoplasmic reticulum ATPase OS=Homo sapiens OX=9606 GN=VCP PE=1 SV=4
#CONTAM#Q01853|TERA_MOUSE	199	68	8.51E+06	47	89322	Transitional endoplasmic reticulum ATPase OS=Mus musculus OX=10090 GN=Vcp PE=1 SV=4
#CONTAM#P46462|TERA_RAT	199	68	8.51E+06	47	89349	Transitional endoplasmic reticulum ATPase OS=Rattus norvegicus OX=10116 GN=Vcp PE=1 SV=3
#CONTAM#P00761|TRYP_PIG	93.86	34	6.47E+06	5	24409	Trypsin OS=Sus scrofa OX=9823 PE=1 SV=1
#CONTAM#P04406|G3P_HUMAN	153.88	58	5.76E+06	17	36053	Glyceraldehyde-3-phosphate dehydrogenase OS=Homo sapiens OX=9606 GN=GAPDH PE=1 SV=3

#CONTAM#Q5R8J7|FRIH_PONAB	138.85	72	5.65E+06	13	21226	Ferritin heavy chain OS=Pongo abelii OX=9601 GN=FTH1 PE=2 SV=3
#CONTAM#P02794|FRIH_HUMAN	138.85	72	5.65E+06	13	21226	Ferritin heavy chain OS=Homo sapiens OX=9606 GN=FTH1 PE=1 SV=2
#CONTAM#P14618|KPYM_HUMAN	168.02	53	5.50E+06	22	57937	Pyruvate kinase PKM OS=Homo sapiens OX=9606 GN=PKM PE=1 SV=4
#CONTAM#Q01105|SET_HUMAN	117.82	31	5.11E+06	9	33489	Protein SET OS=Homo sapiens OX=9606 GN=SET PE=1 SV=3
#CONTAM#Q9BXJ4|C1QT3_HUMAN	100.58	21	4.95E+06	5	26994	Complement C1q tumor necrosis factor-related protein 3 OS=Homo sapiens OX=9606 GN=C1QTNF3 PE=1 SV=1
#CONTAM#Q9BQA1|MEP50_HUMAN	145.04	43	4.67E+06	11	36724	Methylosome protein 50 OS=Homo sapiens OX=9606 GN=WDR77 PE=1 SV=1
#CONTAM#Q07021|C1QBP_HUMAN	118.24	41	4.42E+06	7	31362	Complement component 1 Q subcomponent-binding protein mitochondrial OS=Homo sapiens OX=9606 GN=C1QBP PE=1 SV=1
#CONTAM#P04075|ALDOA_HUMAN	163.86	73	4.41E+06	22	39420	Fructose-bisphosphate aldolase A OS=Homo sapiens OX=9606 GN=ALDOA PE=1 SV=2
#CONTAM#P02769|ALBU_BOVIN	178.52	58	3.64E+06	32	69294	Albumin OS=Bos taurus OX=9913 GN=ALB PE=1 SV=4
#CONTAM#P15636|API_ACHLY	132.47	16	3.33E+06	9	68125	Protease 1 OS=Achromobacter lyticus OX=224 PE=1 SV=1
#CONTAM#Q08380|LG3BP_HUMAN	139.96	29	2.95E+06	14	65331	Galectin-3-binding protein OS=Homo sapiens OX=9606 GN=LGALS3BP PE=1 SV=1
#CONTAM#P30048|PRDX3_HUMAN	108.06	31	2.82E+06	7	27693	Thioredoxin-dependent peroxide reductase mitochondrial OS=Homo sapiens OX=9606 GN=PRDX3 PE=1 SV=3
#CONTAM#Q5REY3|PRDX3_PONAB	108.06	31	2.82E+06	7	27700	Thioredoxin-dependent peroxide reductase mitochondrial OS=Pongo abelii OX=9601 GN=PRDX3 PE=2 SV=1

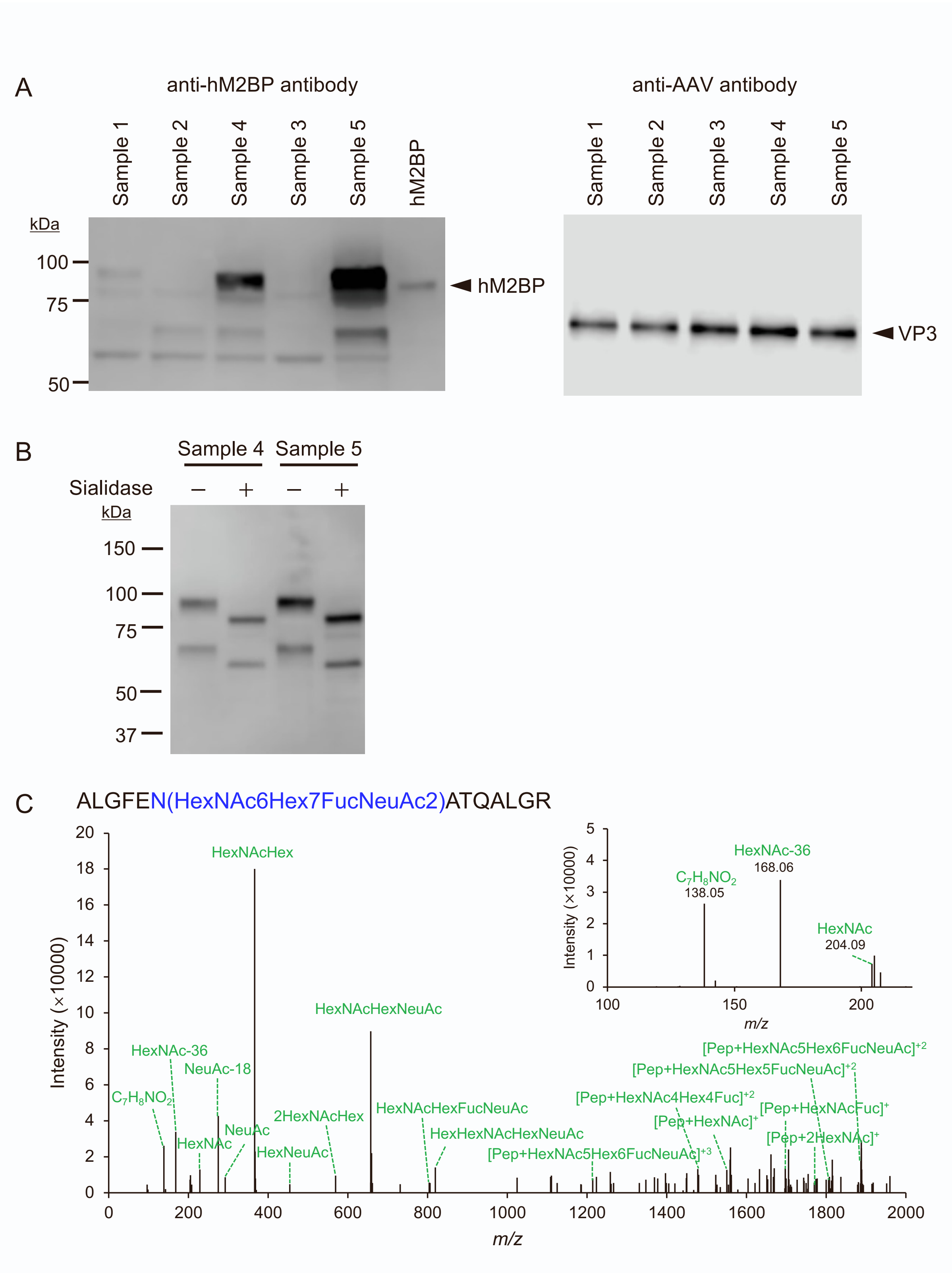

Figure S1. (A) Western blots of Samples 1–5 detected with anti-hM2BP antibody (left) and anti-AAV viral proteins antibody (right). (B) Western blot analysis of Samples 4 and 5 detected with anti-hM2BP antibody with and without sialidase treatment. (C) The CID mass spectra of hM2BP A64–R76 modified with HexNAc6Hex7FucNeuAc2 in Sample 4.

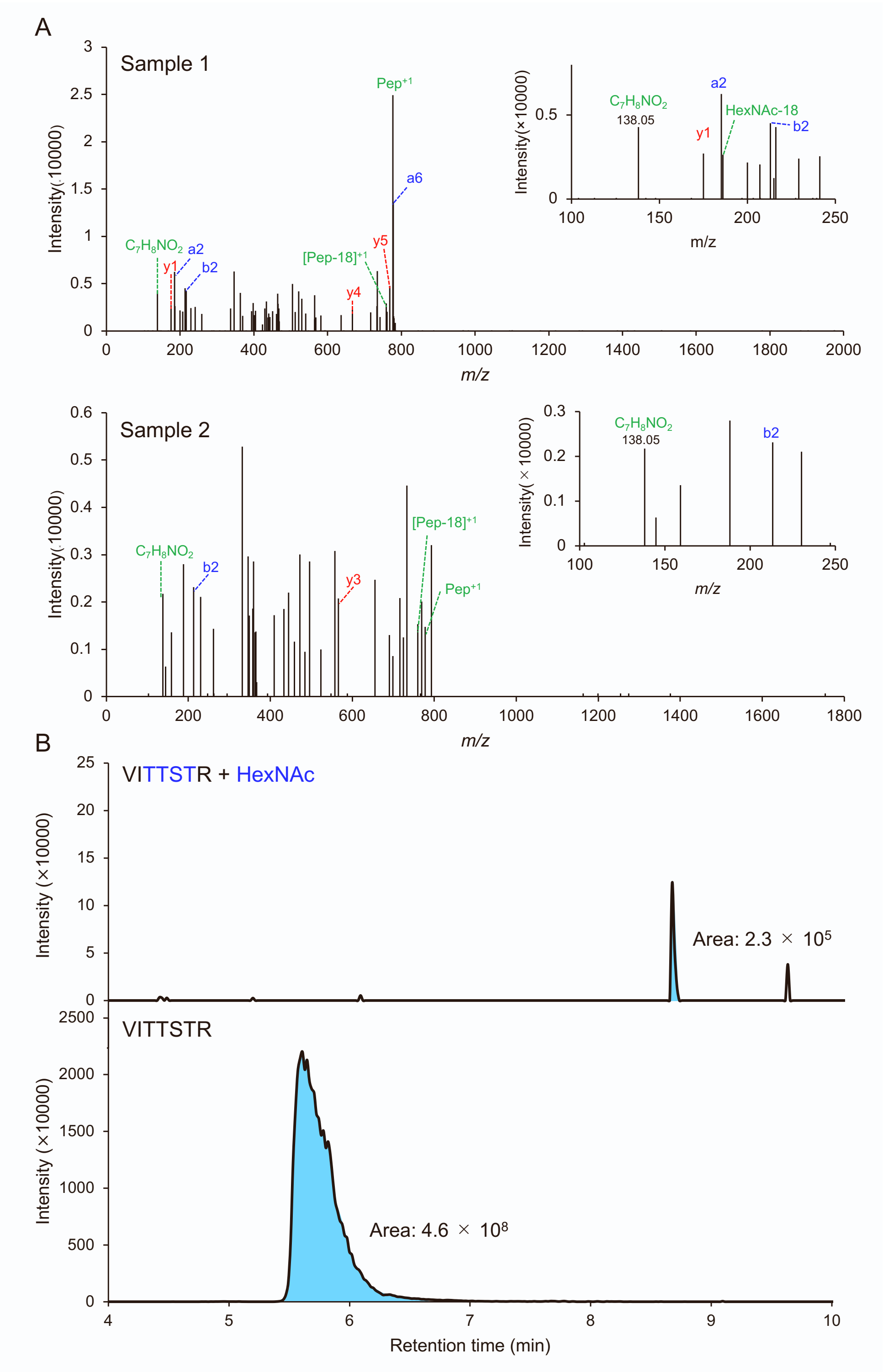

Figure S2. (A) The CID mass spectra of V239–R245 modified with HexNAc for Samples 1 and 2. (B) Extracted ion chromatograms (XICs) of V239–R245 modified with HexNAc (*m/z* 389.2284) (top) and unmodified V239–R245 (*m/z* 490.7697) (bottom). Each MS area was calculated using Byos software. The amount of peptide modified with *O*-GlcNAc (a) was calculated with the equation: a = [2.3×10^5 of *O*-GlcNAc modified peptide's MS area] / ([*O*-GlcNAc modified peptide's MS area] + [4.6×10^8 of unmodified peptide's MS area]). The percentage of particle modified with *O*-GlcNAc (p) was calculated with the equation: $p = 1^{60} - (1\text{-}0.0005)^{60}$.

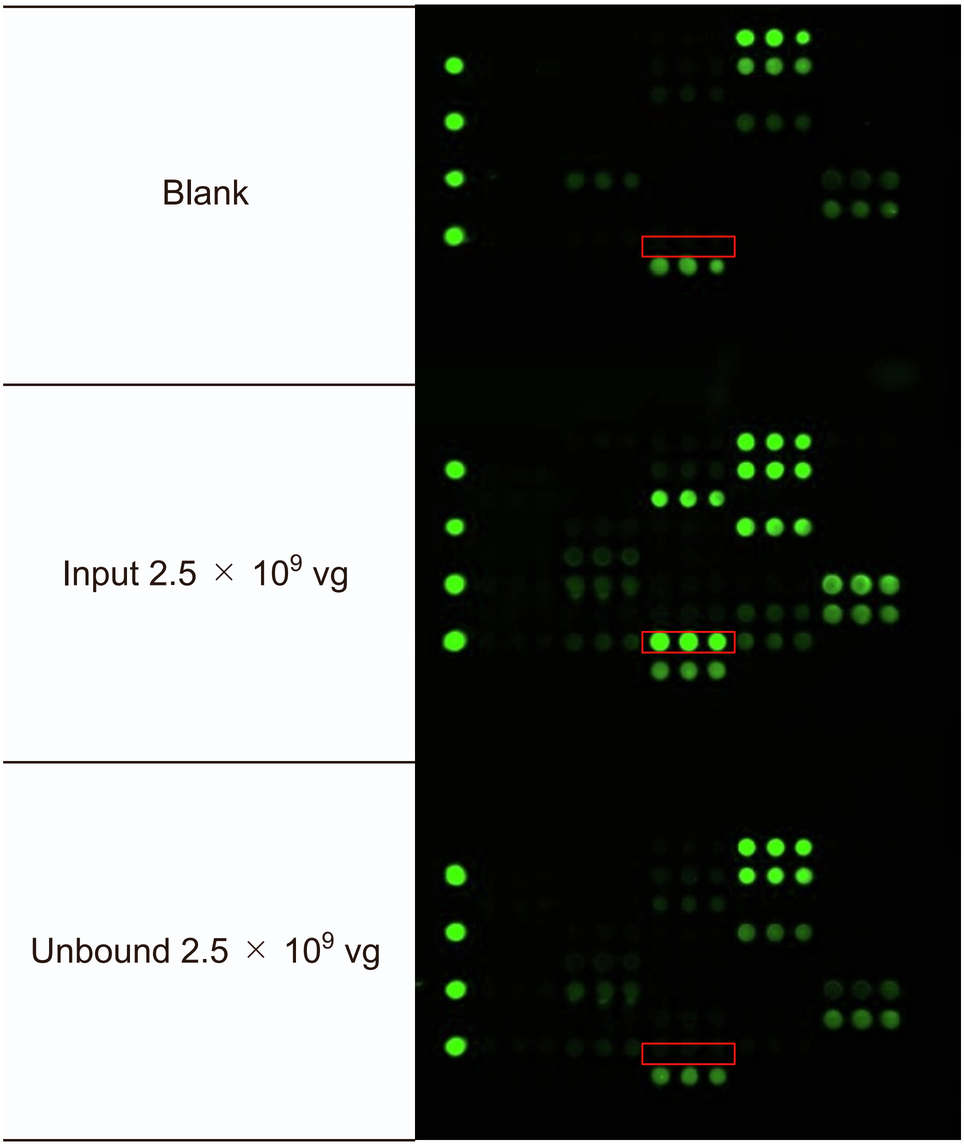

Figure S3. The signals detected by lectin microarray for 2.5 × 10^9 vg rAAV6 samples as described for western blotting. The area of ABA signals is marked by in a red box. The Blank array shows the high signals of *Lycopersicon esculentum* lectin (LEL), *Solanum tuberosum* lectin (STL), and *Urtica dioica* lectin (UDA) in triplicate spots caused by binding to the detection antibody. The signals of those lectins were therefore excluded from the lectin microarray analysis.

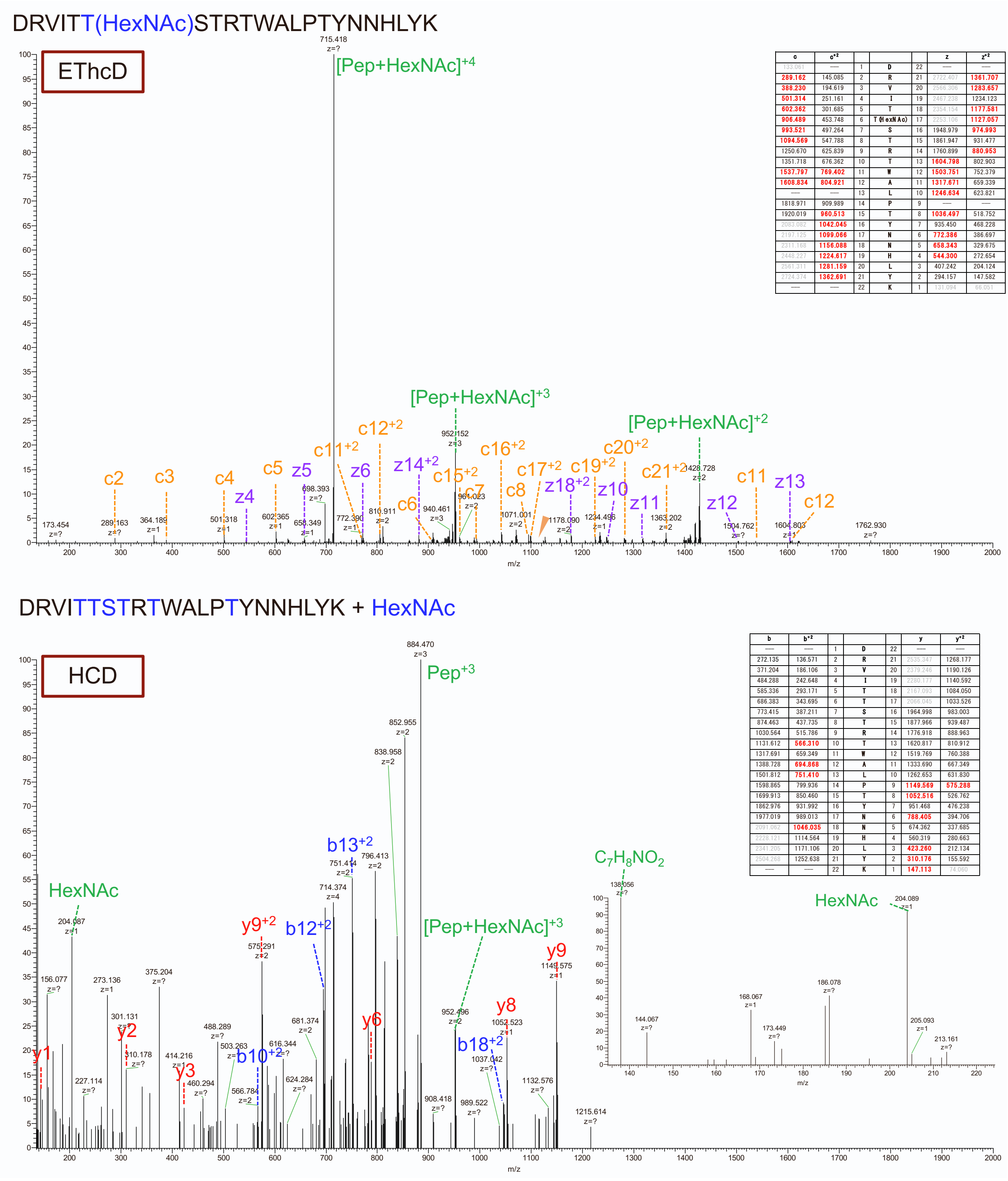

c	c+2				z	z+2
133.061	---	1	D	22	---	---
289.162	145.085	2	R	21	2722.407	1361.707
388.230	194.619	3	V	20	2566.306	1283.657
501.314	251.161	4	I	19	2467.238	1234.123
602.362	301.685	5	T	18	2354.154	1177.581
906.489	453.748	6	T(HexNAc)	17	2253.106	1127.057
993.521	497.264	7	S	16	1948.979	974.993
1094.569	547.788	8	T	15	1861.947	931.477
1250.670	625.839	9	R	14	1760.899	880.953
1351.718	676.362	10	T	13	1604.798	802.903
1537.797	769.402	11	W	12	1503.751	752.379
1608.834	804.921	12	A	11	1317.671	659.339
---	---	13	L	10	1246.634	623.821
1818.971	909.989	14	P	9	---	---
1920.019	960.513	15	T	8	1036.497	518.752
2083.082	1042.045	16	Y	7	935.450	468.228
2197.125	1099.066	17	N	6	772.386	386.697
2311.168	1156.088	18	N	5	658.343	329.675
2448.227	1224.617	19	H	4	544.300	272.654
2561.311	1281.159	20	L	3	407.242	204.124
2724.374	1362.691	21	Y	2	294.157	147.582
---	---	22	K	1	131.094	66.051

b	b+2				y	y+2
---	---	1	D	22	---	---
272.135	136.571	2	R	21	2535.347	1268.177
371.204	186.106	3	V	20	2379.246	1190.126
484.288	242.648	4	I	19	2280.177	1140.592
585.336	293.171	5	T	18	2167.093	1084.050
686.383	343.695	6	T	17	2066.045	1033.526
773.415	387.211	7	S	16	1964.998	983.003
874.463	437.735	8	T	15	1877.966	939.487
1030.564	515.786	9	R	14	1776.918	888.963
1131.612	566.310	10	T	13	1620.817	810.912
1317.691	659.349	11	W	12	1519.769	760.388
1388.728	694.868	12	A	11	1333.690	667.349
1501.812	751.410	13	L	10	1262.653	631.830
1598.865	799.936	14	P	9	1149.569	575.288
1699.913	850.460	15	T	8	1052.516	526.762
1862.976	931.992	16	Y	7	951.468	476.238
1977.019	989.013	17	N	6	788.405	394.706
2091.062	1046.035	18	N	5	674.362	337.685
2228.121	1114.564	19	H	4	560.319	280.663
2341.205	1171.106	20	L	3	423.260	212.134
2504.268	1252.638	21	Y	2	310.176	155.592
---	---	22	K	1	147.113	74.060

Figure S4. The EThcD and HCD mass spectra of D237–K258 with HexNAc identified in the unbound fraction of rAAV6.

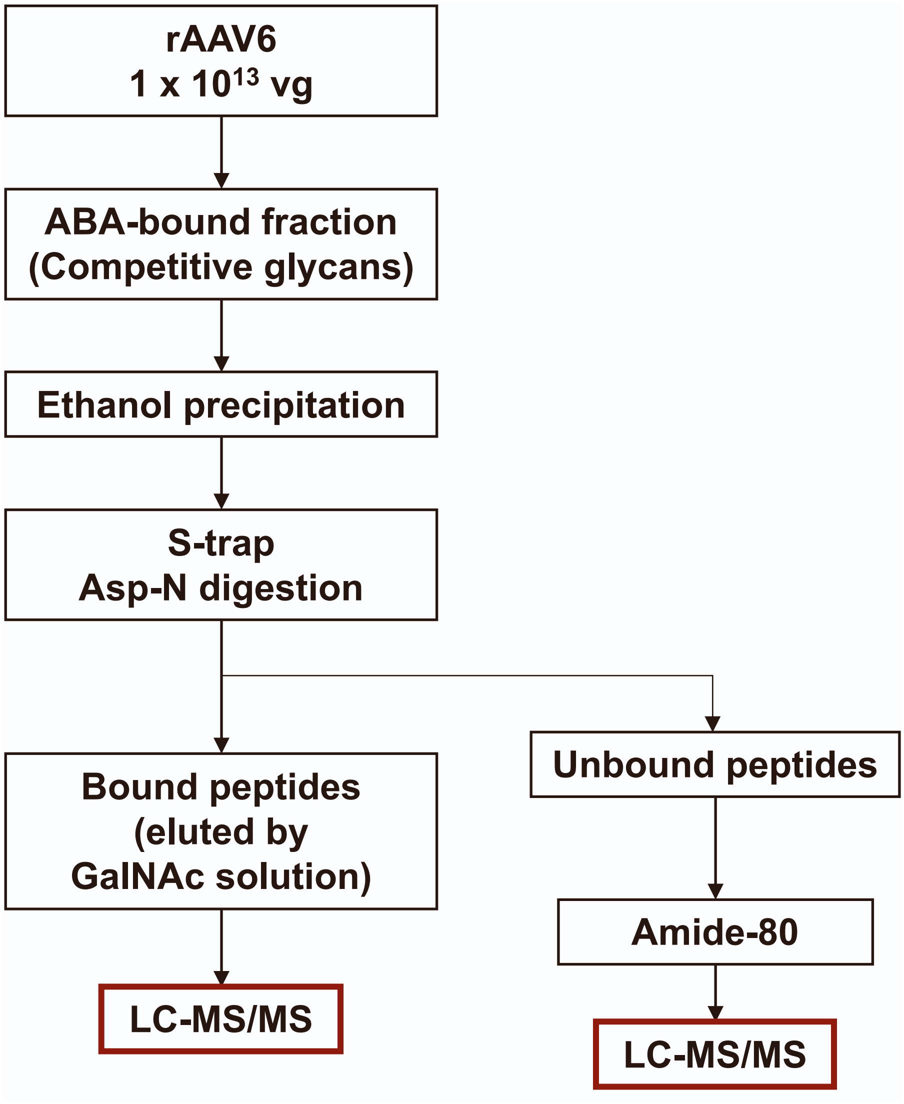

Figure S5. The experimental workflow of LC-MS/MS analysis for the bound fraction of rAAV6 using Sample 1.

A

O-glycan							
DSSSGIGKTGQQPAKKRLNFGQTG (154-177)							
cluster_no:1/member:3							
peak_no	m/z	rt	intensity	Hex	HexNAc	dHex	NeuAc
1	2462.268	40.99	158184080	0	0	0	0
570	2665.354	40.79	184328	0	1	0	0
217	2665.354	39.99	639783				
878	2868.434	39.59	98248	0	2	0	0

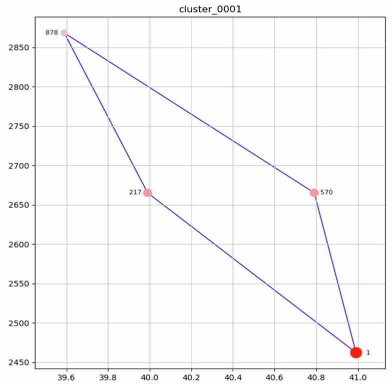

O-glycan							
DPQPLGEPPATPAAVGPTTM (oxi)ASGGGAPM (oxi)A (184-212)							
cluster_no:5/member:10							
peak_no	m/z	rt	intensity	Hex	HexNAc	dHex	NeuAc
497	2881.321	61.9	302018	0	1	0	0
346	3043.372	61.06	572997	1	1	0	0
1985	3043.376	62.69	21129				
1102	3043.378	62.01	66605				
636	3084.399	60.83	191739	0	2	0	0
1970	3246.451	59.43	21498	1	2	0	0
879	3246.452	60.08	101782				
2698	3246.453	58.76	9019				
2361	3334.466	64.29	13970	1	1	0	1
596	3334.469	62.63	215796				
1677	3334.469	63.46	30550				
1041	3334.47	60.26	74799				
2550	3408.509	58.76	11121	2	2	0	0
2117	3537.548	60.35	18564	1	2	0	1
780	3537.549	61.58	123455				
1480	3537.549	59.21	38553				
282	3625.561	61.06	774921	1	1	0	2
1706	3625.563	63.29	29419				
1945	3625.566	64.73	22098				
431	3828.646	59.87	388762	1	2	0	2
1005	3845.674	59.87	81457	2	2	1	1

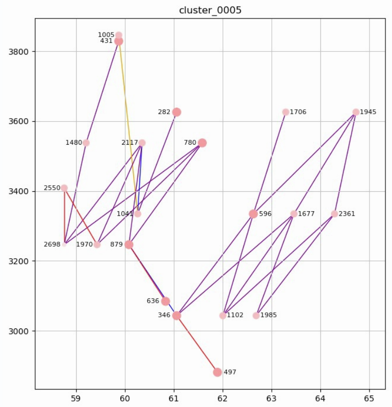

B DSSSGIGKTGQQPAKKRLNFGQTG

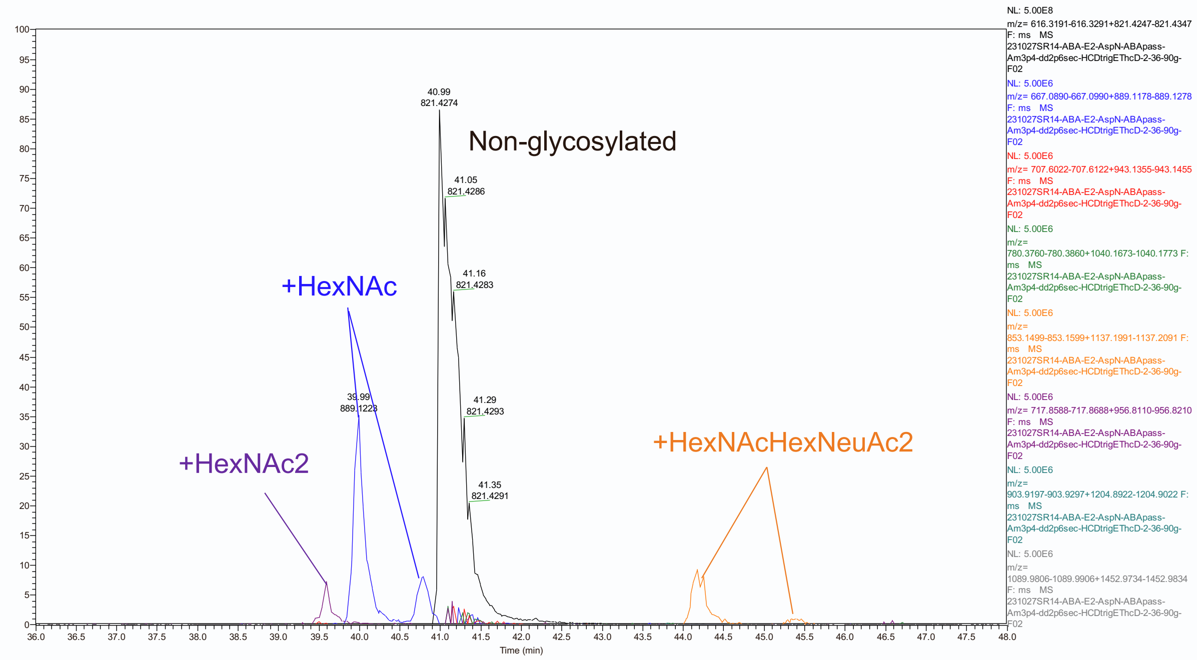

Figure S6. (A) Glycopeptide clusters assigned by GRable for D156–G177 and D184–A212 of the fractionated rAAV6. (B) XICs of D156–G177 modified with mucin-type *O*-glycan clusters. The signal of non-glycosylated peptide (*m/z* 821.4297 ± 0.005) is colored black, HexNAc (*m/z* 889.1228 ± 0.005) is blue, HexNAc2 (*m/z* 956.8160 ± 0.005) is purple and HexNAcHexNeuAc2 (*m/z* 1137.1941 ± 0.005) is orange.

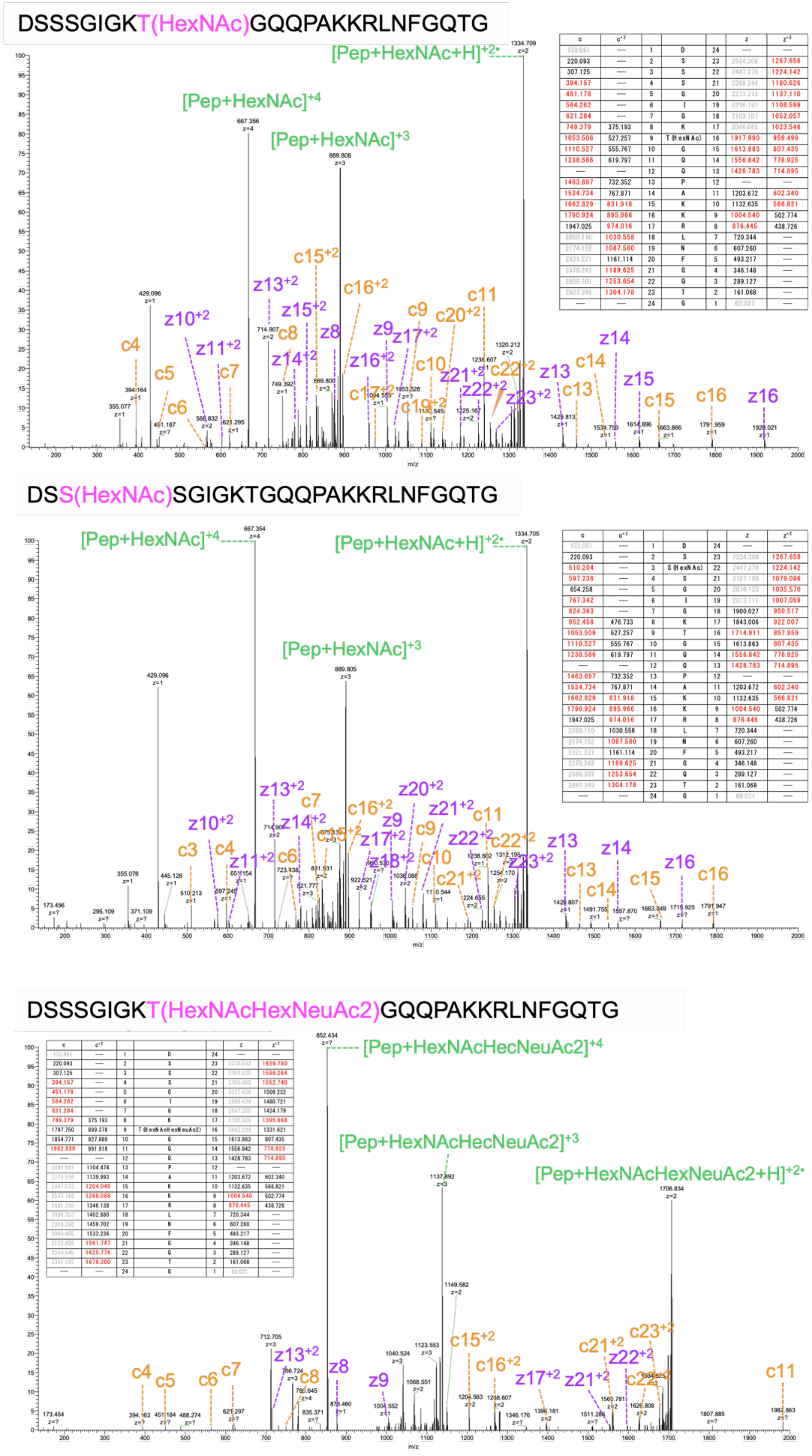

Figure S7. The EThcD mass spectra of D156–G177 modified with HexNAc at T162 (top), HexNAc at S156 (middle) and HexNAcHexNeuAc2 (bottom).

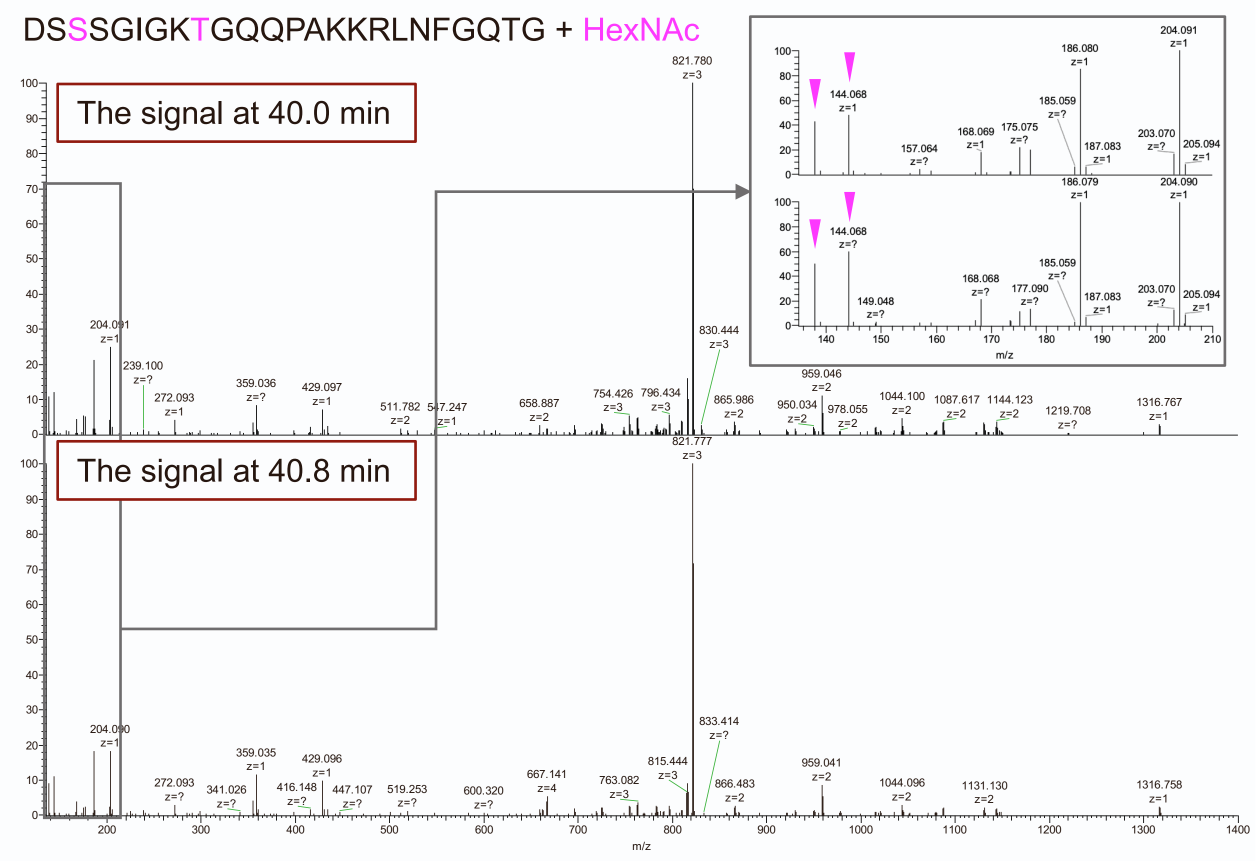

Figure S8. The HCD mass spectra of the signals at 40.0 min and 40.8min for D156–G177 modified with HexNAc. The signals *m/z* 138 and *m/z* 144 showed similar intensities.

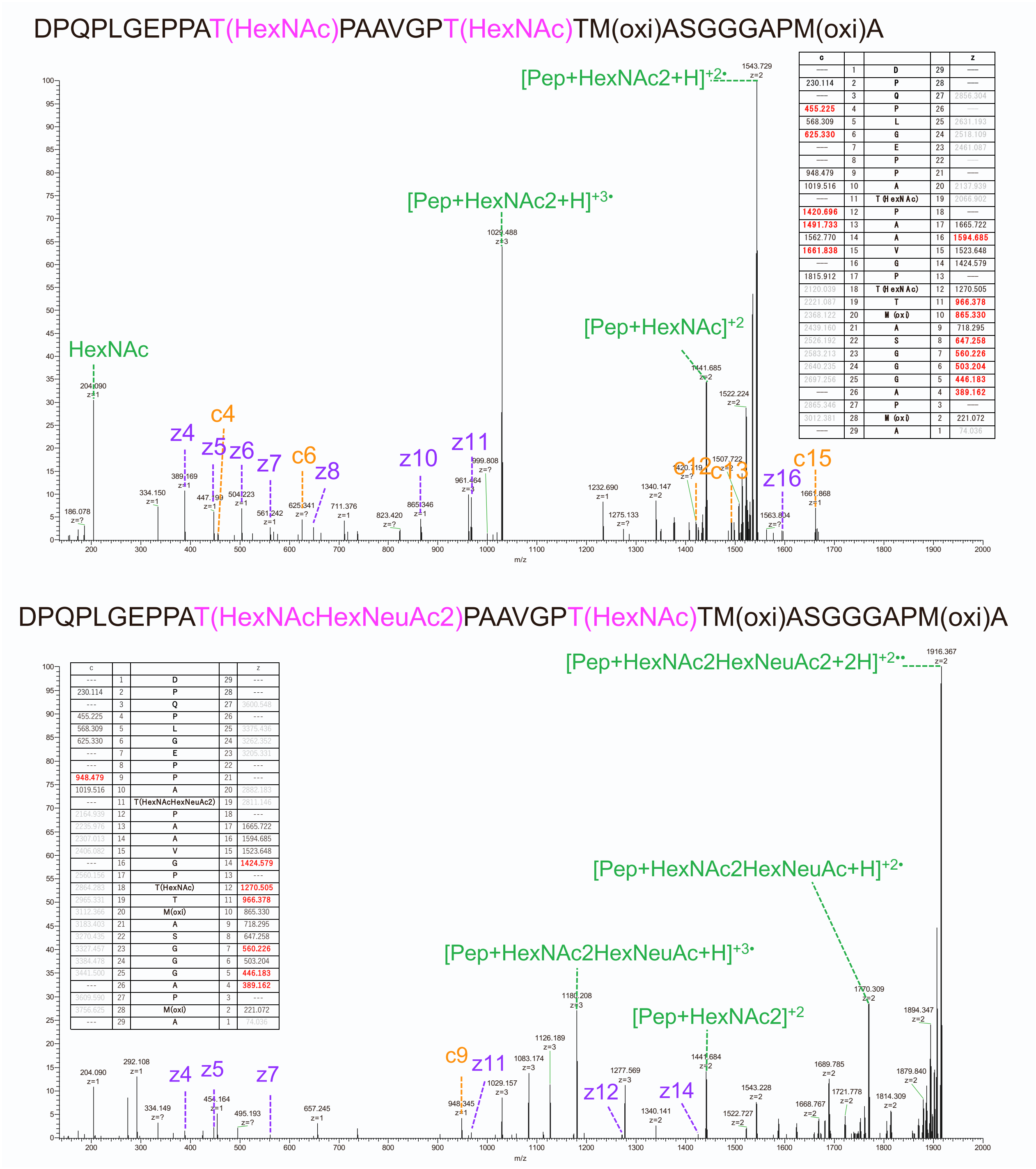

c				z
---	1	D	29	---
230.114	2	P	28	---
---	3	Q	27	2856.304
**455.225**	4	P	26	---
568.309	5	L	25	2631.193
**625.330**	6	G	24	2518.109
---	7	E	23	2461.087
---	8	P	22	---
948.479	9	P	21	---
1019.516	10	A	20	2137.939
---	11	T (HexNAc)	19	2066.902
**1420.696**	12	P	18	---
**1491.733**	13	A	17	1665.722
1562.770	14	A	16	**1594.685**
**1661.838**	15	V	15	1523.648
---	16	G	14	1424.579
1815.912	17	P	13	---
2120.039	18	T (HexNAc)	12	1270.505
2221.087	19	T	11	**966.378**
2368.122	20	M (oxi)	10	**865.330**
2439.160	21	A	9	718.295
2526.192	22	S	8	**647.258**
2583.213	23	G	7	**560.226**
2640.235	24	G	6	**503.204**
2697.256	25	G	5	**446.183**
---	26	A	4	**389.162**
2865.346	27	P	3	---
3012.381	28	M (oxi)	2	221.072
---	29	A	1	74.036

c				z
---	1	D	29	---
230.114	2	P	28	---
---	3	Q	27	3600.548
455.225	4	P	26	---
568.309	5	L	25	3375.436
625.330	6	G	24	3262.352
---	7	E	23	3205.331
---	8	P	22	---
**948.479**	9	P	21	---
1019.516	10	A	20	2882.183
---	11	T(HexNAcHexNeuAc2)	19	2811.146
2164.939	12	P	18	---
2235.976	13	A	17	1665.722
2307.013	14	A	16	1594.685
2406.082	15	V	15	1523.648
---	16	G	14	**1424.579**
2560.156	17	P	13	---
2864.283	18	T(HexNAc)	12	**1270.505**
2965.331	19	T	11	**966.378**
3112.366	20	M(oxi)	10	865.330
3183.403	21	A	9	718.295
3270.435	22	S	8	647.258
3327.457	23	G	7	**560.226**
3384.478	24	G	6	503.204
3441.500	25	G	5	**446.183**
---	26	A	4	**389.162**
3609.590	27	P	3	---
3756.625	28	M(oxi)	2	221.072
---	29	A	1	74.036

Figure S9. The EThcD mass spectra of D184–A212 modified with HexNAc at T194 and T201 (top), and HexNAcHexNeuAc2 at S194 and HexNAc at T201 (bottom).

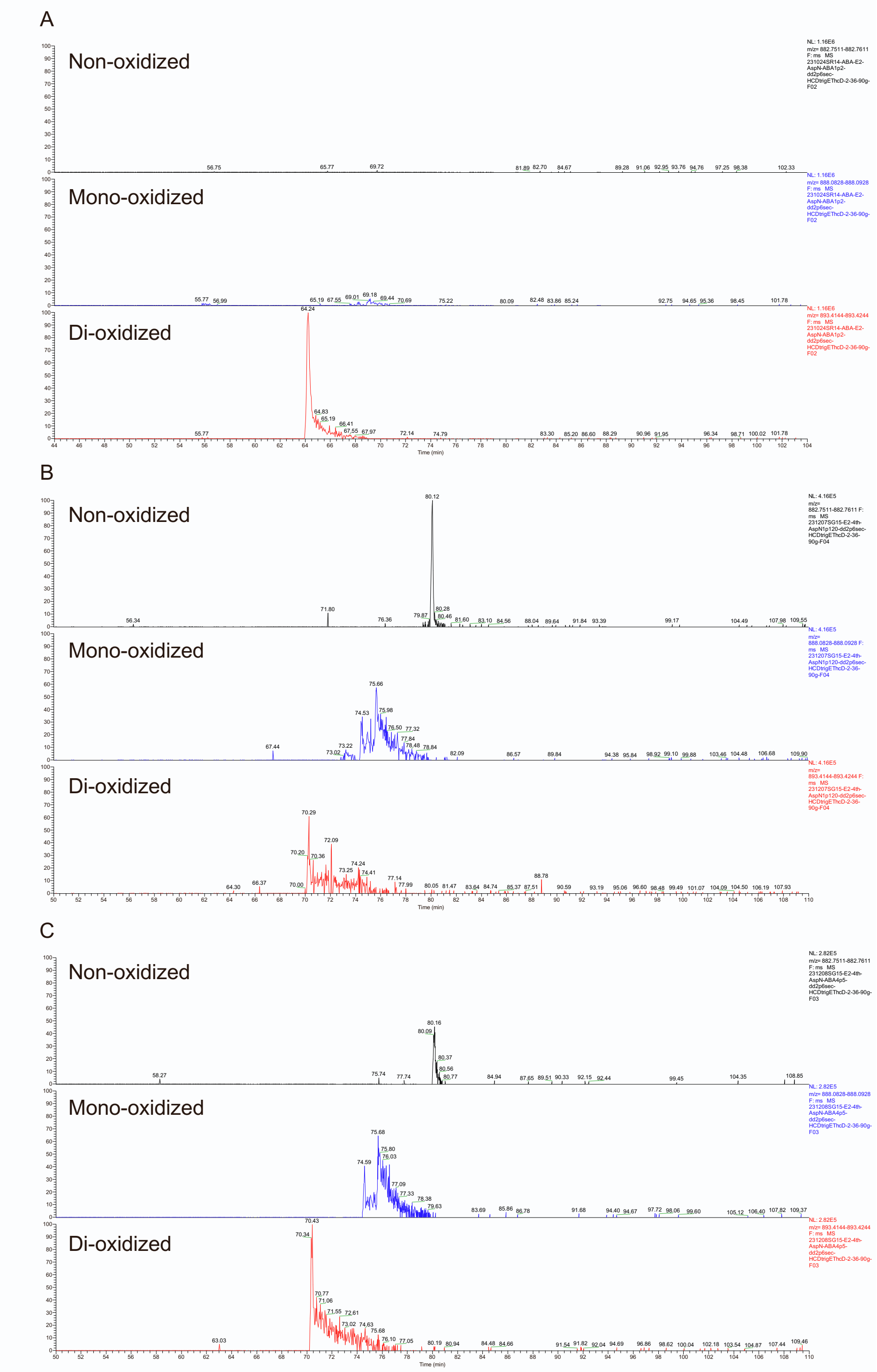

Figure S10. XICs of D184-A212 modified by non- (*m/z* 882.7561 ± 0.005; top), mono- (*m/z* 882.0878 ± 0.005; middle), and di-oxidized form (*m/z* 893.4194 ± 0.005; bottom) in (A) the ABA-bound fraction of the peptides digested from ABA-bound rAAV6 with the S-trap column, (B) the peptides digested from ABA-bound rAAV6 without the S-trap column, and (C) the peptides digested from ABA-bound rAAV6 with the S-trap column. The non-oxidized form was abundant for rAAV6 digested without the S-trap digestion, whereas the ratio of mono- and di-oxidized form to non-oxidized form increased after the S-trap digestion. We therefore suspect that some reagent used in the S-trap digestion may accelerate the oxidation of methionine.

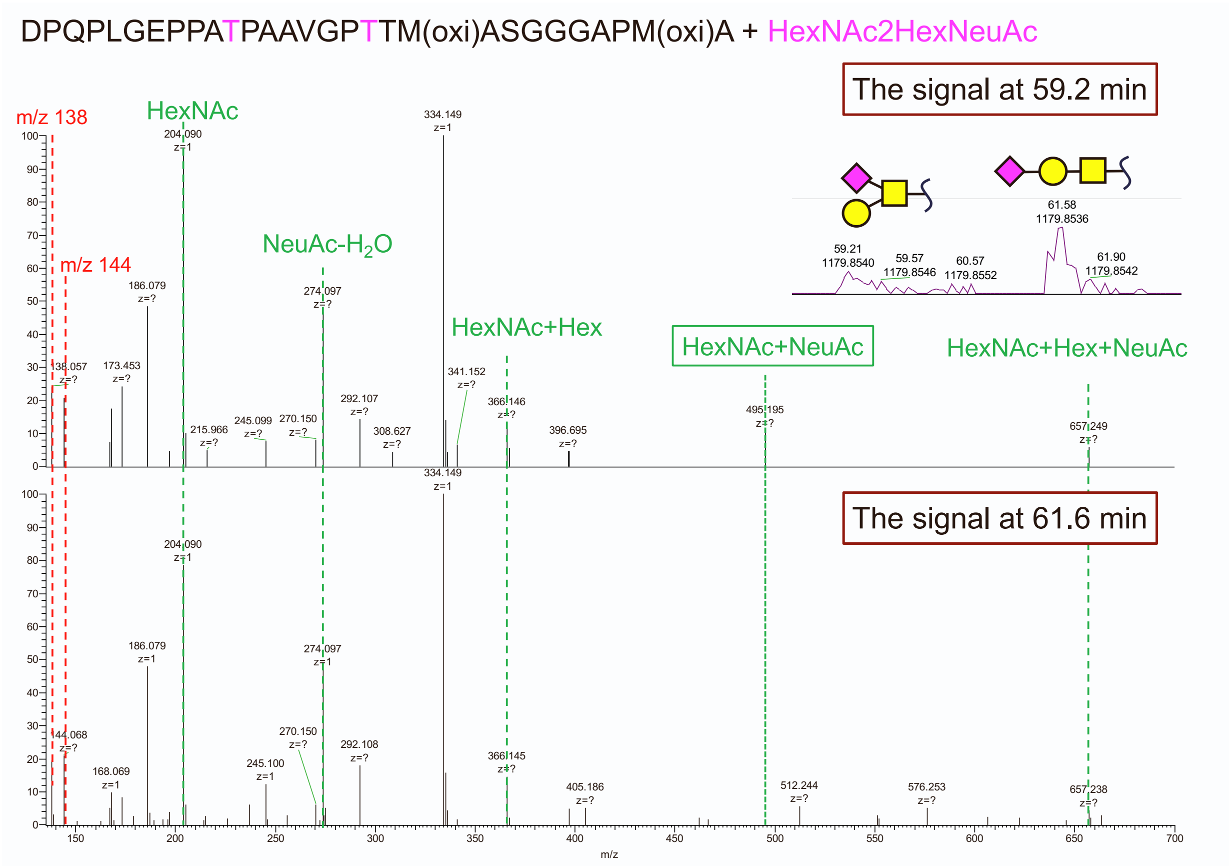

Figure S11. The HCD mass spectra of the signals at 59.2 min and 61.6 min for D184–A212 modified with HexNAc2HexNeuAc. The signals *m/z* 138 and *m/z* 144 showed similar intensities.

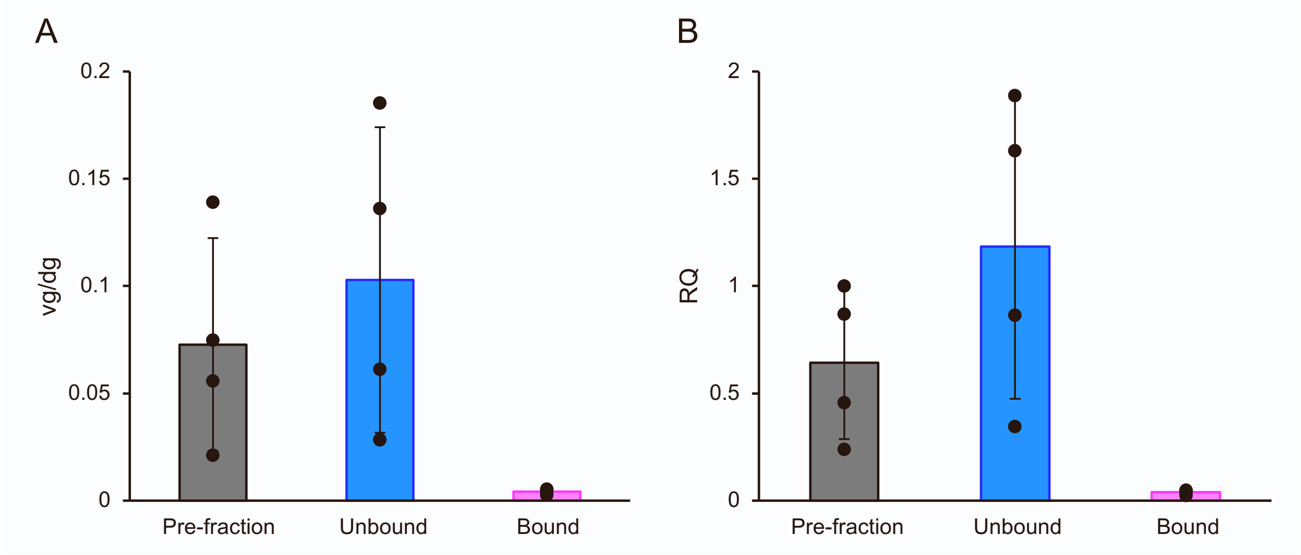

Figure S12. (A) rAAV6 genomes per mouse diploid genome (vg/dg) and (B) *hFIX* mRNA levels in liver tissue were determined using qPCR 8 weeks after rAAV6 administration. Individual points are shown in black circles, and error bars shows the SD value for n = 4.

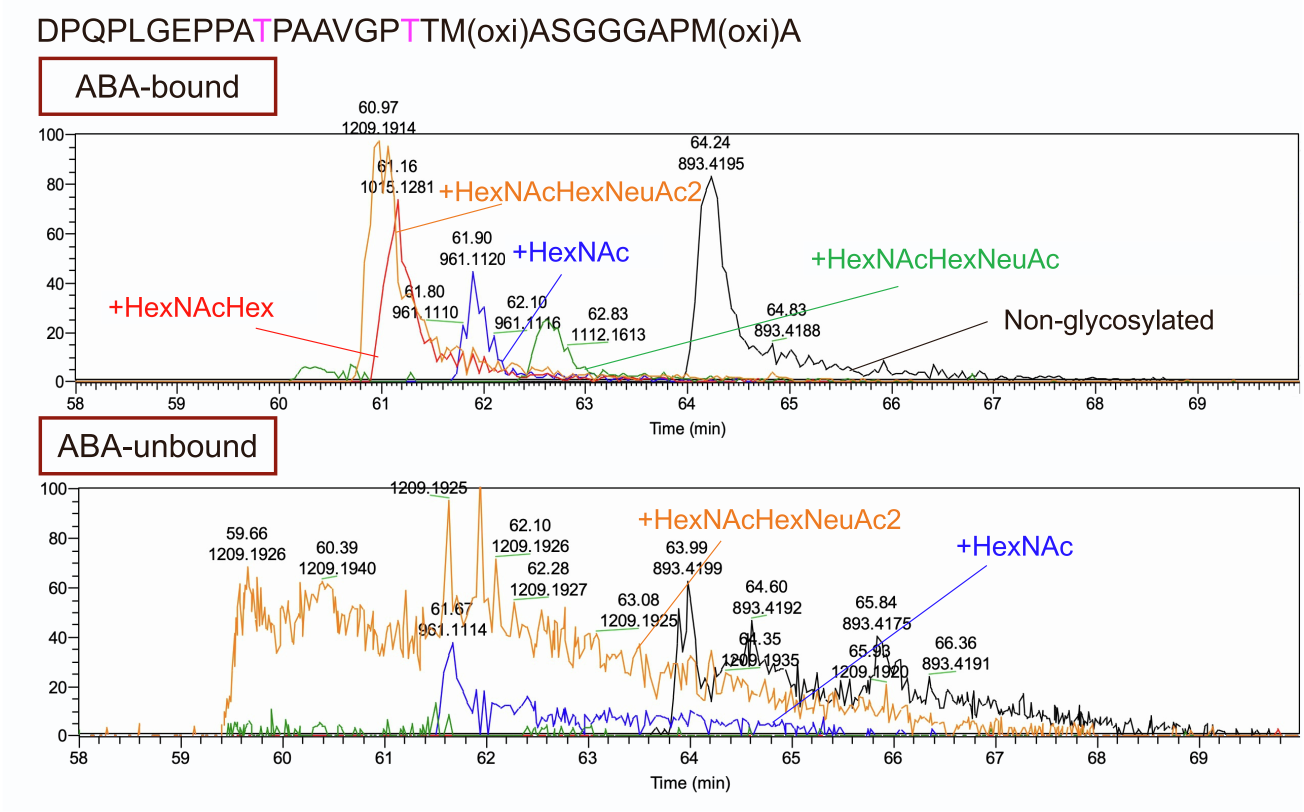

Figure S13. XICs of D184–A212 modified with mucin-type *O*-glycan clusters. The signal of non-glycosylated peptide (*m/z* 893.4194 ± 0.005) is colored black, HexNAc (*m/z* 961.1025 ± 0.005) is blue, HexNAcHex2 (*m/z* 1015.1231 ± 0.005) is red, HexNAcHexNeuAc (*m/z* 1112.1619 ± 0.005) is green, and HexNAcHexNeuAc2 (*m/z* 1209.1937 ± 0.005) is orange.
