# Supplementary material for: Glycosylation of recombinant adeno-associated virus serotype 6
Source: Mol Ther Methods Clin Dev. 2024 Apr 25;32(2):101256. doi: 10.1016/j.omtm.2024.101256 (PMC11107246; doi:10.1016/j.omtm.2024.101256)
Supplement: Document S2. Article plus supplemental information [file mmc2.pdf]

Molecular Therapy
Methods & Clinical Development
Original Article

Glycosylation of recombinant adeno-associated virus serotype 6

Yuki Yamaguchi,[1] Kentaro Ishii,[1] Sachiko Koizumi,[2,3] Hiroaki Sakaue,[4] Takahiro Maruno,[1,5] Mitsuko Fukuhara,[1,5] Risa Shibuya,[1] Yasuo Tsunaka,[1] Aoba Matsushita,[1] Karin Bandoh,[1] Tetsuo Torisu,[1] Chie Murata-Kishimoto,[2] Azusa Tomioka,[4] Saho Mizukado,[4] Hiroyuki Kaji,[6] Yuji Kashiwakura,[7,8] Tsukasa Ohmori,[7,8] Atsushi Kuno,[4] and Susumu Uchiyama[1]

[1]Department of Biotechnology, Graduate School of Engineering, Osaka University, 2-1 Yamadaoka, Suita, Osaka 565-0871, Japan; [2]GlycoTechnica Ltd., Yokohama, Japan; [3]Precision System Science Co. Ltd., 88 Kamihongo, Matsudo, Chiba 271-0064, Japan; [4]Molecular and Cellular Glycoproteomics Research Group, Cellular and Molecular Biotechnology Research Institute, National Institute of Advanced Industrial Science and Technology, Tsukuba, Ibaraki 305-8565, Japan; [5]U-Medico Inc., 2-1 Yamadaoka, Suita, Osaka 565-0871, Japan; [6]Institute for Glyco-core Research (iGCORE), Nagoya University, Furo-cho, Chikusa, Nagoya, Aichi 464-8601, Japan; [7]Department of Biochemistry, Jichi Medical University School of Medicine, 3111-1 Yakushiji, Shimotsuke, Tochigi 329-0498, Japan; [8]Center for Gene Therapy Research, Jichi Medical University, 3111-1 Yakushiji, Shimotsuke, Tochigi 329-0498, Japan

Glycosylation of biopharmaceuticals can affect their safety and efficacy. Glycans can occur on recombinant adeno-associated viruses (rAAVs) that are used for gene therapy; however, the types of glycans that attach to rAAVs are controversial. Here, we conducted lectin microarray analyses on six rAAV serotype 6 (rAAV6) preparations that were produced differently. We demonstrate that *O*-glycans considered to be attached to rAAV6 were recognized by *Agaricus bisporus* agglutinin (ABA) and that *N*-glycans were detected in rAAV6 purified without affinity chromatography. Liquid chromatography-tandem mass spectrometry (LC-MS/MS) analysis showed that the *N*-glycans detected in rAAV6 were derived from host cell proteins. A combination of ABA-based fractionation and LC-MS/MS revealed that rAAV6 was *O*-glycosylated with the mucin-type glycans, *O*-GalNAc (Tn antigen), and mono- and di-sialylated Galβ1-3GalNAc (T antigen) at S156, T162, T194, and T201 in viral protein (VP) 2 and with *O*-GlcNAc at T242 in VP3. The mucin-type *O*-glycosylated rAAV6 particles were 0.1%–1% of total particles. Further physicochemical and biological analyses revealed that mucin-type *O*-glycosylated rAAV6 had a lower ratio of VP1 to VP2/VP3, resulting in a lower transduction efficiency both *in vitro* and *in vivo* compared with rAAV6 without mucin-type *O*-glycans. This report details conclusive evidence of rAAV glycosylation and its impact on rAAV-based therapeutics.

INTRODUCTION

Recombinant adeno-associated viruses (rAAVs) are promising vehicles for target genes in gene therapy because of the low immunogenicity of wild-type AAV in humans; broad tissue tropism, including the central nervous system; and long-term expression in non-dividing cells.[1–3] rAAVs are composed of a single-stranded DNA (ssDNA) coding a gene of interest inside a capsid that is made of 60 subunits, mainly viral protein (VP) 1, VP2, and VP3, and a minor component, $VP3_{variant}$.[4,5]

Post-translational modifications (PTMs) can alter the structure, dynamics, and, ultimately, the function of proteins. For rAAVs, ubiquitination, phosphorylation, SUMOylation, acetylation, oxidation, and deamidation PTMs have been identified. The levels of these modifications vary among different serotypes and because of the cell line used for production, such as Sf9 and HEK293; lot-to-lot differences; and storage conditions.[6–9] Almost all of the methionine-truncated VP1 and VP3 N-termini are generally acetylated,[5] and the introduction of mutations that suppress the acetylation reduces transduction efficiency.[10] Tyrosine phosphorylation facilitates the ubiquitination of rAAV capsids, which is followed by proteasome degradation.[11] Interestingly, deamidation of specific asparagine residues can reduce transduction efficiency and increase immunogenicity.[12,13] Although the glycosylation of AAVs has also been reported, the types of glycans involved are controversial; two studies have reported that *N*-glycans attaches to AAV serotype 2 (AAV2),[6,14] while another study has shown that introduction of glycosylation into T14N mutants increases transduction efficiency.[15] However, another study has reported that AAV2 is not glycosylated.[16] Similarly, several studies have reported AAV8 modified with *N*-glycans,[6,14,17] whereas another study reports only *O*-Linked *N*-Acetylglucosamine (*O*-GlcNAc) attached to AAV8.[18]

If rAAV is naturally glycosylated during the manufacturing process, then it is necessary to consider whether the glycosylation is a critical quality attribute that influences transduction efficiency and immunogenicity. Studies on antibodies have reported immunogenic glycan structures; galactose-α1,3-galactose attached to cetuximab is

Received 8 February 2024; accepted 22 April 2024;
https://doi.org/10.1016/j.omtm.2024.101256.

Correspondence: Susumu Uchiyama, PhD, Department of Biotechnology, Graduate School of Engineering, Osaka University, 2-1 Yamadaoka, Suita, Osaka 565-0871, Japan.
E-mail: suchi@bio.eng.osaka-u.ac.jp

This is an open access article under the CC BY license (http://creativecommons.org/licenses/by/4.0/).

Table 1. rAAV6 preparations used for lectin microarray analyses

Name	Supplier	Genome size (bases)	Cell line	Cell type	Affinity	Density gradient
Sample 1	SR	3,342	HEK293T	adherent	yes	iodixanol
Sample 2	SG	2,521	HEK293T	adherent	yes	CsCl
Sample 3	TK	2,521	HEK293T	adherent	yes	CsCl
Sample 4	VB	2,521	HEK293T	adherent	no	CsCl
Sample 5	VB	2,521	HEK293T	adherent	no	CsCl
Sample 6	in-house	4,133	HEK293	suspension	yes	CsCl

Samples 1–5 were purchased from four different suppliers. SR, Sirion Biotech, SG, SignaGen Laboratories, TK, Takara Bio Inc., and VB, Vector Builder. Samples 4 and 5 were different lot numbers. Samples 6 was manufactured in house.

recognized by immunoglobulin E (IgE) and induces anaphylaxis,[19] and, similarly, *N*-glycolylneuraminic acid (NeuGc) induces anti-NeuGc-IgG to generate immune complexes.[20] Such glycans often attach to proteins during production in non-human mammalian cell lines and/or animal sera; therefore, it is unlikely that rAAVs produced in the human cell HEK293 and HEK293T are modified with those glycans. However, if the glycans are attached to the rAAV capsids, they may act as a carbohydrate epitope to produce antibodies and cause immunogenicity. The difference in rAAV8 glycosylation between human and baculovirus-Sf9 production platforms was that Sf9-produced rAAV8 has one more *O*-GlcNAc modification at T633.[18] Other modifications, such as acetylation, methylation, and phosphorylation, have been observed at higher levels in Sf9-produced rAAV8 compared with human cell-produced rAAV8.[9,18]

Liquid chromatography-tandem mass spectrometry (LC-MS/MS) is an effective technique for glycopeptide analysis. It can determine both attached glycan structures and modification sites from diagnostic glycan fragment ions in collision-induced dissociation (CID)/electron transfer dissociation (ETD) fragmentation spectra produced by the fragmentation of glycopeptides.[21,22] However, only one study has used mass spectrum data containing diagnostic glycan fragment ions to determine the glycan modification sites.[18] Here, we studied rAAV6 to explore whether AAVs are glycosylated and, if so, what types of glycans are attached to the capsid and whether any glycans are associated with immunogenicity by combining LC-MS/MS analysis with a lectin-based fractionation technique. The lectin microarray is a highly sensitive and high-throughput analytical method to evaluate glycan profiles of proteins in solution that is based on the ability of lectin to bind specific glycan structures.[23,24] Each lectin detects distinctive glycan structures;[25] therefore, glycan profiles can be estimated from the detected patterns. Lectin microarray analysis was performed on six rAAV6 preparations produced differently to find the lectins that can capture the glycans attached to rAAV6. We then performed peptide mapping to screen for rAAV6 glycosylation recognized by lectins. Subsequently, we fractionated rAAV6 using *Agaricus bisporus* agglutinin (ABA), which potentially recognizes glycans attached to rAAV6 particles, and conducted LC-MS/MS analyses of the fractionated rAAV6 to more precisely identify glycans in rAAV6. The ABA-bound rAAV6 particles accounted for only 0.1%–1% of total particles but were successfully enriched using ABA-immobilized beads, leading us to observe mucin-type *O*-glycan clusters attached to rAAV6 capsid particles. Moreover, we evaluated the impact of mucin-type *O*-glycosylation on particle size distribution, VP ratio, and *in vivo* and *in vitro* transduction efficiencies. These studies showed that mucin-type *O*-glycosylated rAAV6 had a lower VP1 ratio, resulting in lower transduction efficacy both *in vitro* and *in vivo*, than rAAV6 without mucin-type *O*-glycans. This study provides conclusive proof of the glycosylation of rAAVs, which is important for the consideration of critical quality attributes of rAAV-based therapeutics.

RESULTS

Lectin microarray analyses of rAAV6 glycan profiles

Lectin microarray analysis was conducted for six rAAV6 preparations produced by different methods (Table 1). As shown in Figure 1A, samples 1–3 had similar glycan profiles with high signal intensities for *Agrocybe cylindracea* galectin (ACG), ABA, and *Artocarpus integrifolia* lectin (Jacalin). Samples 4 and 5 had relatively higher signal intensities for lectins from *Sambucus nigra* agglutinin (SNA) to *Datura stramonium* agglutinin (DSA) and *Tulipa gesneriana* lectin_I (TxLC_I) compared with those of samples 1–3. Sample 6 showed relatively low signal intensities for all lectins, except *Maackia amurensis* hemagglutinin II (MAH).

Principal-component analysis (PCA), which can extract features from multivariate data,[26] was applied to lectin microarray data from three different concentrations of each sample to investigate the difference in glycan profiles of the analyzed rAAV6. As shown in Figure 1B, the PCA score plot shows separation as a function of principal component (PC) 1. Samples 1–3 and 6 were placed in the left group and samples 4 and 5 in the right group. In the PCA loading plot, lectins recognizing *N*-glycans, such as TxLC_I, *Phaseolus vulgaris* erythroagglutinin (PHA(E)) and DSA, localized to the slightly positive side of PC1, whereas ABA and Jacalin (which can commonly bind to *O*-glycan core 1), MAH (which can bind to α2,3-sialylated Galβ1-3GalNAc in *O*-glycans), and ACG (which can bind to α2,3 sialic acid), localized to the negative side (Figure 1C).[25,27–29] Notably, ABA was located on the strongly negative side, which means that the relative intensity of the signal associated with the interaction of rAAV6 and ABA to the *N*-glycan-related signals is the strongest determining factor for the classification. Samples 1–3 and 6 in the

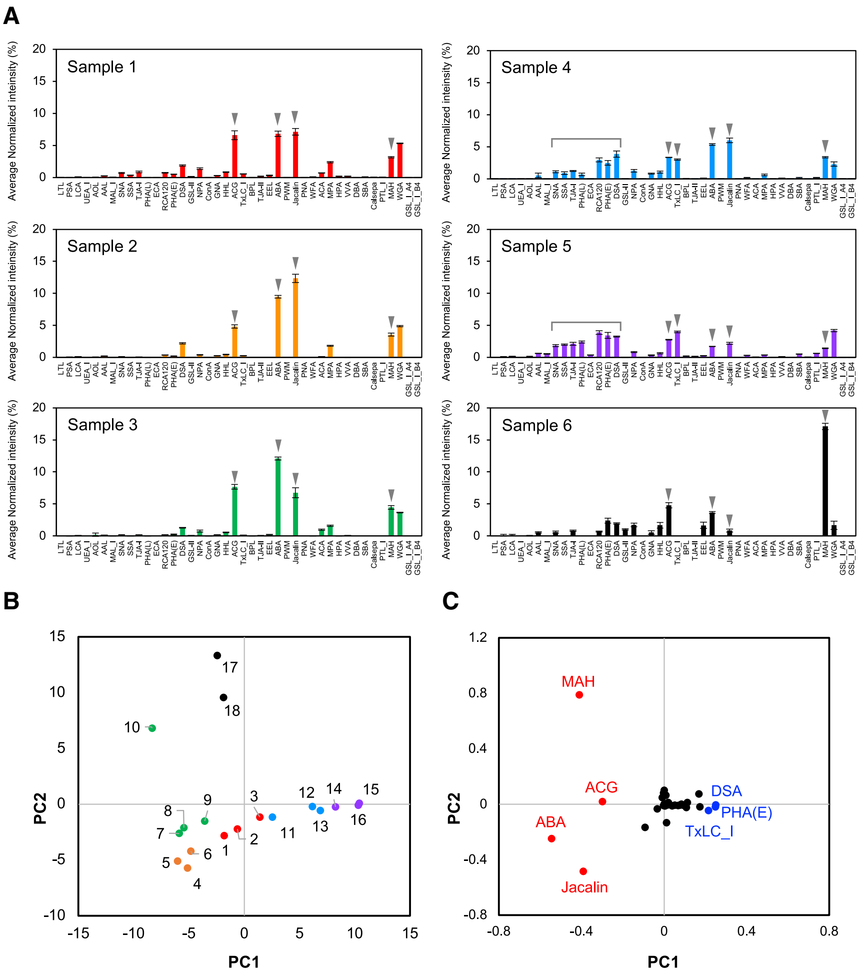

Figure 1. Lectin microarray analyses of rAAV6 produced by different methods

(A) Representative glycan profiles of six rAAV6 preparations produced by different methods. The plot shows the mean-normalized signal intensities with standard diviation (SD) from triplicate spots of the 42 lectins for 2.5×10^9 vg. The signals of *O*-glycan recognition lectins, ACG, ABA, Jacalin, and ACG, are indicated for all samples; *N*-glycan recognition lectins are additionally indicated for samples 4 and 5. (B and C) PCA score (B) and loading plot (C) based on the glycan profiles obtained by lectin microarray analyses. Different samples are represented with different colors. The colors of the dots in the PCA score plot correspond to the bars in the glycan profiles of each sample. The number of each dot corresponds to the sample information used for the PCA analysis shown in Table S1. Representative *N*-glycan-recognized lectins are shown in blue and *O*-glycan-recognized lectins in red in the PCA loading plot.

left group were purified by affinity chromatography, whereas samples 4 and 5 were only purified by density gradient ultracentrifugation. These results indicate that the method used to purify rAAV6 affects its glycan profile; rAAV6, which is highly purified by affinity chromatography and density gradient ultracentrifugation, contains mainly *O*-glycans with sialic acids, whereas moderately purified rAAV6 not purified by affinity chromatography has a higher content of *N*-glycans. Therefore, the *N*-glycans detected in rAAV6 are likely to be mainly derived from host cell proteins (HCPs).

Proteome analysis of sample 4 showed that rAAV6 was contaminated with several proteins derived from bovine serum, possibly from the culture medium, and from the human HEK293T cells used for production (Table S2). Human galectin 3-binding protein, which is known as M2BP and a possible major AAV6 hitchhiker protein,[30] was also identified. The M2BP content in samples 4 and 5 was much higher than that in samples 1–3 (Figure S1A). Sialidase treatment of samples 4 and 5 caused the M2BP band to shift, and the peptide derived from M2BP in sample 4 was identified with $HexNAc_6Hex_7FucNeuAc_2$ modification (Figures S1B and S1C); therefore, M2BP is a protein highly glycosylated with sialic acids, as seen in our previous studies.[31,32] These results agree with the hypothesis that the higher intensity of the *N*-glycan-related signals in samples 4 and 5 is caused by the detection of *N*-glycans attached to contaminating HCPs. Therefore, rAAV6 particles are potentially *O*-glycosylated with sialic acids, which are recognized by ABA, Jacalin, MAH, and ACG, and, if insufficiently purified, co-exist with *N*-glycosylated HCPs.

Analysis of rAAV6 glycopeptide by MS

Lectin microarray analysis indicated that rAAV6 particles were modified with *O*-glycans containing sialic acids. We then applied glycopeptide mapping using LC-MS/MS with CID fragmentation of highly purified rAAV6 (samples 1–3) to identify the glycans attached to rAAV6 capsids that are recognized by the lectin. We identified *N*-acetylhexosamine (HexNAc) modification to V239–R245 across all samples, indicating that the modification is independent of the upstream processing method (Figures 2A, 2C, and S2A). *m/z* 138 was observed, but *m/z* 144 was not observed in CID spectra; therefore, the identified HexNAc was assigned as *O*-GlcNAc.[33] By comparing the MS area, the *O*-GlcNAc glycosylated peptide was estimated to be 0.05% of total glycosylated and unmodified peptides (Figure S2B). Unfortunately, the *O*-GlcNAc binding site was not determined because the V239–R245 peptide contains three threonine residues and one serine residue, which are potential *O*-glycosylation sites. The $HexNAcHexNeuAc_2$ modification at T162 was detected only in sample 3 (Figures 2A and 2B), which might be because of the difference in the glycosylation level in each sample, since our lectin microarray analysis indicated that samples 1–3 have the same glycosylation profiles (Figure 1A). Considering that samples 1–3 were produced by the same cell line and purification methods but were from different suppliers, as shown in Table 1, this *O*-glycan may therefore be affected by production conditions; i.e., medium and culture

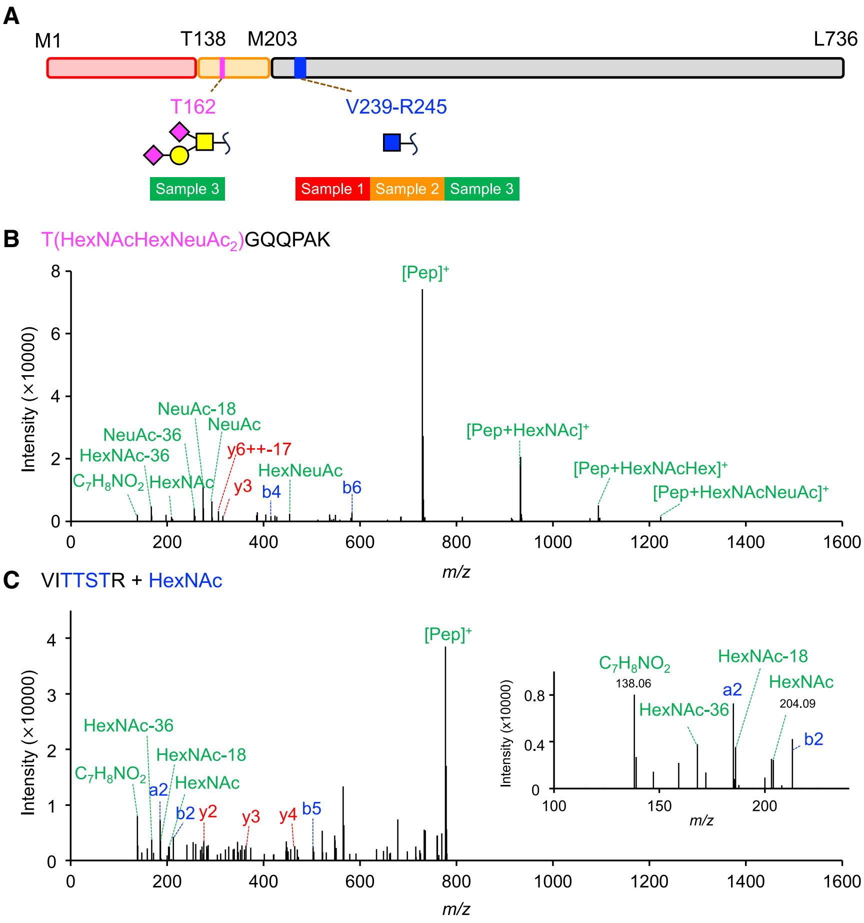

Figure 2. Glycopeptide analysis of rAAV6 by LC-MS/MS with CID fragmentation

(A) Schematic of identified glycans and glycosylation sites. *O*-GlcNAc is depicted as a blue square, *O*-GalNAc as a yellow square, galactose as a yellow circle, and sialic acid as a magenta diamond. (B) The CID mass spectrum of T162–K168 modified with $HexNAcHexNeuAc_2$. (C) The CID mass spectrum of V239–R245 modified with HexNAc.

conditions. *O*-GlcNAc is generally not modified with sialic acid;[34] therefore, $HexNAcHexNeuAc_2$ at T162 is considered an *O*-GalNAc derivative, di-sialylated Galβ1-3GalNAc (T antigen). Therefore, in lectin microarrays, ABA, Jacalin, MAH, and ACG are considered to recognize this *O*-glycan of rAAV6. Although MS searches also included *N*-glycan modifications, no *N*-glycans attached to unique rAAV6 peptides were detected in any of the rAAV6 samples.

Fractionation of rAAV6 particles using ABA-immobilized beads

To further characterize rAAV6 glycosylation, we used sample 3 and fractionated rAAV6 particles with *O*-glycans by ABA capture, which potentially recognizes the *O*-glycans attached to rAAV6 capsids. ABA-immobilized magnetic beads were incubated with rAAV6. After the beads' absorption with a magnet, the supernatant was collected as the unbound fraction. The nonspecific bound component was washed out by 0.5 M glycine and 1% poloxamer-188 in Tris-buffered saline (TBS) as the wash fraction, and the bound component was eluted by saccharides that compete with the ABA binding glycoproteins as the bound fraction. When the amount of rAAV6 applied was 1.3×10^7 vector genomes (vg), the bands corresponding to VP1, VP2, and VP3 observed by western blotting were only present in the unbound fraction, and there appeared to be no fraction that bound to ABA. However, when the initial amount of rAAV6 was increased 100-fold, a band corresponding to VP3 appeared (Figure 3A). This indicated that ABA-bound rAAV6 particles accounted for only approximately 1% of total particles. The ABA signals disappeared in the unbound rAAV6 fraction (Figures 3B and S3); therefore, we considered that the rAAV6 particles, which have *O*-glycans recognized by ABA, were successfully captured by the ABA-immobilized beads. We therefore conducted a large-scale purification using the same method as that used for small-scale fractionation. When 2.1×10^{14} vg of sample 2 was applied on to the beads, the total vg of the unbound, washed, and bound fractions was calculated by droplet digital PCR (ddPCR). The recovery rate was 46%, probably because of rAAV6 lost by adsorption to the tools during fractionation. The rAAV6 particles firmly captured by ABA accounted for 0.1% of the total particles (Figure 3C); this is based on the assumption that ABA-bound and unbound rAAV6 particles have the same adsorption properties on the tools. The amount of ABA-bound particles determined by western blotting showed a similar value of 0.5% of the total particles. These results indicate that sample 2 was modified with a lower level of *O*-glycans bound to ABA than sample 3 (namely, *O*-GalNAc derivatives), which is supported by the fact that the glycan was only detected in sample 3 (Figures 2A and 2B). Therefore, rAAV6 is considered to be commonly modified with ABA-bound *O*-glycans, with modification levels varying from 0.1% to 1%.

MS analysis of glycopeptide in ABA-fractionated rAAV6

Although CID fragmentation is useful for exploring the glycosylation of proteins, it mainly induces the fragmentation of glycan structures but with limited fragmentation of peptide backbones, making it difficult to identify the amino acid residues that are glycosylated. We therefore applied LC-MS/MS with hybrid electron-transfer/higher-energy collision dissociation (EThcD) fragmentation for glycopeptide analysis of rAAV6 particles after fractionation by ABA. However, the sample amount produced by the small-scale purification was sufficient for ABA-unbound fraction analysis, whereas ABA-bound

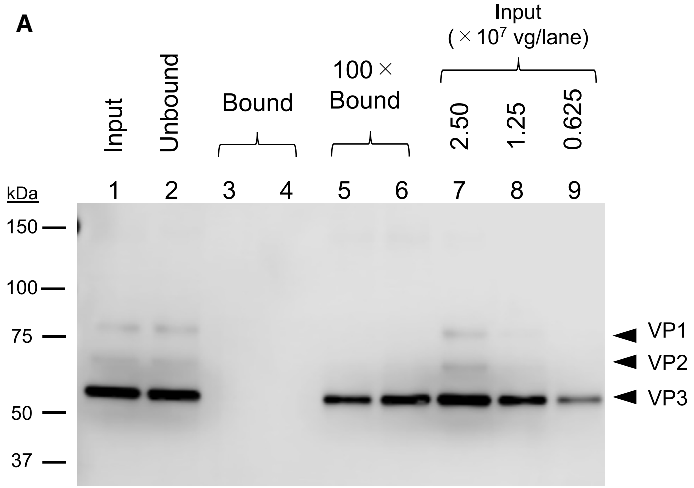

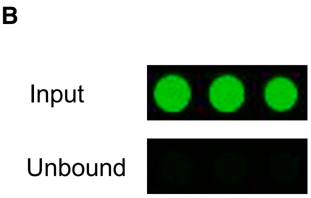

C

	Amount (vg)	Ratio (%)
Unbound	9.4×10^{13}	97.4
Wash	2.4×10^{12}	2.4
Bound	1.2×10^{11}	0.1

Figure 3. Fractionation of rAAV6 using ABA-immobilized beads

(A) Western blot of rAAV6 purified by ABA at a small scale. Untreated rAAV6 applied at 1.3×10^7 vg is "Input," rAAV6 collected from supernatant after incubation is "Unbound," and rAAV6 eluted from ABA-immobilized beads is "Bound." The result of the elution fraction for 1.3×10^9 vg is shown as "100 × Bound". (B) The ABA signals detected by lectin microarray for 2.5×10^{10} vg rAAV6 samples as described for western blotting. (C) The total vg of each component after the fractionation of rAAV6. rAAV6 not bound to ABA is "Unbound," non-specifically bound to ABA is "Wash," and bound to ABA is "Bound." The total vg of each fraction was calculated by ddPCR.

fraction analysis was difficult even after the large-scale purification because of the low abundance of glycopeptides. We therefore optimized the sample treatment of the post-fractionation and re-fractionated the peptides digested from the ABA-bound fraction when 1×10^{13} vg of sample 1 was applied, as shown in Figure S5.

The ABA-unbound fraction of rAAV6 was digested with Asp-N and Lys-C on an S-trap column instead of trypsin to generate relatively longer peptides, which are suitable for EThcD fragmentation. The obtained MS spectra were analyzed using the glycan analysis software Byonic. As shown in Figures 4A and S4, based on the EThcD fragmentation pattern and an *m/z* intensity ratio of 138–144 for the higher-energy collision dissociation (HCD) fragmentation pattern, only the HexNAc modification, *O*-GlcNAc, was identified at T242 in the unbound fraction of rAAV6. This was consistent with the rAAV6 pre-fraction analysis that showed T241, T242, S243, or T244 to be potential modification sites for *O*-GlcNAc (Figures 2A and 2C).

The ABA-bound fraction of rAAV6 was also digested with Asp-N on an S-trap column after ethanol precipitation to remove the surfactants and competitive saccharides in the elution buffer. We then incubated the digested peptides with ABA-immobilized beads and eluted the ABA-bound peptides with GalNAc solution. The ABA-unbound peptides were further enriched for glycopeptides with Amide-80 to avoid ion suppression by non-glycopeptides. The unbound and bound peptides were analyzed by LC-MS/MS with EThcD fragmentation, and the obtained spectral data were analyzed using Mascot and Byonic. Byonic searches of the ABA-unbound peptides identified HexNAc modification to D154–G177 and HexNAcHexNeuAc2 modification to D184–A212. For in-depth analysis of the identified glycopeptides, we performed GRable analysis on the peptides D154–G177 and D184–A212 for the MS results of both the unbound and the bound peptides. GRable, recently released in the GlyCosmos portal by Nagai-Okatani et al.,[35] can predict glycan composition on peptides from MS1 and retention time, unlike MS/MS spectrum-based searches in Byonic. GRable predicted glycosylation clusters on D154–G177 of the unbound peptides and on D184–A212 of the bound peptides, including additional glycan structures that were not identified by Byonic (Figures 4A, 4B, and S6). The ion chromatograms were then extracted for the signals of each peptide that showed glycan structures identified in both GRable results. The peaks of both HCD and EThcD spectra for each signal were then assigned.

For D154–G177, the EThcD spectra clearly identified the HexNAc modification at S156 or T162 and the $HexNAcHexNeuAc_2$ modification at T162 (Figure S7). The identified HexNAc was assigned as *O*-GalNAc (Tn antigen), and $HexNAcHexNeuAc_2$ was assigned as a mucin-type *O*-glycan, di-sialylated Galβ1-3GalNAc, based on the HCD spectra (Figure S8). Unfortunately, the signal to confirm the modification site at 44.5 min was derived from poor fragmentation; therefore, we presumed that the peptide was modified di-sialylated Galβ1-3GalNAc at S156 because the elution time interval between the signal of the peptide with *O*-GalNAc at T162 and that with *O*-GalNAc at S156 was the same as that between the signal of the peptide with di-sialylated Galβ1-3GalNAc at T162 and the signal at 44.5 min. The glycosylation of T162 was consistent with the pre-fractionation result, as shown in Figures 2A and 2B. Although MS/MS spectra were not obtained, the signal for the peptide with two HexNAc residues observed at 39.6 min could be assigned as the peptide that was glycosylated simultaneously at both S156 and T162. For D184–A212, EThcD clearly identified two glycosylation sites: T194 modified with

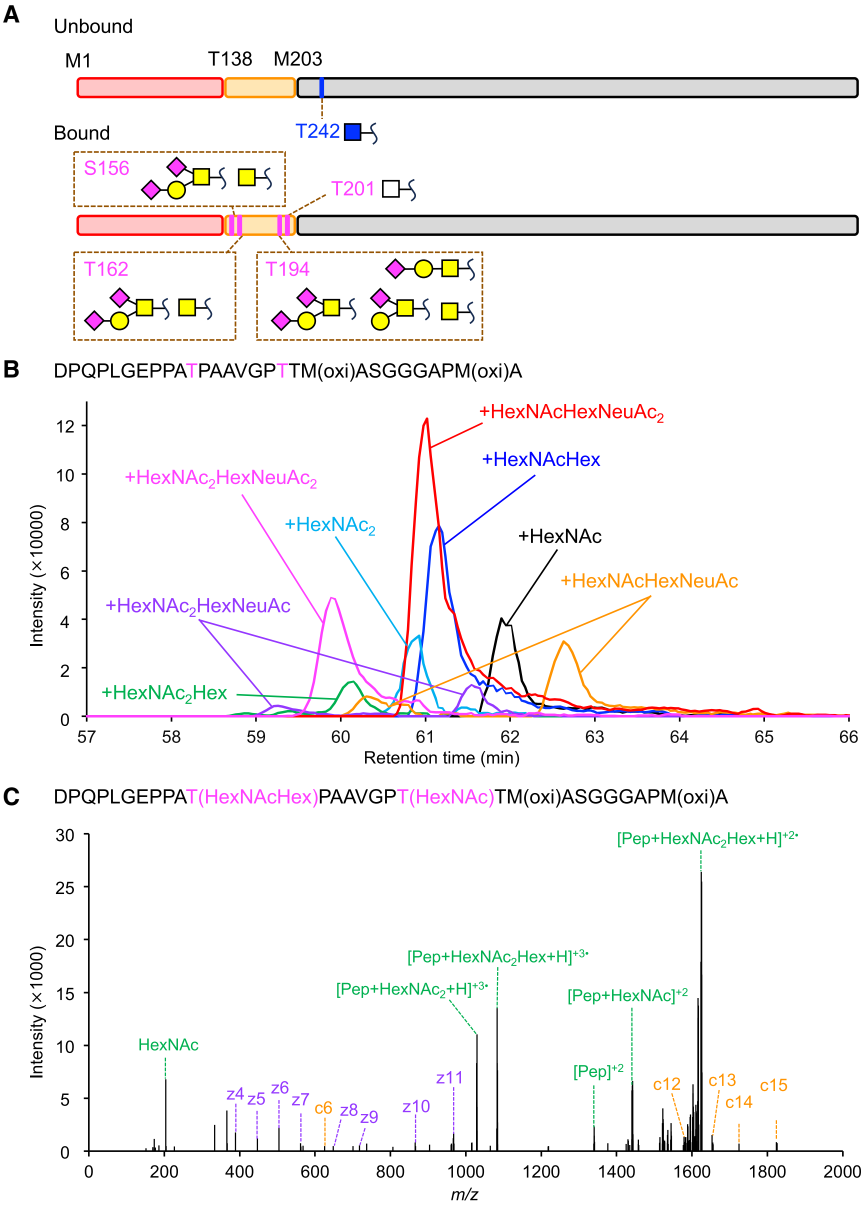

Figure 4. Glycopeptide analysis of rAAV6 by LC-MS/MS with hybrid electron-transfer/higher-energy collision dissociation fragmentation

(A) Schematic of identified glycans and glycosylation sites. *O*-GlcNAc is depicted as a blue square, *O*-GalNAc as a yellow square, galactose as a yellow circle, sialic acid as a magenta diamond, and HexNAc as a white square. (B) Extracted ion chromatograms of D184–A212 modified with two oxidation and mucin-type *O*-glycan clusters. The signal of peptide with HexNAc (*m/z* 961.1129 ± 0.005) is colored black, HexNAcHex (*m/z* 1015.1305 ± 0.005) blue, $HexNAc_2$ (*m/z* 1028.8060 ± 0.005) red, $HexNAc_2Hex$ (*m/z* 1082.8236 ± 0.005) green, HexNAcHexNeuAc (*m/z* 1112.1623 ± 0.005) orange, $HexNAc_2HexNeuAc$ (*m/z* 1179.8554 ± 0.005) purple, $HexNAcHexNeuAc_2$ (*m/z* 1209.1941 ± 0.005) red, and $HexNAc_2HexNeuAc_2$ (*m/z* 1276.8872 ± 0.005) magenta. (C) The EThcD mass spectrum of D184–A212 modified with $HexNAc_2Hex$.

mucin-type *O*-glycans, HexNAc as *O*-GalNAc and HexNAcHex as Galβ1-3GalNAc, and $HexNAcHexNeuAc_2$ as di-sialylated Galβ1-3GalNAc and T201 modified with HexNAc (Figures 4B, 4C, and S9). Two methionine residues were mostly oxidized in the analyzed peptide. The peptide was not oxidized when the ABA-bound rAAV6 was digested without the S-trap column, indicating that the oxidation occurred during the S-trap digestion step and ABA fractionation after digestion, as shown in Figure S10. The glycosylation sites of the peptides modified with HexNAcHexNeuAc or $HexNAc_2HexNeuAc$ detected at two different retention times could not be determined because of poor EThcD spectra. T194 was identified with *O*-GalNAc derivative modifications, whereas there was only HexNAc modification of T201, indicating that HexNAcHexNeuAc can be attached to T194 and assigned as mono-sialylated Galβ1-3GalNAc, an intermediate in *O*-glycan synthesis from *O*-GalNAc to di-sialylated Galβ1-3GalNAc. The HCD fragmentation pattern of each peptide with the same modification at different retention times showed that they were modified with glycans linked in different manners, α2,3 linked and α2,6 linked (Figure S11). The types of glycans for HexNAc at T201 were not determined to be *O*-GalNAc or *O*-GlcNAc from HCD spectra because the fragment ions of HexNAc at T201 were always detected with glycans at T194 and mixed with the fragment ions from glycans at T194. Notably, in contrast to the result of the digested peptides from the ABA-unbound fraction of rAAV6, no glycosylation was observed for the peptide containing T242. In short, the ABA-unbound fraction of rAAV6, which accounted for more than 99% of total particles, was modified only with *O*-GlcNAc at T242, whereas ABA-bound rAAV6 was modified with *O*-GalNAc or *O*-GalNAc derivatives at S156, T162, and T194 and with *O*-GlcNAc or *O*-GalNAc at T201.

Physicochemical and biological characterization of rAAV6 particles fractionated by ABA

The identified mucin-type *O*-glycans in the VP2 region may influence physicochemical and biological properties of rAAV6. To characterize the effect of rAAV6 glycosylation on particle distribution and VP ratio, band sedimentation analytical ultracentrifugation (BS-AUC) and capillary electrophoresis sodium dodecyl sulfate (CE-SDS) were performed for the large-scale fractionated rAAV6. BS-AUC can completely separate the *c*(*s*) distribution of full particles (FP) that encapsidate a full-length genome, empty particles (EP) without a

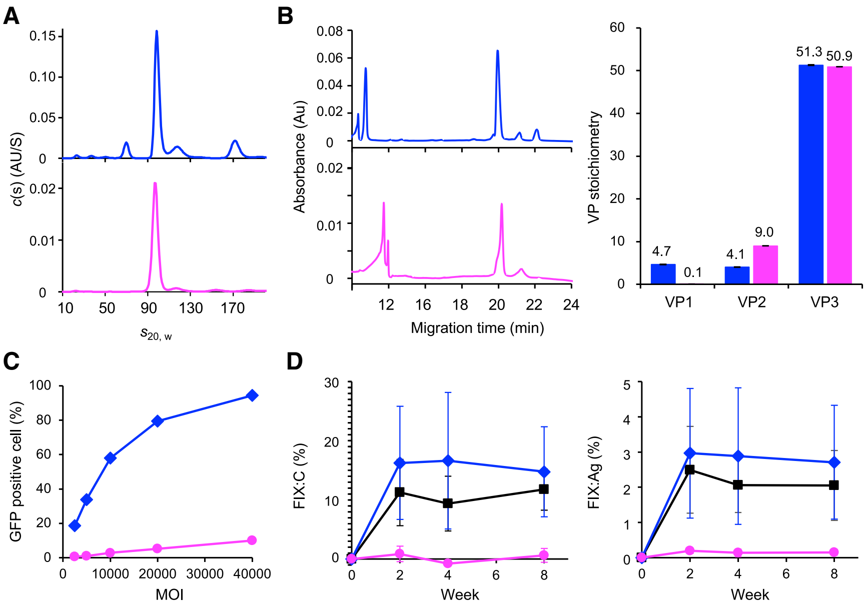

Figure 5. Physicochemical and biological characterizations of rAAV6 particles fractionated by ABA

(A) The *c*(s) distributions profiles of unbound and bound fractions of rAAV6. (B) Electropherograms of unbound and bound fractions of rAAV6 and VP ratios of each rAAV6. (C) The proportion of GFP-positive viable cells evaluated by flow cytometry at five MOIs (2.5 × 10^3, 5 × 10^3, 1 × 10^4, 2 × 10^4, and 4 × 10^4) in unbound and bound fractions of rAAV6. (D) FIX:C and FIX:Ag of mouse plasma measured 2, 4, 6, and 8 weeks after administration of pre-bound, unbound, and bound fractions of rAAV6. Each value was subtracted from the value at the time of administration. All plots show pre-fractions of rAAV6 in black, unbound fractions in blue, and bound fractions in magenta. Error bars show SD values caluclated from triplicate measurements (A, B, and C) or four mices (D).

genome, and partially or extra-filled particles and aggregates from a small amount of sample.[36] CE-SDS is a method to quantify the VP ratio from the peak area of separated VPs and their molar absorption coefficient.[5]

As shown in Figure 5A, two major peaks, at 98.1 ± 1.1 S and 69.2 ± 0.9 S, were observed in the *c*(s) distribution profile for the unbound fraction of rAAV6. Based on the amino acid and genomic sequences used in this study, we concluded that the 98.1 and 69.2 S peaks corresponded to FPs and EPs, respectively. Surprisingly, the peak corresponding to EPs disappeared in the bound fraction, indicating that only FPs were modified with mucin-type *O*-glycans interacting with ABA. CE-SDS analysis produced three major peaks, at 20, 21, and 22 min, for the unbound fraction of rAAV6 (Figure 5B). These peaks corresponded to VP3, VP2, and VP1, respectively, based on our previous study.[5] Only two major peaks, at 20 and 21 min, were observed for the bound fraction, meaning that mucin-type *O*-glycosylated rAAV6 particles incorporated a small amount of VP1 into the capsid. This was consistent with VP1 not being detected by western blotting, as shown in Figure 3A. The VP ratio calculated from the peak area in the electropherogram was VP1:VP2:VP3 = 4.66:4.05:51.28 (±0.07:0.09:0.10) for the unbound fraction of rAAV6 and VP1:VP2:VP3 = 0.08:9.02:50.90 (±0.00:0.03:0.03) for the bound fraction of rAAV6.

The VP1 unique region contains a phospholipase A2 domain, which is important for endosomal escape;[37,38] therefore, mucin-type *O*-glycosylated rAAV6 particles possibly have a low transduction efficiency because they lack VP1. To confirm this hypothesis, cultured cells were incubated at dose-ranging multiplicity of infections (MOIs) with rAAV6 recovered from post-ABA fractionation. Then, the number of transduced cells that express the transgene carried by rAAV6 was quantified by flow cytometry. The transduction efficiency of the ABA-unbound fraction was up to 94.4% ± 0.3% at MOI 4 × 10^4, whereas that of the ABA-bound fraction at the same MOI was only 10.0% ± 0.2% (Figure 5C). To further examine the transduction efficiency of the fractionated rAAV6 *in vivo*, we constructed and fractionated rAAV6 harboring the human coagulation factor IX (*hFIX*) gene with the Padua mutation (R338L) driven by the liver-specific HCRhAAT promoter. C57BL/6 mice were administered 4.5 × 10^9 vg/mouse of each rAAV6 fraction through the jugular vein, and then plasma hFIX activity (FIX:C) and antigen (FIX:Ag) were measured at the time of administration and 2, 4, and 8 weeks after administration, with 8 weeks as the endpoint. As expected from the *in vitro* transduction efficiency results, the levels of FIX:C and FIX:Ag were only slightly increased in the bound fractions after vector injection (Figure 5D). For the unbound fraction, the levels of FIX:C and FIX:Ag were higher, but not significantly, compared with that of pre-fraction rAAV6 because of the low amount of mucin-type *O*-glycosylated rAAV6 in the unbound fraction. Additionally, the number of rAAV6 genomes and *hFIX* mRNA levels within the liver 8 weeks after administration were significantly lower in mice treated with the bound fraction compared with the unbound fraction (Figure S12). The BS-AUC showed that the bound fraction contained only FP, indicating that the low transduction efficiency of the bound fraction was not caused by genome content but by the low abundance of VP1 in the ABA-bound particles. Unfortunately, the possibility that glycans directly influence transduction efficiency cannot be ruled out in this experiment because the premise is that AAVs lacking VP1 have no infectivity when co-infected with adenoviruses.[39] Therefore, mucin-type *O*-glycans attached to the VP2 region of rAAV6 may reduce the abundance of VP1 in the capsid, resulting in low transduction efficiency both *in vitro* and *in vivo.*

DISCUSSION

Glycosylation of AAV has been reported in previous studies;[6,9,14–18] however, there is little concrete evidence to show where in the AAV

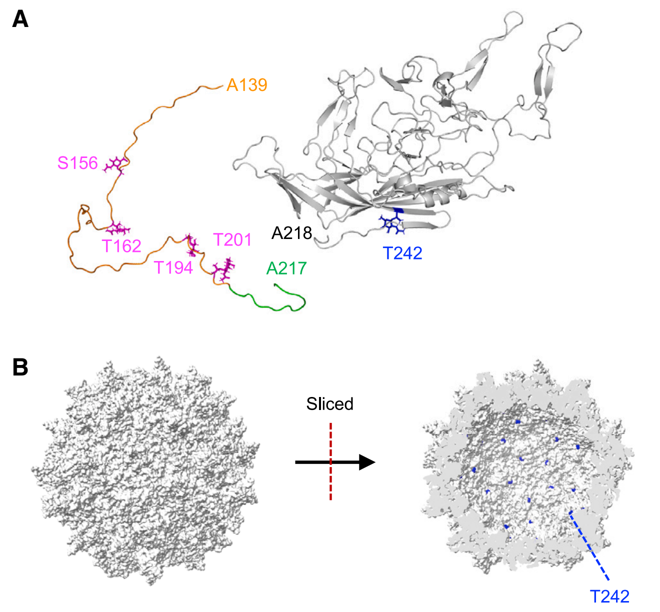

Figure 6. A structural model of rAAV6 glycosylation

(A) A structural model of the VP2 and VP3 region of rAAV6 modified with *O*-GlcNAc and *O*-GalNAc. The crystal structure of AAV6 VP3 (PDB: 3OAH) was used for A218–L736, whereas A139–A217 was derived from the VP2 structure, as modeled by AlphaFold2.[41] The glycans were attached by CHARMM-GUI Glycan Modeler.[42] *O*-GlcNAc is colored blue, *O*-GalNAc in magenta, the VP2 region in orange, the modeled VP3 in green, and VP3 from the crystal structure in gray. (B) A structural model of rAAV6 capsid based on the crystal structure of AAV6 VP3 (PDB: 3OAH). A whole capsid is shown on the left and a sliced-through capsid on the right. The residues colored blue are T242, which was identified as an *O*-GlcNAc attachment site.

capsid each type of glycan is attached, the exception being a study of AAV8 that produced glycopeptide mass spectra.[18]

Our comprehensive glycan profiling by lectin microarrays revealed that ABA can potentially recognize the *O*-glycans attached to rAAV6 capsids, whereas *N*-glycan-binding lectins can recognize the contaminants of HCPs. This indicates that glycosylation analysis of rAAV requires the use of highly purified samples to avoid the analysis of HCP glycosylation.

The mass spectrometry analysis of ABA-fractionated rAAV6 showed that *O*-GlcNAc at T242 in VP3 was not observed in the ABA-bound fraction but only in the ABA-unbound fraction, whereas the mucin-type *O*-glycosylation clusters, *O*-GalNAc derivatives with sialic acids, were only identified in VP2 in the ABA-bound fraction. These results indicate that *O*-GlcNAc in VP3 and mucin-type *O*-glycan modification in VP2 do not occur simultaneously; that is, if rAAV6 particles are modified with *O*-GlcNAc, then the particles do not undergo mucin-type *O*-glycan modification and vice versa. Thus, HexNAc at T201, the glycan types of which could not be determined by MS spectra, is assigned as *O*-GalNAc. Here, since the amount of peptide modified with *O*-GlcNAc in the rAAV6 pre-fraction was calculated to be approximately 0.05% of all modified and unmodified peptides, as shown in Figure S2, the binomial theorem showed that rAAV6 modified with *O*-GlcNAc accounts for about 3% of the rAAV6 particles without mucin-type *O*-glycan, which is 99.9% of the total particles, when VPs containing *O*-GlcNAc are uniformly contained in each particle.

Interestingly, the peptides modified with *O*-GalNAc or di-sialylated Galβ1-3GalNAc were identified in both ABA-bound and ABA-unbound peptides, which indicates that *O*-GalNAc and di-sialylated Galβ1-3GalNAc by themselves do not strongly bind to ABA (Figure S13). However, rAAV6 must have several combinations of mucin-type *O*-glycans because the capsid consists of 60 VPs, resulting in an avidity effect that enhances the interaction with ABA. Therefore, *O*-GlcNAc at T242 is not involved in the interaction of rAAV6 with ABA, whereas the combination of mucin-type *O*-glycans at S156, T162, T194, and T201 provide the main contribution to the binding of rAAV6 particles to ABA with polyvalent interactions.[40]

Despite MS searches including *N*-glycan modifications, *N*-glycans attached to unique rAAV6 peptides were not detected in any rAAV6 sample in this study. Considering that 0.05% of *O*-glycosylated peptides were successfully identified in the pre-fraction of rAAV6, even if rAAV6 was *N*-glycosylated, the *N*-glycosylated peptides should constitute less than 0.05% of the total peptides. We note that, if a high signal intensity for *N*-glycosylated peptides is observed for rAAV, then careful consideration is needed to determine whether the spectra are derived from contaminated HCPs. Therefore, we conclude that rAAV6 is not or minimally *N*-glycosylated. These results are consistent with a previous report that defined less than 1% as the upper limit for glycosylation of intact AAV2.[16]

The reported structure of rAAV6 places the *O*-GlcNAc at T242 of VP3 on the inside of the capsid (Figures 6A and 6B). The *O*-GlcNAc modification occurs within the nuclear and cytoplasmic compartments;[34] therefore, *O*-GlcNAc at T242 may be attached to VPs before the formation of rAAV6 particles. Although the structure of 1–217 in VP1, VP2, and the N-terminal region of VP3 has not been elucidated by X-ray crystallography or cryoelectron microscopy,[43] mucin-type *O*-glycan clusters at S156, T162, T194, and T201 in VP2 should be located at positions that are accessible by ABA. Accordingly, we propose the following hypothesis for a rAAV glycosylation scheme with biosynthesis of *O*-GalNAc glycans.[44] After VP synthesis, some VPs form particles without VP1. The particles then migrate to the nucleus for genome packaging and extrude a portion of VP2 on capsid formation or genome packaging. VP2 then undergoes *O*-GalNAc modification through the rough endoplasmic reticulum to the Golgi apparatus stage in an uncertain sequence. Alternatively, VP2 undergoes *O*-GalNAc modification at the rough endoplasmic reticulum stage before particle formation and then inhibits VP1 incorporation into the capsid. A portion of VP2 is then extruded before Golgi migration, and *O*-GalNAc attached to VP2 is further modified with sialic acids.

In this study, we revealed that rAAV6 is not or minimally *N*-glycosylated and slightly O-glycosylated. Our lectin-based fractionation

technique successfully enriched only mucin-type *O*-glycosylated rAAV6 particles and allowed us to precisely detect mucin-type O-glycan clusters attached to rAAV6 capsids despite the small number of glycosylated particles. The identified glycans did not contain the high-risk glycan structures, such as galactose-α1,3-galactose, which is responsible for immunogenicity;[45] therefore, the glycosylation of rAAV is not considered to be related to immunogenicity. Unfortunately, this study could not evaluate the direct influence of mucin-type *O*-glycans on transduction efficiency. The number of VP1 and VP2 incorporated into the capsid can alter the transduction efficiency;[46,47] therefore, further experiments using rAAVs that have the same VP ratios but with or without glycosylation are needed to answer this question. However, mucin-type *O*-glycosylated rAAV6 particles had a lower VP1 ratio, which resulted in lower transduction efficiency than that of rAAV6 without mucin-type *O*-glycans. In other words, mucin-type *O*-glycosylation of rAAV can indirectly influence transduction efficiency. Glycosylation is therefore considered to be a critical quality attribute for rAAV-based gene therapy drug products. However, if the amount of glycosylation is negligible, as in the case of rAAV6 produced by triple transfection of HEK293 or HEK293T cells, then it may have only a small effect on the overall transduction efficiency. This report details conclusive evidence that rAAV is *O*-glycosylated and provides in-depth insight into rAAV glycosylation that will impact rAAV-based therapeutics.

MATERIALS AND METHODS

Materials

rAAV6-CMV-EGFP was purchased from Sirion Biotech (Bavaria, Germany), SignaGen Laboratories (Frederick, MD, USA), Takara Bio (Shiga, Japan), and Vector Builder (Chicago, IL, USA). The encapsidated genome size and purification methods of each rAAV6 are shown in Table 1. To produce in-house rAAV6, a transgene plasmid (HCRhAAT–hFIX minigene), pAAV-Rep-Cap (serotype 6), and pAd helper were co-transfected into suspended Viral Production Cells (VPC) 2.0 cells (Thermo Fisher Scientific, Waltham, MA, USA) using the transfection reagent FectoVIR-AAV (Polyplus, Illkirch, France) and cultured in a flask. 4 days after transfection, the cells were harvested and lysed, and the lysate was filtered. rAAV6 particles were purified by affinity chromatography using AAVX prepacked columns (Thermo Fisher Scientific). To separate the FPs and EPs, the purified AAVs were processed by cesium chloride ultracentrifugation. FP fractions were collected and dialyzed in 1 $\times$ Phospho-buffered saline (PBS) buffer containing 200 mM NaCl and 0.001% poloxamer-188.

Lectin microarray

Differential glycan profiling of rAAV6 was performed by antibody overlay lectin microarray as described previously.[23] Briefly, purified rAAVs (2.5–40 $\times$ 10^9 vg) were diluted to 60 µL with TBSTx (1% Triton X-100 in TBS) and then applied to a LecChip v.1.0 (Precision System Science, Chiba, Japan), which included triplicate spots for each of the 45 lectins in each of the seven reaction wells. After incubation at 20°C for 12 h, 20 µg of human serum polyclonal IgG was added to the glass slide and incubated for 30 min. The reaction solution was discarded, and the glass slide was washed three times with TBSTx. Sixty microliters of biotinylated anti-AAV6 antibody (610159, Progen Biotechnik, Heidelberg, Germany) solution in TBSTx was applied to the array and incubated at 20°C for 1 h. After washing three times with TBSTx, 60 µL of Cy3-labeled streptavidin (Merck Millipore, Burlington, MA, USA) solution in TBSTx was added to the array and then incubated at 20°C for 30 min. The glass slide was rinsed with TBSTx and scanned by an evanescent-field fluorescence scanner, GlycoStationReader2300 (emukk, Mie, Japan). All data were analyzed with GlycoStationToolsPro v.3.0 (emukk). The net intensity value for each spot was calculated by subtracting the background value from the signal intensity values of three spots. Data obtained under suitable time exposure conditions with net intensities below 40,000 for all lectin spots were used to obtain glycan profiles. The signals of *Lycopersicon esculentum* lectin (LEL), *Solanum tuberosum* lectin (STL), and *Urtica dioica* lectin (UDA) had high background noise caused by undesirable binding to the detection antibody and were excluded from the lectin microarray analysis. Finally, the mean signal intensities with SD were calculated from triplicate spots and normalized against the mean values of 42 lectins immobilized on the array. PCA was performed for the mean-normalized signal intensities of each rAAV6 preparation using the covariance matrix model in R software (v.4.2.2).

Sample preparation for LC-MS/MS analysis with CID fragmentation

Glycopeptides were prepared using a method modified from previous studies.[48,49] rAAV6 was concentrated by acetone precipitation: 6 $\times$ 10^{11} vg (10 µg VPs) was added to a 4-fold volume of ice-cold acetone, incubated at −80°C for 1 h, centrifuged at 15,000 $\times$ *g* for 15 min at 4°C to precipitate the protein, and the supernatant removed. The precipitated protein was dissolved in 100 mM Tri-HCl (pH 9.0) containing 12 mM sodium deoxycholate and 12 mM sodium lauroylsarcosinate. The dissolved samples were reduced by incubation at 25°C for 0.5 h in the presence of 10 mM dithiothreitol and then alkylated by incubation with 20 mM iodoacetamide at 25°C in the dark for 0.5 h. The samples were 5-fold diluted by the addition of MilliQ water and digested with 1/200 weight Lys-C for 1 h at 37°C. The Lys-C-digested samples were further digested with 1/100 weight trypsin for 4 h at 37°C. Digestion was arrested by adding 0.5% trifluoroacetic acid and 66% ethyl acetate. Centrifugation was performed at 15,700 $\times$ *g* for 5 min at 25°C to remove the upper ethyl acetate layer containing detergents, and the remaining ethyl acetate was evaporated by vacuum drying for 0.5 h. The dried samples were dissolved in 0.1% formic acid water, filtered through a 0.22-µm filter, and analyzed by LC-MS/MS.

LC-MS/MS with CID fragmentation for rAAV6

LC-MS/MS of glycopeptides was performed using a method modified from a previous study.[5] Briefly, NanoElute Ultra High Performance Liquid Chromatography (CTC Analytics, Zwingen, Switzerland) coupled to a trapped ion mobility spectrometer with a time-of-flight instrument, timsTOF Pro (Bruker, Billerica, MA, USA), was used for

LC-MS/MS of glycopeptides. Water containing 0.1% (v/v) formic acid and acetonitrile containing 0.1% (v/v) formic acid were used as the mobile phase. Glycopeptides were separated on a C18 Aurora UHPLC column with CSI Fitting (AUR2-25075C18A-CSI, Ion Opticks, VIC, Australia) by applying an acetonitrile gradient from 0% to 30% in 30 min at a flow rate of 0.4 μL/min. Data analysis with ion mobility data was performed by Byos (ProteinMetrics, Cupertino, CA, USA).

Small-scale fractionation of glycosylated rAAV6

A 20-μL suspension of 1×10^{10} vg of rAAV6 in TBSTx was incubated overnight at 4°C with the equivalent volume of streptavidin beads pre-conjugated with 2 μg biotinylated ABA (Mitsubishi Gas Chemical Company, Tokyo, Japan). The supernatant was collected as the “unbound fraction,” and the beads were washed three times with 400 μL 1% poloxamer-188 in TBS. To remove nonspecifically bound proteins, 20 μL TBS containing 1% poloxamer-188 and 0.5 M glycine were added to the washed beads and incubated at 50°C for 10 min. After removing the supernatant, 20 μL elution buffer (Glycoprotein Eluting Solution for Galactose/GalNAc Binding Lectins, ES2100; Vector Laboratories, Newark, CA, USA) in TBS containing 1% poloxamer-188) was added to the beads and then incubated at 50°C for 10 min. The supernatant was collected as the “bound fraction.”

Large-scale fractionation of glycosylated rAAV6

A 100 mL suspension of 2.1×10^{14} vg of rAAV6 with TBSTx was reacted overnight at 4°C with 25 mL streptavidin beads pre-conjugated with 2.5 mg biotinylated ABA. The supernatant was collected and washed four times with 200 mL 1% poloxamer-188 in TBS. To remove nonspecific binding, 50 mL TBS containing 1% poloxamer-188 and 0.5 M glycine was added to the washed beads and incubated at 50°C for 10 min. For elution, 50 mL elution buffer was added to the beads and incubated at 50°C for 10 min 1×10^{13} vg was also fractionated for the LC-MS/MS analysis or 8×10^{13} vg for the animal experimentation following the method described above.

Western blot analysis

Each rAAV6 was denatured at 95°C for 5 min in sample buffer (60 mM Tris-HCl [pH 6.8], 2 [w/v] % SDS, 10 [w/v] % glycerol, and 0.005 [w/v] % BPB + 20 mM DTT). Denatured rAAV6s were electrophoresed with 25 mM Tris, 192 mM glycine, and 0.1% (w/v) SDS buffer under reducing conditions on 5%–20% polyacrylamide gels (Fujifilm Wako Pure Chemical, Osaka, Japan) and transferred to polyvinylidene fluoride (PVDF) membranes (Bio-Rad, Hercules, CA, USA). After treatment with Block Ace (DS Pharma Biomedical, Osaka, Japan), the membranes were incubated with 0.15 μg/mL of anti-M2BP polyclonal goat antibody (R&D Systems, Minneapolis, MN, USA) or 0.1 μg/mL of anti-AAV VP1/VP2/VP3 mouse monoclonal antibody (Progen Biotechnik) and then with anti-goat IgG-horseradish peroxidase (HRP) or anti-mouse IgG-HRP. Membranes were then incubated with ImmunoStar LD (Fujifilm Wako Pure Chemical). For sialidase treatment, each rAAV6 was incubated with sialidase A (ProZyme, CA, USA) at 37°C for 2 h.

ddPCR

The concentration of FPs was measured by ddPCR. Free nucleic acids in the sample solution were treated with DNase I (Takara Bio), followed by inactivation of DNase I by EDTA (Nacalai Tesque, Kyoto, Japan) (final concentration, 50 mM). The capsid was thermally denatured to release the internal nucleic acid. Each sample was then diluted in Tris-ethylenediaminetetraacetic acid (TE) buffer (pH 8.0) (Invitrogen, Carlsbad, CA, USA) containing 0.05% poloxamer-188. The sample and reaction mixture were mixed with ddPCR Supermix (Bio-Rad), forward and reverse primers (final concentration, 0.9 μM), and probes (final concentration, 0.25 μM). The following primers and probes were used: Inverted Terminal Repeat (ITR) forward primer, 5′-GGAACCCCTAGTGATGGAGTT-3′; ITR reverse primer, 5′-CGGCCTCAGTGAGCGA-3′; and ITR probe, 5′-[Fluorescein (FAM)]-CACTCCCTCTCTGCGCGCTCG-[Black Hole Quencher 1 (BHQ1)]-3′. Droplets were generated using a QX200 Droplet Generator (Bio-Rad) with the above mixture and droplet generator oil (Bio-Rad) in a DG8 cartridge (Bio-Rad). PCR reactions were conducted after sealing each well in the PCR plate (Eppendorf, Hamburg, Germany) using a PX1 PCR Plate Sealer (Bio-Rad) and a foil heat seal (Bio-Rad). The PCR program consisted of 95°C for 10 min for enzyme activation, followed by 40 cycles of 94°C for 30 s and 54°C for 30 s. After the reaction, the plate was transferred to a QX200 Droplet Reader (Bio-Rad) for scanning with positive or negative binary fluorescence. It was confirmed that the number of droplets used for measurement exceeded 10,000. The results (copies per well) obtained from QuantaSoft v.1.7 (Bio-Rad) were then converted to concentrations (vector genomes per milliliter).

LC-MS/MS with hybrid ETD/HCD fragmentation for fractionated rAAV6

The ABA-fractionated rAAV6 was digested on an S-trap column (Protifi, Huntington, NY, USA) using a workflow following the manufacturer’s instructions after ethanol precipitation. Briefly, the fractionated rAAV6 was reduced, alkylated, and acid denatured. All prepared samples were then diluted in binding/washing buffer and trapped on the column. After washing the column, 20 μL of 50 mM Tris-HCl containing Asp-N and Lys-C or Asp-N was added to the column and digested at 37°C for 16 h under humidified conditions. The resulting digested peptides were eluted with elution buffer, lyophilized, and redissolved in 20 μL pure water. The digested peptides from ABA-bound fraction were re-fractionated by ABA following the fractionation method described above. After the re-fractionation, ABA-unbound peptides were further enriched for glycopeptides with TSKgel Amide-80 (TOSOH, Tokyo, Japan) to avoid ion suppression by non-glycopeptides. The re-fractionated ABA-unbound and ABA-bound peptides were analyzed by LC-MS/MS with EThcD fragmentation, and the obtained spectral data were analyzed using Mascot (Matrix Science, Boston, MA, USA) and Byonic (ProteinMetrics, Cupertino, CA, USA).

The digested rAAV6 samples were analyzed by nanoLC-MS/MS on an Orbitrap Fusion Tribrid (Thermo Fisher Scientific) coupled to an UltiMate 3000 UHPLC system (Thermo Fisher Scientific) with a

trap column (Acclaim PepMap100 C18, 300 μm I.D. (inside diameter) × 5 mm, Thermo Fisher Scientific). The sample solutions were loaded onto a tip column (C18 column, 0.075 mm I.D. × 250 mm; 1.9-μm particles; Nikkyo Technos, Tokyo, Japan) with a linear gradient of 2.5%–36% acetonitrile in the presence of 0.1% formic acid at a flow rate of 300 nL/min. The peptides were measured using the triggered EThcD acquisition mode in the data-dependent mode. The trigger was set to an HCD fragment of $HexNAc+H^+$ (*m/z* 204.0872). Glycopeptides were identified based on the HCD and EThcD MS/MS spectra by database searches using Byonic (Protein Metrics). Then the *m/z* and elution time of the non-glycosylated forms of glycopeptides assigned by Byonic were given to GRable as input data, and the presence of glycopeptides with the same core peptide was predicted from the MS1 data. The AAV6 structures attached to identified glycan structures were modeled by CHARMM-GUI Glycan Modeler[42] and visualized with ChimeraX[50] and the PyMOL Molecular Graphics System v.2.0 (Schrödinger, New York, NY, USA).

BS-AUC for fractionated rAAV6

BS-AUC experiments and analyses were performed according to our previous study.[36] Briefly, 15 μL of the buffer or sample was loaded into a reference or sample reservoir well with a 12-mm band-forming centerpiece (Spin Analytical, South Berwick, ME, USA). The reference and sample sectors were loaded with 250 μL and 240 μL PBS/D_2O containing 0.001% poloxamer-188, respectively. Data were collected at 20°C using Optima AUC (Beckman Coulter, Brea, CA, USA) at 20,000 rpm with a UV detection system. The detection wavelength was set to 230 nm. Data were collected with a 10-μm radial increment every 150 s.

The collected sedimentation data were analyzed using the analytical zone centrifugation *c*(s) model implemented in the program SEDFIT (v.16.2b),[51] where the lamella width, frictional ratio, meniscus, time-invariant noise, and radial invariant noise were fitted, and a regularization level of 0.68 was used. The sedimentation coefficient ranges of 0–175 S were evaluated with a resolution of 350. The apparent sedimentation coefficient of each solute was converted to the sedimentation coefficient in water at 20°C, $s_{20,\,w}$. The figures of the *c*(s) distribution were generated using the program GUSSI (v.1.3.2).[52]

CE-SDS for fractionated rAAV6

The 7 × 10^{10} vg of the rAAV6 unbound fraction and 5 × 10^{10} vg of the rAAV6 bound fraction was mixed with 14.4 μL 10% SDS (Nippon Gene, Toyama, Japan) and 4.83 μL 2-mercaptoethanol (Nacalai Tesque), respectively. Each mixture was incubated at 70°C for 3 min, and the buffer was then exchanged twice with 70% matrix exchange solution (0.5 mL 2-mercaptoethanol and 9.5 mL 0.05% SDS diluted to 70% with MilliQ water) using an Amicon ultracentrifugal filter (Merck Millipore). The buffer-exchanged samples were incubated at 70°C for 3 min, and then 1 μL of 20-fold diluted 10-kD Internal Standard (SCIEX, Framingham, MA) in MilliQ water was added to make a total volume of 70 μL. A PA800 Plus Pharmaceutical Analysis CE system (SCIEX) equipped with a PDA (Photodiode Array) detector at 214 nm and 32 Karat software (v.10.3 Build 20, SCIEX) was used for all experiments. A bare fused-silica capillary (50 μm I.D., 30 cm total length, 20 cm effective length, SCIEX) was used for separation. Data acquisition and analysis were performed using 32 Karat software (v.10.3 Build 20, SCIEX).

In vitro transduction assay

HeLaRC32 cells were seeded at 5 × 10^4 cells/well in 24-well plates in 0.5 mL of 10% fetal bovine serum (FBS) (HyClone, Marlborough, MA, USA) containing DMEM (Sigma-Aldrich, Burlington, MA, USA). Cells were infected with AAV vectors at five MOI (2.5 × 10^3, 5 × 10^3, 1 × 10^4, 2 × 10^4, and 4 × 10^4) in triplicate. Cells were incubated at 37°C for 2 days and then harvested. The percentage of viable cells expressing EGFP was assayed using the CytoFLEX flow cytometry system (Beckman Coulter).

Animal experimentation

All animal experiment procedures were approved by The Institutional Animal Care and Concern Committee of Jichi Medical University (permission numbers 19029-07, 20023-01, 20054-02, and 20051-06). Animals were cared for according to the committee's guidelines and ARRIVE guidelines.[53,54]

C57BL/6J mice were purchased from Japan SLC (Shizuoka, Japan) and maintained in isolators in the specific pathogen-free facility of Jichi Medical University at 23°C ± 3°C with a 12:12 h light/dark cycle. A 100 μL of 4.5 × 10^9 vg/mouse of rAAV6 was administered intravenously through the jugular vein to four mice under isoflurane (1%–3%) anesthesia. Mice were anesthetized with isoflurane (1%–3%) to obtain plasma samples. Blood samples were drawn from the jugular vein of mice with a 29G micro-syringe (Terumo, Tokyo, Japan) pre-filled with 3.8% sodium citrate solution (Harasawa Pharmaceutical, Tokyo, Japan). The volume of blood samples was 1/10 the volume of the sodium citrate solution. Blood samples were taken at the time of rAAV6 administration and 2, 4, and 8 weeks after administration, with 8 weeks being the endpoint. Platelet-poor plasma was isolated by centrifugation at 2,500 × *g* for 10 min and then frozen and stored at −80°C until analysis. Eight weeks after treatment, mice were euthanized and perfused with PBS, and livers were harvested for quantification of rAAV6 genomes in liver.

Measurement of FIX:C and FIX:Ag

FIX:C was measured using Revohem FIX Chromogenic, a blood coagulation FIX measurement kit (Sysmex, Kobe, Japan) with an automated coagulation analyzer (Sysmex CS-1600 analyzer, Sysmex). FIX:Ag in plasma was measured as described previously.[55]

Quantification of AAV vector genome copy number and *hFIX* mRNA in liver

Genomic DNA was extracted from liver tissue using a nucleic acid extraction system (GENE PREP STAR 480; Kurabo, Osaka, Japan). AAV vector genome copy number was quantified by qPCR using Thunderbird Probe qPCR Mix (Toyobo, Osaka, Japan) and the

QuantStudio 12K Flex real-time PCR system (Thermo Fisher Scientific), as described previously.[56,57] AAV vector genome in liver DNA was estimated using a standard of linearized plasmid containing the target sequence and is expressed as copy number per mouse diploid genome (6 pg/cell).[58,59] The following primers and probes were used: hAATp_Primer_F, 5′-TTCGGTAAGTGCAGTGGAAG-3′; hAATp_Probe, 5′-ACTCAGATCCCAGCCAGTGGACTTA-3′; hAATp_Primer_R, 5′-CAGTTATCGGAGGAGCAAACA-3′.

RNA in liver was extracted with an RNeasy Mini Kit (QIAGEN). The RNA samples were reverse-transcribed using a PrimeScript RT Reagent Kit (Takara Bio). qPCR was performed with Thunderbird SYBR qPCR Mix (Toyobo) in the QuantStudio 12K Flex real-time PCR system (Thermo Fisher Scientific). The levels of *hFIX* mRNA were normalized to those of *Hprt1*. The RQ was automatically calculated in QuantStudio 12K Flex (Thermo Fisher Scientific). The following primers were used: hFIX_F, 5′-GTTCCATGAAGGTGGTAGAGA-3′; hFIX_R, 5′-GCTGATAATCCCAGTCAGGAAG-3′; mHPRT1_F, 5′-GTTGGATACAGGCCAGACTTTGTTG-3′; and mHPRT1_R, 5′-GATTCAACTYGCGCTCATCTTAGG-3′.

DATA AND CODE AVAILABILITY

The data of this study are available from the corresponding author upon reasonable request.

SUPPLEMENTAL INFORMATION

Supplemental information can be found online at https://doi.org/10.1016/j.omtm.2024.101256.

ACKNOWLEDGMENTS

This study was supported by a grant-in-aid from “Research and development of core technologies for gene and cell therapy” supported by the Japan Agency for Medical Research and Development (AMED) (grants JP21ae0201001 and JP21ae0201002). We thank Prof. Takashi Okada for the preliminary discussion on this study. We thank Sayuki Iijima, Kaori Yamauchi, and Hiroko Shimazaki (GlycoTechnica Ltd., Yokohama, Japan) for support with the lectin microarray analyses and Tamaki Aoki, Mika Kishimoto, and Hiromi Ozaki (Jichi Medical University, Tochigi, Japan) for help with the measurement of *in vivo* transduction efficiency. The image of the mouse and syringe in graphical abstract was created with BioRender. We thank Jeremy Allen, PhD, from Edanz (https://jp.edanz.com/ac) for editing a draft of this manuscript.

AUTHOR CONTRIBUTIONS

Conceptualization, A.K. and S.U.; investigation, K.I., S.K., H.S., T.M., M.F., R.S., K.B., C.M.-K., A.T., S.M., H.K., and Y.K.; resources, M.F., Y.T., and A.M.; visualization, Y.Y.; writing – original draft, Y.Y. and S.U.; writing – review & editing, Y.Y., T.T., T.O., A.K., and S.U.

DECLARATION OF INTERESTS

S.K. and C.M.-K.’s have a relationship with GlycoTechnica Ltd. that includes employment; S.K. has a relationship with Precision System Science Co. Ltd.; T.M. and M.F. have relationships with U-Medico Inc. that include employment; S.U. has a relationship with U-Medico Inc. that includes founder and CSO.

REFERENCES

1. Zaiss, A.-K., Liu, Q., Bowen, G.P., Wong, N.C.W., Bartlett, J.S., and Muruve, D.A. (2002). Differential activation of innate immune responses by adenovirus and adeno-associated virus vectors. J. Virol. *76*, 4580–4590.
2. Asokan, A., Schaffer, D.V., and Samulski, R.J. (2012). The AAV vector toolkit: poised at the clinical crossroads. Mol. Ther. *20*, 699–708.
3. Calcedo, R., and Wilson, J.M. (2013). Humoral Immune Response to AAV. Front. Immunol. *4*, 341.
4. Naso, M.F., Tomkowicz, B., Perry, W.L., 3rd, and Strohl, W.R. (2017). Adeno-Associated Virus (AAV) as a Vector for Gene Therapy. BioDrugs *31*, 317–334.
5. Oyama, H., Ishii, K., Maruno, T., Torisu, T., and Uchiyama, S. (2021). Characterization of Adeno-Associated Virus Capsid Proteins with Two Types of VP3-Related Components by Capillary Gel Electrophoresis and Mass Spectrometry. Hum. Gene Ther. *32*, 1403–1416.
6. Mary, B., Maurya, S., Arumugam, S., Kumar, V., and Jayandharan, G.R. (2019). Post-translational modifications in capsid proteins of recombinant adeno-associated virus (AAV) 1-rh10 serotypes. FEBS J. *286*, 4964–4981.
7. Liu, A.P., Patel, S.K., Xing, T., Yan, Y., Wang, S., and Li, N. (2020). Characterization of Adeno-Associated Virus Capsid Proteins Using Hydrophilic Interaction Chromatography Coupled with Mass Spectrometry. J. Pharm. Biomed. Anal. *189*, 113481.
8. Zhou, Y., and Wang, Y. (2023). Direct deamidation analysis of intact adeno-associated virus serotype 9 capsid proteins using reversed-phase liquid chromatography. Anal. Biochem. *668*, 115099.
9. Liu, S., Li, J., Peraramelli, S., Luo, N., Chen, A., Dai, M., Liu, F., Yu, Y., Leib, R.D., Li, Y., et al. (2024). Systematic comparison of rAAV vectors manufactured using large-scale suspension cultures of Sf9 and HEK293 cells. Mol. Ther. *32*, 74–83.
10. Frederick, A., Sullivan, J., Liu, L., Adamowicz, M., Lukason, M., Raymer, J., Luo, Z., Jin, X., Rao, K.N., and O’Riordan, C. (2020). Engineered Capsids for Efficient Gene Delivery to the Retina and Cornea. Hum. Gene Ther. *31*, 756–774.
11. Zhong, L., Li, B., Jayandharan, G., Mah, C.S., Govindasamy, L., Agbandje-McKenna, M., Herzog, R.W., Weigel-Van Aken, K.A., Hobbs, J.A., Zolotukhin, S., et al. (2008). Tyrosine-phosphorylation of AAV2 vectors and its consequences on viral intracellular trafficking and transgene expression. Virology *381*, 194–202.
12. Giles, A.R., Sims, J.J., Turner, K.B., Govindasamy, L., Alvira, M.R., Lock, M., and Wilson, J.M. (2018). Deamidation of Amino Acids on the Surface of Adeno-Associated Virus Capsids Leads to Charge Heterogeneity and Altered Vector Function. Mol. Ther. *26*, 2848–2862.
13. Bing, S.J., Justesen, S., Wu, W.W., Sajib, A.M., Warrington, S., Baer, A., Thorgrimsen, S., Shen, R.-F., and Mazor, R. (2022). Differential T cell immune responses to deamidated adeno-associated virus vector. Mol. Ther. Methods Clin. Dev. *24*, 255–267.
14. Xie, Y., and Butler, M. (2024). N-glycomic profiling of capsid proteins from adeno-associated virus serotypes. Glycobiology *34*, cwad074. https://doi.org/10.1093/glycob/cwad074.
15. Mary, B., Maurya, S., Kumar, M., Bammidi, S., Kumar, V., and Jayandharan, G.R. (2019). Molecular Engineering of Adeno-Associated Virus Capsid Improves Its Therapeutic Gene Transfer in Murine Models of Hemophilia and Retinal Degeneration. Mol. Pharm. *16*, 4738–4750.
16. Murray, S., Nilsson, C.L., Hare, J.T., Emmett, M.R., Korostelev, A., Ongley, H., Marshall, A.G., and Chapman, M.S. (2006). Characterization of the capsid protein glycosylation of adeno-associated virus type 2 by high-resolution mass spectrometry. J. Virol. *80*, 6171–6176.
17. Aloor, A., Zhang, J., Gashash, E.A., Parameswaran, A., Chrzanowski, M., Ma, C., Diao, Y., Wang, P.G., and Xiao, W. (2018). Site-Specific N-Glycosylation on the AAV8 Capsid Protein. Viruses *10*, 644. https://doi.org/10.3390/v10110644.
18. Rumachik, N.G., Malaker, S.A., Poweleit, N., Maynard, L.H., Adams, C.M., Leib, R.D., Cirolia, G., Thomas, D., Stamnes, S., Holt, K., et al. (2020). Methods Matter: Standard Production Platforms for Recombinant AAV Produce Chemically and Functionally Distinct Vectors. Mol. Ther. Methods Clin. Dev. *18*, 98–118.

19. Chung, C.H., Mirakhur, B., Chan, E., Le, Q.-T., Berlin, J., Morse, M., Murphy, B.A., Satinover, S.M., Hosen, J., Mauro, D., et al. (2008). Cetuximab-induced anaphylaxis and IgE specific for galactose-alpha-1,3-galactose. N. Engl. J. Med. *358*, 1109–1117.

20. Ghaderi, D., Taylor, R.E., Padler-Karavani, V., Diaz, S., and Varki, A. (2010). Implications of the presence of N-glycolylneuraminic acid in recombinant therapeutic glycoproteins. Nat. Biotechnol. *28*, 863–867.

21. Mechref, Y. (2012). Use of CID/ETD mass spectrometry to analyze glycopeptides. Curr. Protoc. Protein Sci. *Chapter 12*, 12.11.1–12.11.11.

22. Wuhrer, M., Catalina, M.I., Deelder, A.M., and Hokke, C.H. (2007). Glycoproteomics based on tandem mass spectrometry of glycopeptides. J. Chromatogr., B: Anal. Technol. Biomed. Life Sci. *849*, 115–128.

23. Kuno, A., Kato, Y., Matsuda, A., Kaneko, M.K., Ito, H., Amano, K., Chiba, Y., Narimatsu, H., and Hirabayashi, J. (2009). Focused differential glycan analysis with the platform antibody-assisted lectin profiling for glycan-related biomarker verification. Mol. Cell. Proteomics *8*, 99–108.

24. Hiono, T., and Kuno, A. (2022). Glycan Profiling of Viral Glycoproteins with the Lectin Microarray. Methods Mol. Biol. *2556*, 59–68.

25. Bojar, D., Meche, L., Meng, G., Eng, W., Smith, D.F., Cummings, R.D., and Mahal, L.K. (2022). A Useful Guide to Lectin Binding: Machine-Learning Directed Annotation of 57 Unique Lectin Specificities. ACS Chem. Biol. *17*, 2993–3012.

26. Bro, R., and Smilde, A.K. (2014). Principal component analysis. Anal. Methods *6*, 2812–2831.

27. Van Damme, E.J., Briké, F., Winter, H.C., Van Leuven, F., Goldstein, I.J., and Peumans, W.J. (1996). Molecular cloning of two different mannose-binding lectins from tulip bulbs. Eur. J. Biochem. *236*, 419–427.

28. Yagi, F., Miyamoto, M., Abe, T., Minami, Y., Tadera, K., and Goldstein, I.J. (1997). Purification and carbohydrate-binding specificity of Agrocybe cylindracea lectin. Glycoconj. J. *14*, 281–288.

29. Nakamura-Tsuruta, S., Kominami, J., Kuno, A., and Hirabayashi, J. (2006). Evidence that Agaricus bisporus agglutinin (ABA) has dual sugar-binding specificity. Biochem. Biophys. Res. Commun. *347*, 215–220.

30. Denard, J., Beley, C., Kotin, R., Lai-Kuen, R., Blot, S., Leh, H., Asokan, A., Samulski, R.J., Moullier, P., Voit, T., et al. (2012). Human galectin 3 binding protein interacts with recombinant adeno-associated virus type 6. J. Virol. *86*, 6620–6631.

31. Kuno, A., Ikehara, Y., Tanaka, Y., Ito, K., Matsuda, A., Sekiya, S., Hige, S., Sakamoto, M., Kage, M., Mizokami, M., and Narimatsu, H. (2013). A serum "sweet-doughnut" protein facilitates fibrosis evaluation and therapy assessment in patients with viral hepatitis. Sci. Rep. *3*, 1065.

32. Noro, E., Matsuda, A., Kyoutou, T., Sato, T., Tomioka, A., Nagai, M., Sogabe, M., Tsuruno, C., Takahama, Y., Kuno, A., et al. (2021). N-glycan structures of Wisteria floribunda agglutinin-positive Mac2 binding protein in the serum of patients with liver fibrosis. Glycobiology *31*, 1268–1278.

33. Rumachik, N.G., Malaker, S.A., and Paulk, N.K. (2021). VectorMOD: Method for Bottom-Up Proteomic Characterization of rAAV Capsid Post-Translational Modifications and Vector Impurities. Front. Immunol. *12*, 657795.

34. Zachara, N.E., Akimoto, Y., Boyce, M., and Hart, G.W. (2022). The O-GlcNAc Modification. In Essentials of Glycobiology, 4th ed., *Chapter 19*, A. Varki, R.D. Cummings, J.D. Esko, P. Stanley, G.W. Hart, M. Aebi, D. Mohnen, T. Kinoshita, N.H. Packer, and J.H. Prestegard, et al., eds. (Cold Spring Harbor Laboratory Press).

35. Nagai-Okatani, C., Tominaga, D., Tomioka, A., Sakaue, H., Goda, N., Ko, S., Kuno, A., and Kaji, H. (2023). GRable version 1.0: A software tool for site-specific glycoform analysis using the improved Glyco-RIDGE method with parallel clustering and MS2 information. Preprint at bioRxiv. https://doi.org/10.1101/2023.10.30.564073.

36. Maruno, T., Ishii, K., Torisu, T., and Uchiyama, S. (2023). Size Distribution Analysis of the Adeno-Associated Virus Vector by the c(s) Analysis of Band Sedimentation Analytical Ultracentrifugation with Multiwavelength Detection. J. Pharmaceut. Sci. *112*, 937–946.

37. Girod, A., Wobus, C.E., Zádori, Z., Ried, M., Leike, K., Tijssen, P., Kleinschmidt, J.A., and Hallek, M. (2002). The VP1 capsid protein of adeno-associated virus type 2 is carrying a phospholipase A2 domain required for virus infectivity. J. Gen. Virol. *83*, 973–978.

38. Stahnke, S., Lux, K., Uhrig, S., Kreppel, F., Hösel, M., Coutelle, O., Ogris, M., Hallek, M., and Büning, H. (2011). Intrinsic phospholipase A2 activity of adeno-associated virus is involved in endosomal escape of incoming particles. Virology *409*, 77–83.

39. Grieger, J.C., Snowdy, S., and Samulski, R.J. (2006). Separate basic region motifs within the adeno-associated virus capsid proteins are essential for infectivity and assembly. J. Virol. *80*, 5199–5210.

40. Wu, A.M., Liu, J.-H., Gong, Y.-P., Li, C.-C., and Chang, E.-T. (2010). Multiple recognition systems adopting four different glycotopes at the same domain for the Agaricus bisporus agglutinin-glycan interactions. FEBS Lett. *584*, 3561–3566.

41. Mirdita, M., Schütze, K., Moriwaki, Y., Heo, L., Ovchinnikov, S., and Steinegger, M. (2022). ColabFold: making protein folding accessible to all. Nat. Methods *19*, 679–682.

42. Park, S.-J., Lee, J., Qi, Y., Kern, N.R., Lee, H.S., Jo, S., Joung, I., Joo, K., Lee, J., and Im, W. (2019). CHARMM-GUI Glycan Modeler for modeling and simulation of carbohydrates and glycoconjugates. Glycobiology *29*, 320–331.

43. Stagg, S.M., Yoshioka, C., Davulcu, O., and Chapman, M.S. (2022). Cryo-electron Microscopy of Adeno-associated Virus. Chem. Rev. *122*, 14018–14054.

44. Brockhausen, I., Wandall, H.H., Hagen, K.G.T., and Stanley, P. (2009). O-GalNAc Glycans. In Essentials of Glycobiology, 4th ed., A. Varki, R.D. Cummings, J.D. Esko, P. Stanley, G.W. Hart, M. Aebi, D. Mohnen, T. Kinoshita, N.H. Packer, and J.H. Prestegard, et al., eds. (Cold Spring Harbor Laboratory Press).

45. Kuriakose, A., Chirmule, N., and Nair, P. (2016). Immunogenicity of Biotherapeutics: Causes and Association with Posttranslational Modifications. J. Immunol. Res. *2016*, 1298473. https://doi.org/10.1155/2016/1298473.

46. Bosma, B., du Plessis, F., Ehlert, E., Nijmeijer, B., de Haan, M., Petry, H., and Lubelski, J. (2018). Optimization of viral protein ratios for production of rAAV serotype 5 in the baculovirus system. Gene Ther. *25*, 415–424.

47. Onishi, T., Nonaka, M., Maruno, T., Yamaguchi, Y., Fukuhara, M., Torisu, T., Maeda, M., Abbatiello, S., Haris, A., Richardson, K., et al. (2023). Enhancement of recombinant adeno-associated virus activity by improved stoichiometry and homogeneity of capsid protein assembly. Mol. Ther. Methods Clin. Dev. *31*, 101142.

48. Masuda, T., Tomita, M., and Ishihama, Y. (2008). Phase transfer surfactant-aided trypsin digestion for membrane proteome analysis. J. Proteome Res. *7*, 731–740.

49. Togayachi, A., Tomioka, A., Fujita, M., Sukegawa, M., Noro, E., Takakura, D., Miyazaki, M., Shikanai, T., Narimatsu, H., and Kaji, H. (2018). Identification of Poly-N-Acetyllactosamine-Carrying Glycoproteins from HL-60 Human Promyelocytic Leukemia Cells Using a Site-Specific Glycome Analysis Method, Glyco-RIDGE. J. Am. Soc. Mass Spectrom. *29*, 1138–1152.

50. Pettersen, E.F., Goddard, T.D., Huang, C.C., Meng, E.C., Couch, G.S., Croll, T.I., Morris, J.H., and Ferrin, T.E. (2021). UCSF ChimeraX: Structure visualization for researchers, educators, and developers. Protein Sci. *30*, 70–82.

51. Schuck, P. (1998). Sedimentation Analysis of Noninteracting and Self-Associating Solutes Using Numerical Solutions to the Lamm Equation. Biophys. J. *75*, 1503–1512.

52. Brautigam, C.A. (2015). Calculations and Publication-Quality Illustrations for Analytical Ultracentrifugation Data. Methods Enzymol. *562*, 109–133.

53. Kilkenny, C., Browne, W.J., Cuthill, I.C., Emerson, M., and Altman, D.G. (2010). Improving bioscience research reporting: the ARRIVE guidelines for reporting animal research. PLoS Biol. *8*, e1000412.

54. Percie du Sert, N., Hurst, V., Ahluwalia, A., Alam, S., Avey, M.T., Baker, M., Browne, W.J., Clark, A., Cuthill, I.C., Dirnagl, U., et al. (2020). The ARRIVE guidelines 2.0: Updated guidelines for reporting animal research. PLoS Biol. *18*, e3000410.

55. Hiramoto, T., Kashiwakura, Y., Hayakawa, M., Baatartsogt, N., Kamoshita, N., Abe, T., Inaba, H., Nishimasu, H., Uosaki, H., Hanazono, Y., et al. (2023). PAM-flexible Cas9-mediated base editing of a hemophilia B mutation in induced pluripotent stem cells. Commun. Med. *3*, 56.

56. Kashiwakura, Y., Endo, K., Ugajin, A., Kikuchi, T., Hishikawa, S., Nakamura, H., Katakai, Y., Baatartsogt, N., Hiramoto, T., Hayakawa, M., et al. (2023). Efficient gene transduction in pigs and macaques with the engineered AAV vector AAV.GT5 for hemophilia B gene therapy. Mol. Ther. Methods Clin. Dev. *30*, 502–514.

57. Baatartsogt, N., Kashiwakura, Y., Hiramoto, T., Hayakawa, M., Kamoshita, N., and Ohmori, T. (2023). Successful liver transduction by re-administration of different adeno-associated virus vector serotypes in mice. J. Gene Med. *25*, e3505.

58. Capparelli, R., Cottone, C., D'Apice, L., Viscardi, M., Colantonio, L., Lucretti, S., and Iannelli, D. (1997). DNA content differences in laboratory mouse strains determined by flow cytometry. Cytometry *29*, 261–266.

59. Kashiwakura, Y., and Ohmori, T. (2023). Genome Editing of Murine Liver Hepatocytes by AAV Vector-Mediated Expression of Cas9 *In Vivo*. Methods Mol. Biol. *2637*, 195–211.

OMTM, Volume *32*

Supplemental information

Glycosylation of recombinant adeno-associated virus serotype 6

Yuki Yamaguchi, Kentaro Ishii, Sachiko Koizumi, Hiroaki Sakaue, Takahiro Maruno, Mitsuko Fukuhara, Risa Shibuya, Yasuo Tsunaka, Aoba Matsushita, Karin Bandoh, Tetsuo Torisu, Chie Murata-Kishimoto, Azusa Tomioka, Saho Mizukado, Hiroyuki Kaji, Yuji Kashiwakura, Tsukasa Ohmori, Atsushi Kuno, and Susumu Uchiyama

Table S1. Sample information of the lectin microarrays used for the PCA analysis shown in Figure 1B. The number and color correspond to the dot in PCA score plot.

The dot number	Sample	Amount (vg)
1	Sample 1	2.5×10^{9}
2	Sample 1	1×10^{10}
3	Sample 1	4×10^{10}
4	Sample 2	2.5×10^{9}
5	Sample 2	1×10^{10}
6	Sample 2	4×10^{10}
7	Sample 3	2.5×10^{9}
8	Sample 3	1×10^{10}
9	Sample 3	4×10^{10}
10	Sample 3	2.5×10^{9}
11	Sample 4	2.5×10^{9}
12	Sample 4	1×10^{10}
13	Sample 4	4×10^{10}
14	Sample 5	2.5×10^{9}
15	Sample 5	1×10^{10}
16	Sample 5	4×10^{10}
17	Sample 6	2.5×10^{9}
18	Sample 6	5×10^{9}

Table S2. The list of identified proteins contaminating Sample 4.

Several bovine serum proteins (colored blue) and human galectin 3-binding protein (colored red) were identified in Sample 4.

Accession	-10lgP	Coverage (%)	Area	#Peptides	Avg. Mass	Description
VP1_AAV6	215.39	74	3.81E+08	47	81411	VP1_AAV6
#CONTAM#Q7SIH1|A2MG_BOVIN	241.3	66	3.35E+08	77	167575	Alpha-2-macroglobulin OS=Bos taurus OX=9913 GN=A2M PE=1 SV=2
#CONTAM#A7E3W2|LG3BP_BOVIN	174.09	40	6.08E+07	18	62127	Galectin-3-binding protein OS=Bos taurus OX=9913 GN=LGALS3BP PE=1 SV=1
#CONTAM#Q3Y5Z3|ADIPO_BOVIN	115.09	32	2.42E+07	6	26133	Adiponectin OS=Bos taurus OX=9913 GN=ADIPOQ PE=1 SV=1
#CONTAM#P06748|NPM_HUMAN	147.68	46	2.23E+07	10	32575	Nucleophosmin OS=Homo sapiens OX=9606 GN=NPM1 PE=1 SV=2
#CONTAM#O46415|FRIL_BOVIN	129.49	63	2.01E+07	10	19988	Ferritin light chain OS=Bos taurus OX=9913 GN=FTL PE=2 SV=3
#CONTAM#P02792|FRIL_HUMAN	122.99	48	1.52E+07	9	20020	Ferritin light chain OS=Homo sapiens OX=9606 GN=FTL PE=1 SV=2
#CONTAM#P55072|TERA_HUMAN	199	68	8.51E+06	47	89322	Transitional endoplasmic reticulum ATPase OS=Homo sapiens OX=9606 GN=VCP PE=1 SV=4
#CONTAM#Q01853|TERA_MOUSE	199	68	8.51E+06	47	89322	Transitional endoplasmic reticulum ATPase OS=Mus musculus OX=10090 GN=Vcp PE=1 SV=4
#CONTAM#P46462|TERA_RAT	199	68	8.51E+06	47	89349	Transitional endoplasmic reticulum ATPase OS=Rattus norvegicus OX=10116 GN=Vcp PE=1 SV=3
#CONTAM#P00761|TRYP_PIG	93.86	34	6.47E+06	5	24409	Trypsin OS=Sus scrofa OX=9823 PE=1 SV=1
#CONTAM#P04406|G3P_HUMAN	153.88	58	5.76E+06	17	36053	Glyceraldehyde-3-phosphate dehydrogenase OS=Homo sapiens OX=9606 GN=GAPDH PE=1 SV=3

#CONTAM#Q5R8J7|FRIH_PONAB	138.85	72	5.65E+06	13	21226	Ferritin heavy chain OS=Pongo abelii OX=9601 GN=FTH1 PE=2 SV=3
#CONTAM#P02794|FRIH_HUMAN	138.85	72	5.65E+06	13	21226	Ferritin heavy chain OS=Homo sapiens OX=9606 GN=FTH1 PE=1 SV=2
#CONTAM#P14618|KPYM_HUMAN	168.02	53	5.50E+06	22	57937	Pyruvate kinase PKM OS=Homo sapiens OX=9606 GN=PKM PE=1 SV=4
#CONTAM#Q01105|SET_HUMAN	117.82	31	5.11E+06	9	33489	Protein SET OS=Homo sapiens OX=9606 GN=SET PE=1 SV=3
#CONTAM#Q9BXJ4|C1QT3_HUMAN	100.58	21	4.95E+06	5	26994	Complement C1q tumor necrosis factor-related protein 3 OS=Homo sapiens OX=9606 GN=C1QTNF3 PE=1 SV=1
#CONTAM#Q9BQA1|MEP50_HUMAN	145.04	43	4.67E+06	11	36724	Methylosome protein 50 OS=Homo sapiens OX=9606 GN=WDR77 PE=1 SV=1
#CONTAM#Q07021|C1QBP_HUMAN	118.24	41	4.42E+06	7	31362	Complement component 1 Q subcomponent-binding protein mitochondrial OS=Homo sapiens OX=9606 GN=C1QBP PE=1 SV=1
#CONTAM#P04075|ALDOA_HUMAN	163.86	73	4.41E+06	22	39420	Fructose-bisphosphate aldolase A OS=Homo sapiens OX=9606 GN=ALDOA PE=1 SV=2
#CONTAM#P02769|ALBU_BOVIN	178.52	58	3.64E+06	32	69294	Albumin OS=Bos taurus OX=9913 GN=ALB PE=1 SV=4
#CONTAM#P15636|API_ACHLY	132.47	16	3.33E+06	9	68125	Protease 1 OS=Achromobacter lyticus OX=224 PE=1 SV=1
#CONTAM#Q08380|LG3BP_HUMAN	139.96	29	2.95E+06	14	65331	Galectin-3-binding protein OS=Homo sapiens OX=9606 GN=LGALS3BP PE=1 SV=1
#CONTAM#P30048|PRDX3_HUMAN	108.06	31	2.82E+06	7	27693	Thioredoxin-dependent peroxide reductase mitochondrial OS=Homo sapiens OX=9606 GN=PRDX3 PE=1 SV=3
#CONTAM#Q5REY3|PRDX3_PONAB	108.06	31	2.82E+06	7	27700	Thioredoxin-dependent peroxide reductase mitochondrial OS=Pongo abelii OX=9601 GN=PRDX3 PE=2 SV=1

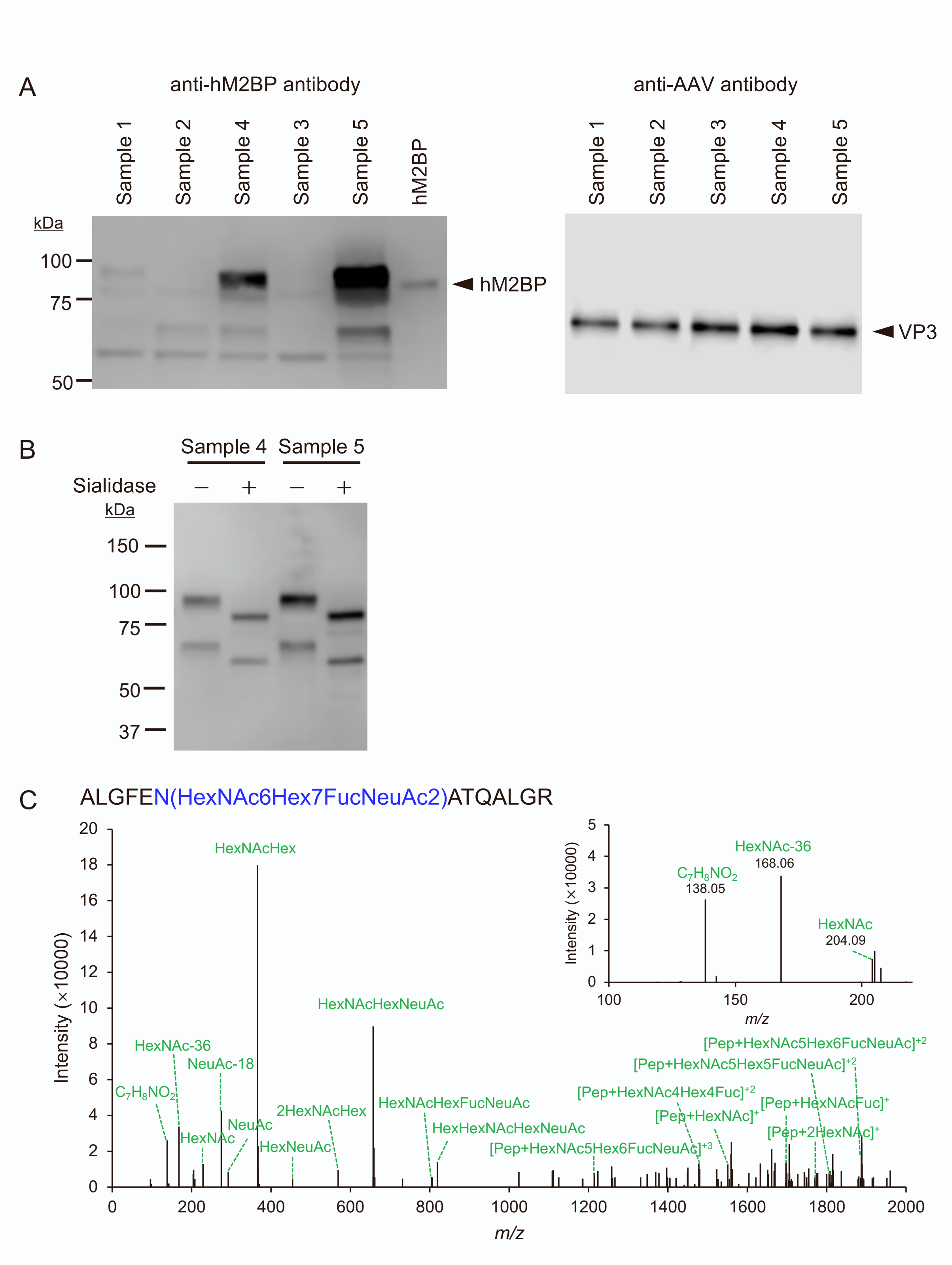

Figure S1. (A) Western blots of Samples 1–5 detected with anti-hM2BP antibody (left) and anti-AAV viral proteins antibody (right). (B) Western blot analysis of Samples 4 and 5 detected with anti-hM2BP antibody with and without sialidase treatment. (C) The CID mass spectra of hM2BP A64–R76 modified with HexNAc6Hex7FucNeuAc2 in Sample 4.

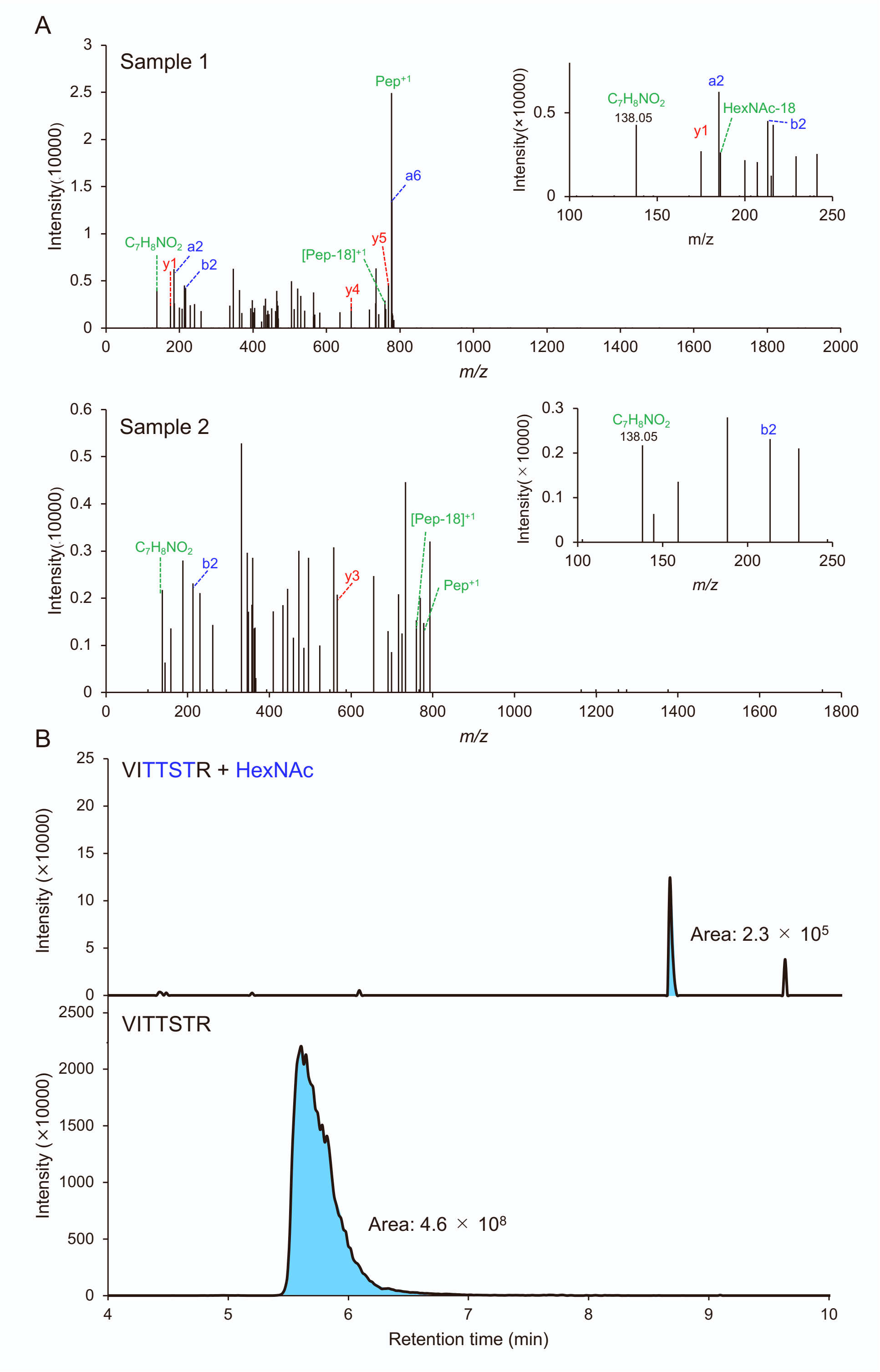

Figure S2. (A) The CID mass spectra of V239–R245 modified with HexNAc for Samples 1 and 2. (B) Extracted ion chromatograms (XICs) of V239–R245 modified with HexNAc (*m/z* 389.2284) (top) and unmodified V239–R245 (*m/z* 490.7697) (bottom). Each MS area was calculated using Byos software. The amount of peptide modified with *O*-GlcNAc (*a*) was calculated with the equation: a = [2.3×10^5 of *O*-GlcNAc modified peptide's MS area] / ([*O*-GlcNAc modified peptide's MS area] + [4.6×10^8 of unmodified peptide's MS area]). The percentage of particle modified with *O*-GlcNAc (*p*) was calculated with the equation: $p = 1^{60} - (1\text{-}0.0005)^{60}$.

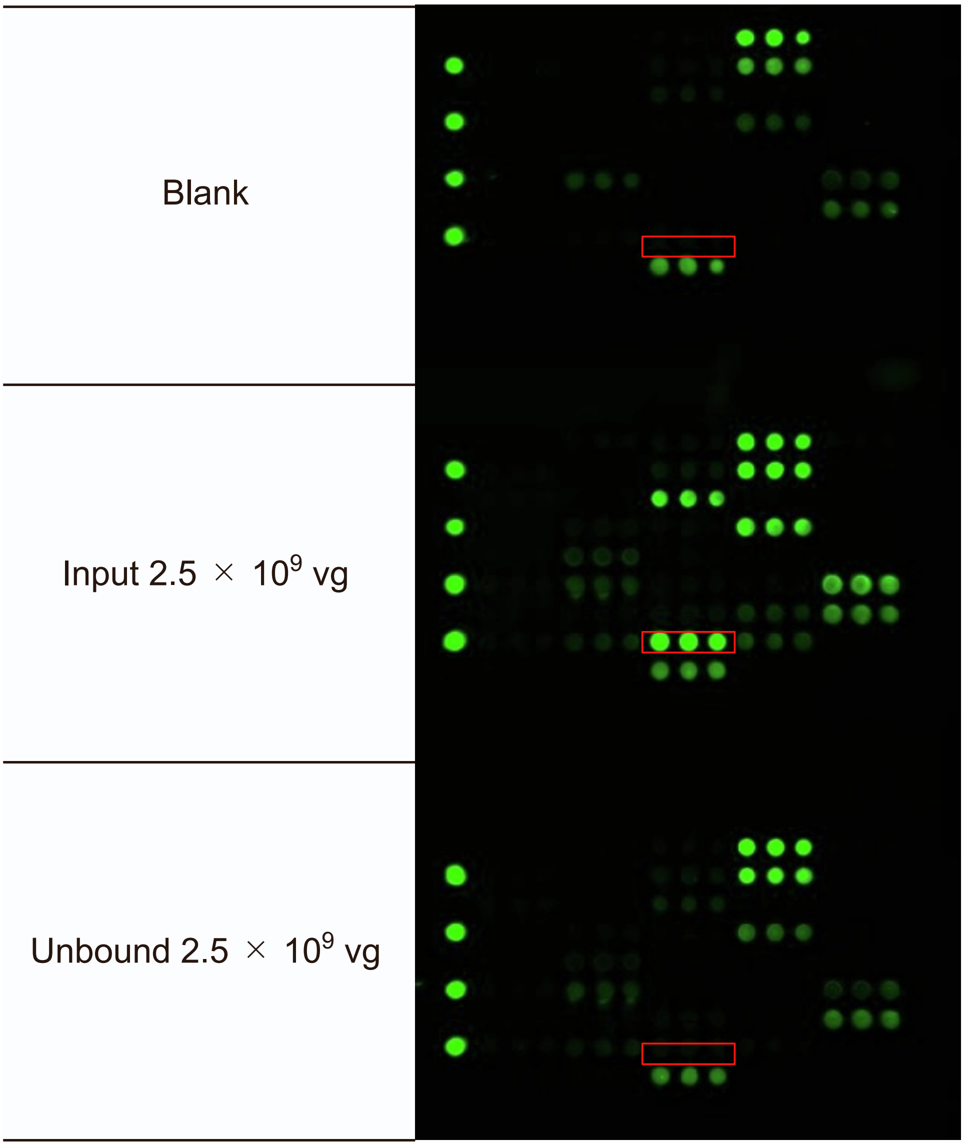

Figure S3. The signals detected by lectin microarray for 2.5×10^9 vg rAAV6 samples as described for western blotting. The area of ABA signals is marked by in a red box. The Blank array shows the high signals of *Lycopersicon esculentum* lectin (LEL), *Solanum tuberosum* lectin (STL), and *Urtica dioica* lectin (UDA) in triplicate spots caused by binding to the detection antibody. The signals of those lectins were therefore excluded from the lectin microarray analysis.

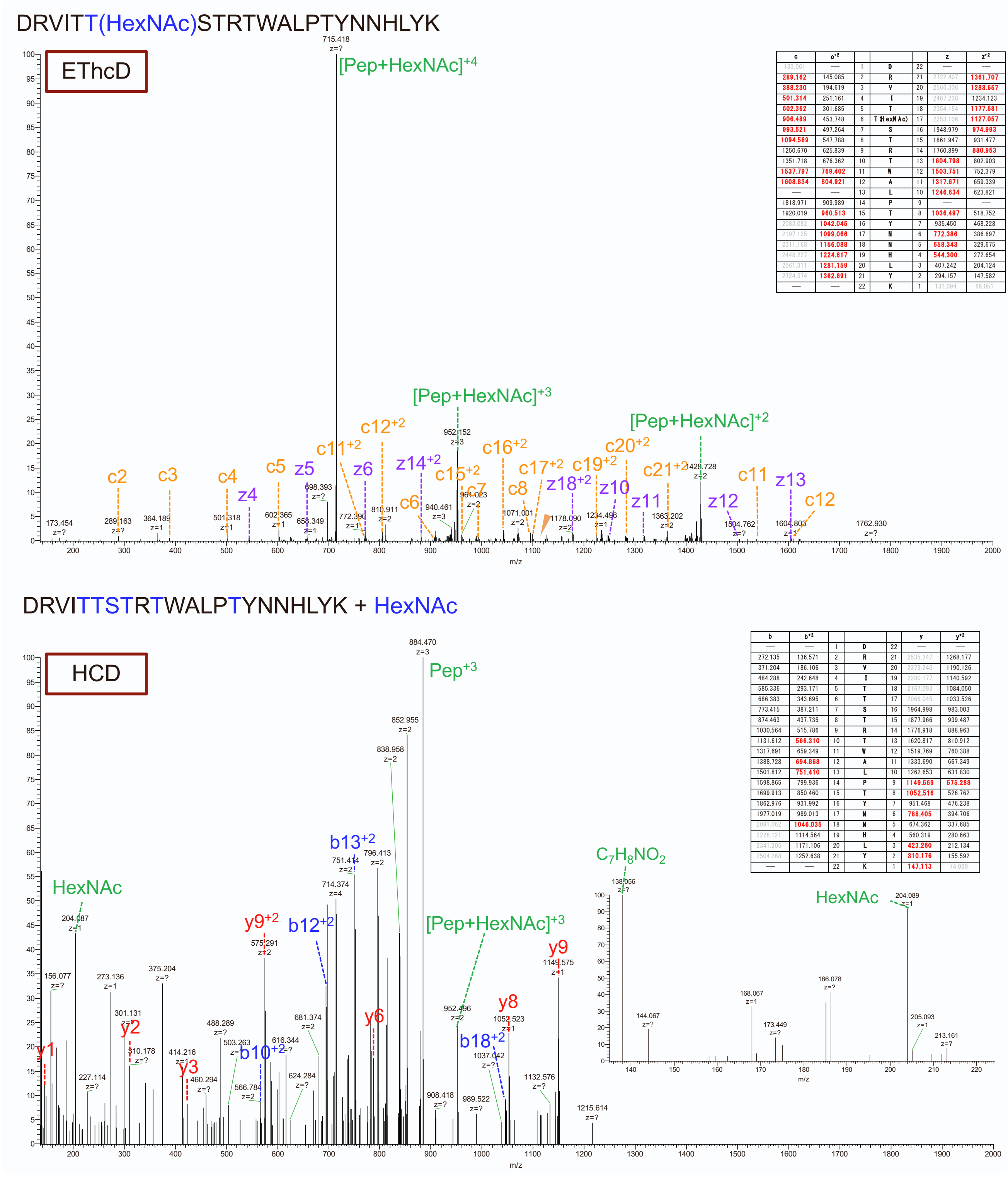

Figure S4. The EThcD and HCD mass spectra of D237–K258 with HexNAc identified in the unbound fraction of rAAV6.

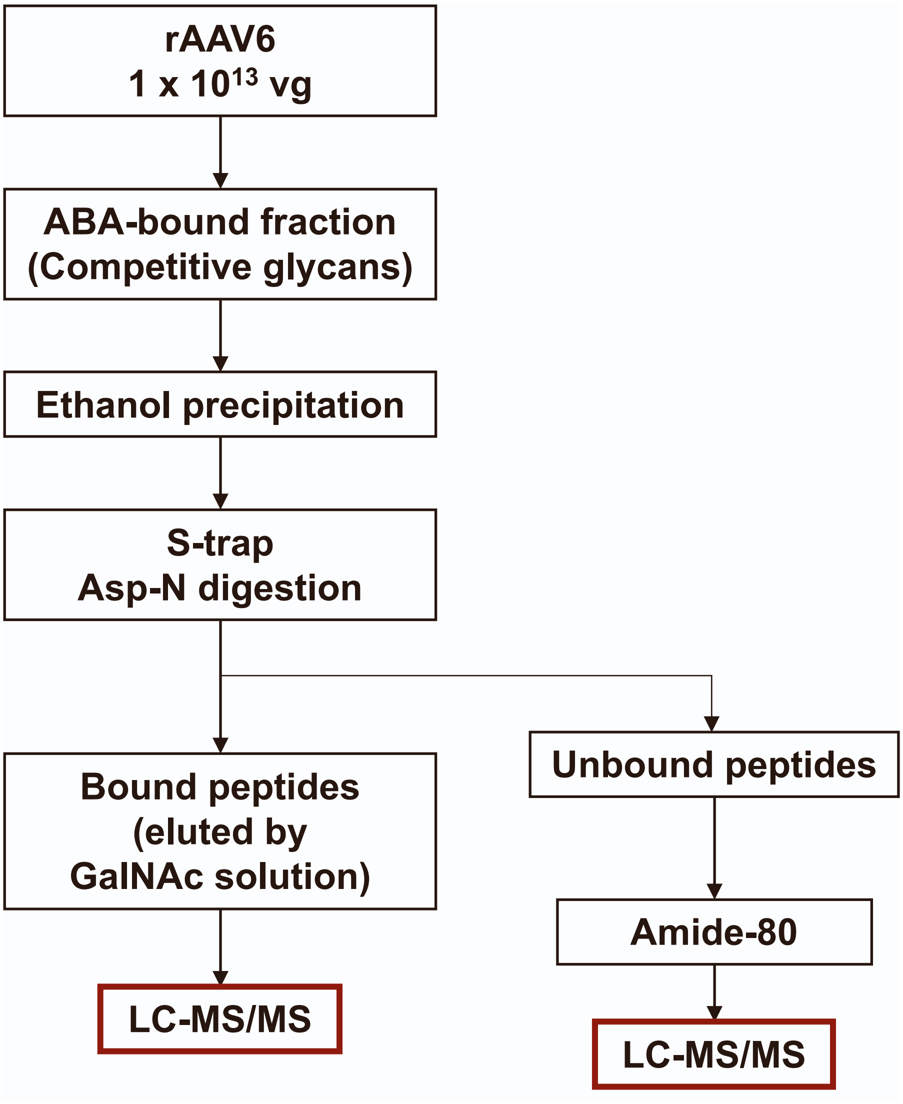

Figure S5. The experimental workflow of LC-MS/MS analysis for the bound fraction of rAAV6 using Sample 1.

A

O-glycan							
DSSSGIGKTGQQPAKKRLNFGQTG (154-177)							
cluster_no:1/member:3							
peak_no	m/z	rt	intensity	Hex	HexNAc	dHex	NeuAc
1	2462.268	40.99	158184080	0	0	0	0
570	2665.354	40.79	184328	0	1	0	0
217	2665.354	39.99	639783				
878	2868.434	39.59	98248	0	2	0	0

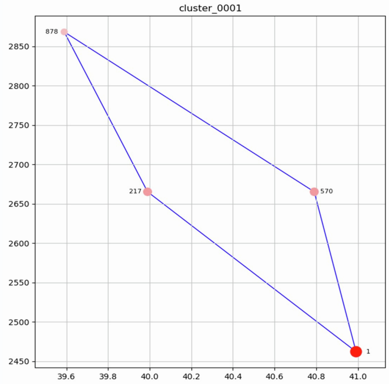

O-glycan							
DPQPLGEPPATPAAVGPTTM (oxi)ASGGGAPM (oxi)A (184-212)							
cluster_no:5/member:10							
peak_no	m/z	rt	intensity	Hex	HexNAc	dHex	NeuAc
497	2881.321	61.9	302018	0	1	0	0
346	3043.372	61.06	572997	1	1	0	0
1985	3043.376	62.69	21129				
1102	3043.378	62.01	66605				
636	3084.399	60.83	191739	0	2	0	0
1970	3246.451	59.43	21498	1	2	0	0
879	3246.452	60.08	101782				
2698	3246.453	58.76	9019				
2361	3334.466	64.29	13970	1	1	0	1
596	3334.469	62.63	215796				
1677	3334.469	63.46	30550				
1041	3334.47	60.26	74799				
2550	3408.509	58.76	11121	2	2	0	0
2117	3537.548	60.35	18564	1	2	0	1
780	3537.549	61.58	123455				
1480	3537.549	59.21	38553				
282	3625.561	61.06	774921	1	1	0	2
1706	3625.563	63.29	29419				
1945	3625.566	64.73	22098				
431	3828.646	59.87	388762	1	2	0	2
1005	3845.674	59.87	81457	2	2	1	1

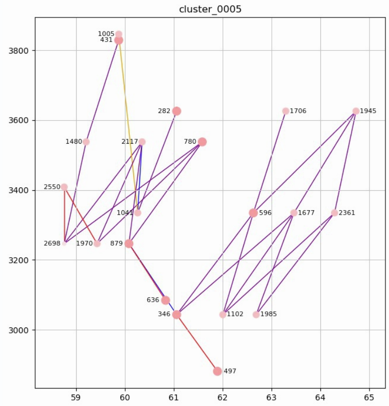

B DSSSGIGKTGQQPAKKRLNFGQTG

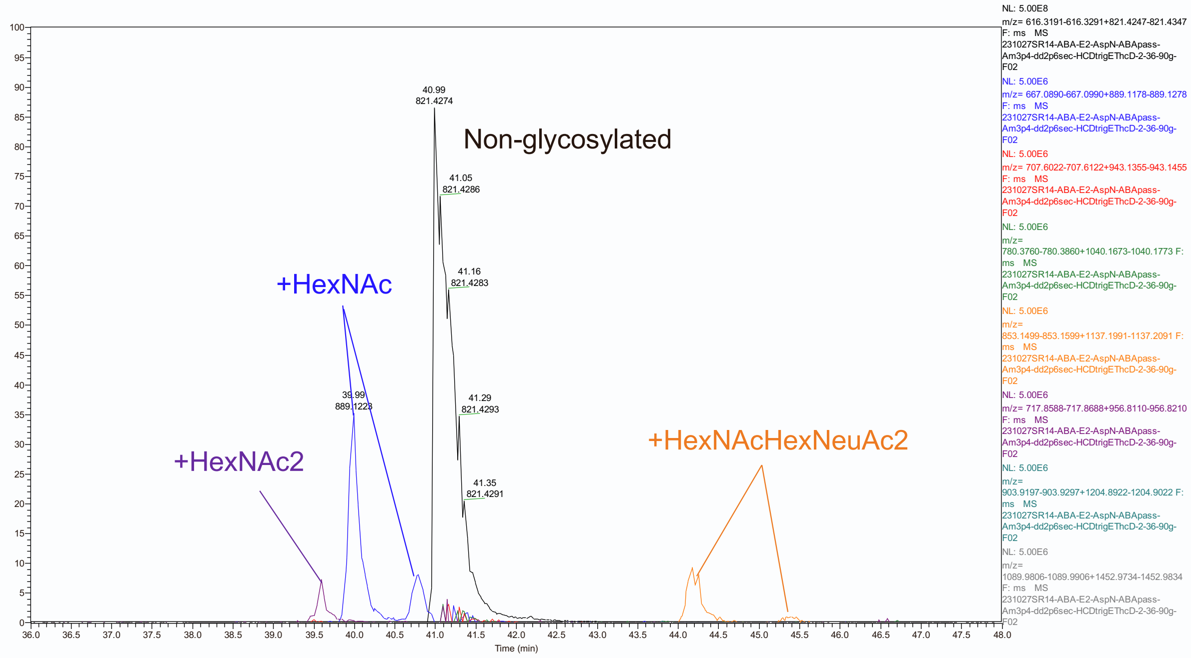

Figure S6. (A) Glycopeptide clusters assigned by GRable for D156–G177 and D184–A212 of the fractionated rAAV6. (B) XICs of D156–G177 modified with mucin-type *O*-glycan clusters. The signal of non-glycosylated peptide (*m/z* 821.4297 ± 0.005) is colored black, HexNAc (*m/z* 889.1228 ± 0.005) is blue, HexNAc2 (*m/z* 956.8160 ± 0.005) is purple and HexNAcHexNeuAc2 (*m/z* 1137.1941 ± 0.005) is orange.

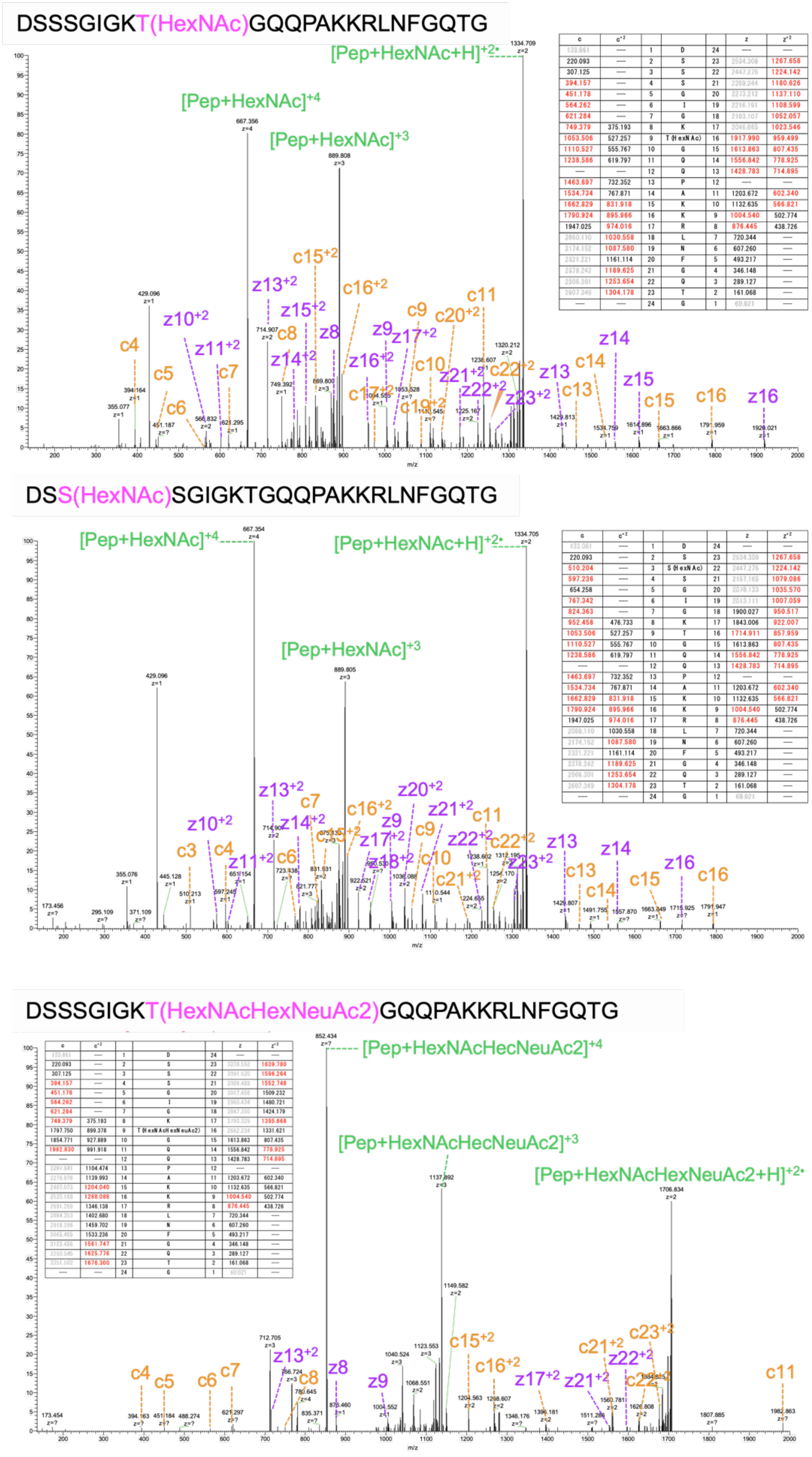

Figure S7. The EThcD mass spectra of D156–G177 modified with HexNAc at T162 (top), HexNAc at S156 (middle) and HexNAcHexNeuAc2 (bottom).

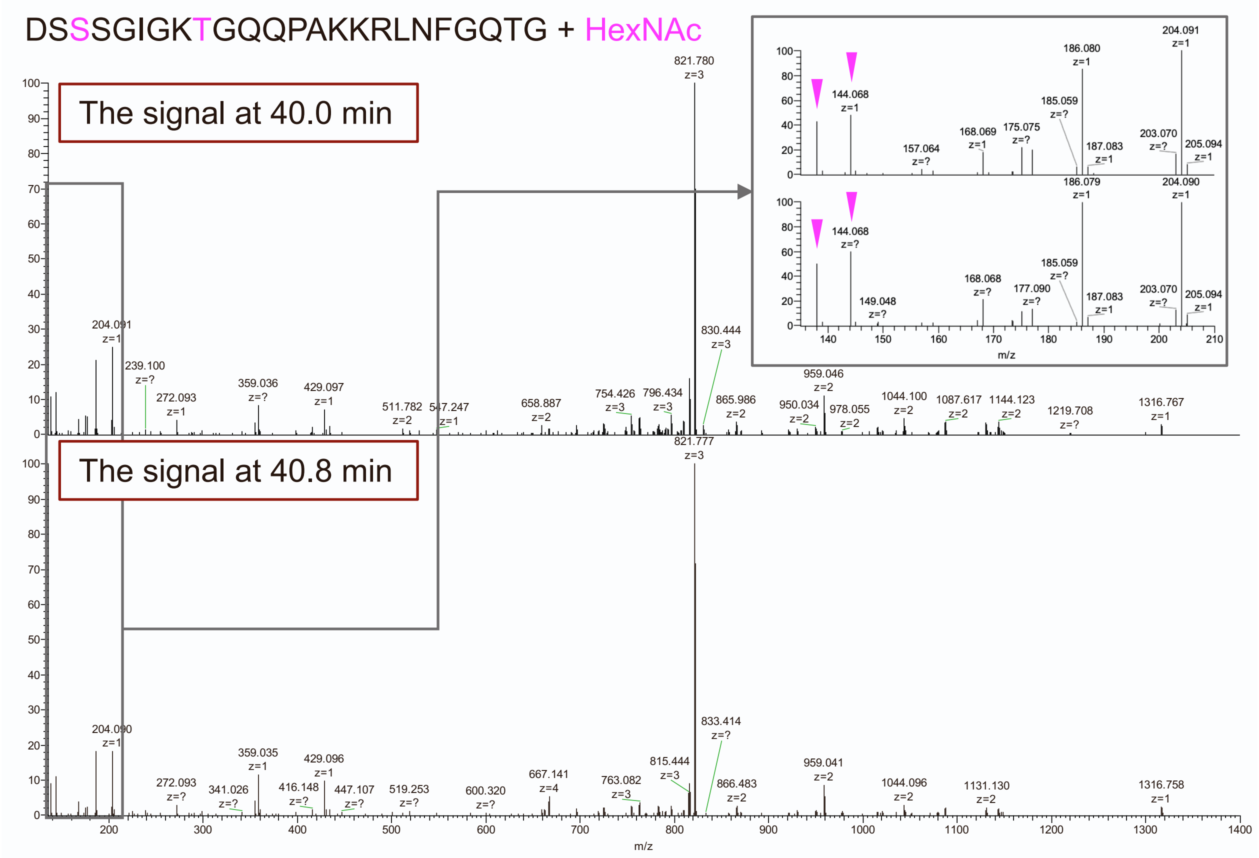

Figure S8. The HCD mass spectra of the signals at 40.0 min and 40.8min for D156–G177 modified with HexNAc. The signals *m/z* 138 and *m/z* 144 showed similar intensities.

DPQPLGEPPAT(HexNAc)PAAVGPT(HexNAc)TM(oxi)ASGGGAPM(oxi)A

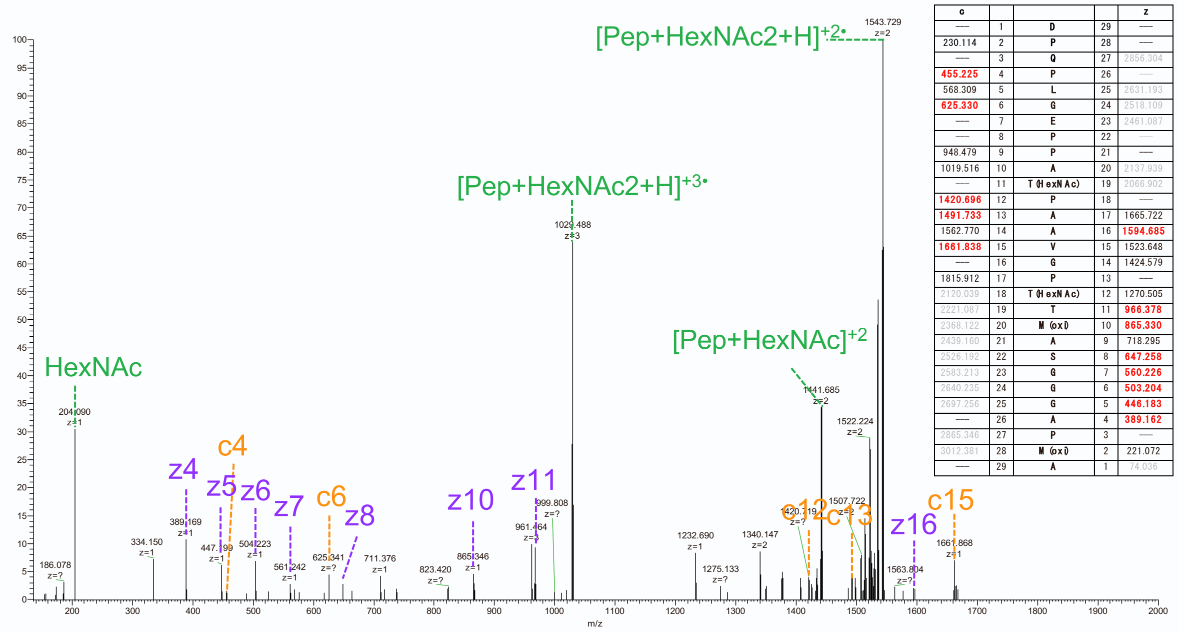

c				z
---	1	D	29	---
230.114	2	P	28	---
---	3	Q	27	2856.304
455.225	4	P	26	---
568.309	5	L	25	2631.193
625.330	6	G	24	2518.109
---	7	E	23	2461.087
---	8	P	22	---
948.479	9	P	21	---
1019.516	10	A	20	2137.939
---	11	T (HexNAc)	19	2066.902
1420.696	12	P	18	---
1491.733	13	A	17	1665.722
1562.770	14	A	16	1594.685
1661.838	15	V	15	1523.648
---	16	G	14	1424.579
1815.912	17	P	13	---
2120.039	18	T (HexNAc)	12	1270.505
2221.087	19	T	11	966.378
2368.122	20	M (oxi)	10	865.330
2439.160	21	A	9	718.295
2526.192	22	S	8	647.258
2583.213	23	G	7	560.226
2640.235	24	G	6	503.204
2697.256	25	G	5	446.183
---	26	A	4	389.162
2865.346	27	P	3	---
3012.381	28	M (oxi)	2	221.072
---	29	A	1	74.036

DPQPLGEPPAT(HexNAcHexNeuAc2)PAAVGPT(HexNAc)TM(oxi)ASGGGAPM(oxi)A

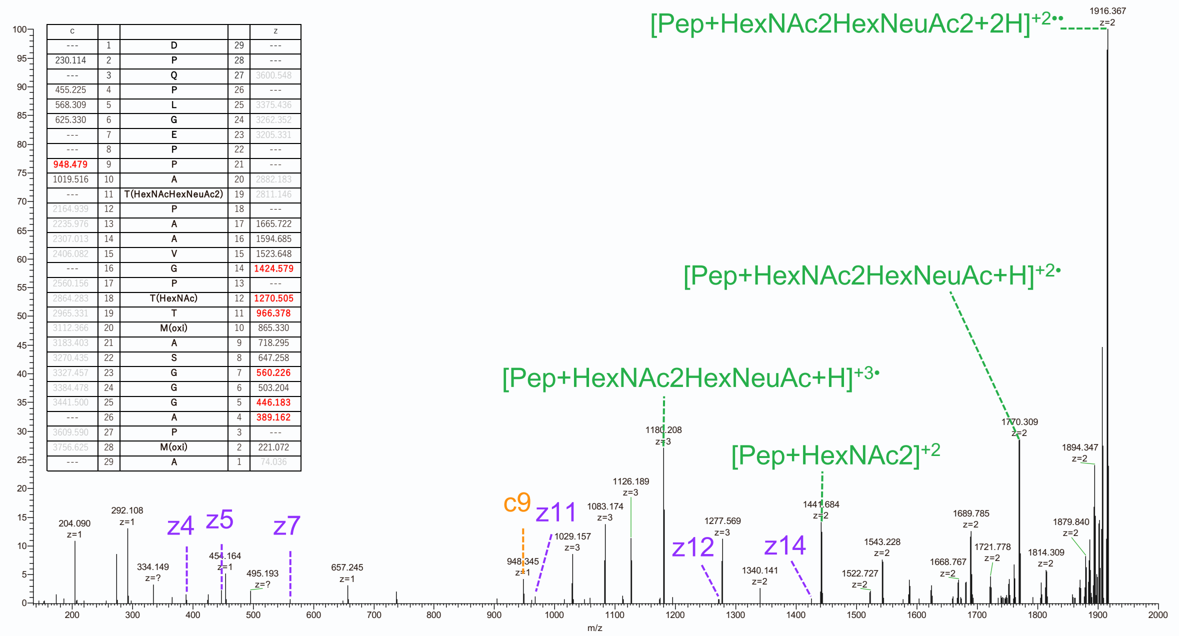

c				z
---	1	D	29	---
230.114	2	P	28	---
---	3	Q	27	3600.548
455.225	4	P	26	---
568.309	5	L	25	3375.436
625.330	6	G	24	3262.352
---	7	E	23	3205.331
---	8	P	22	---
948.479	9	P	21	---
1019.516	10	A	20	2882.183
---	11	T(HexNAcHexNeuAc2)	19	2811.146
2164.939	12	P	18	---
2235.976	13	A	17	1665.722
2307.013	14	A	16	1594.685
2406.082	15	V	15	1523.648
---	16	G	14	1424.579
2560.156	17	P	13	---
2864.283	18	T(HexNAc)	12	1270.505
2965.331	19	T	11	966.378
3112.366	20	M(oxi)	10	865.330
3183.403	21	A	9	718.295
3270.435	22	S	8	647.258
3327.457	23	G	7	560.226
3384.478	24	G	6	503.204
3441.500	25	G	5	446.183
---	26	A	4	389.162
3609.590	27	P	3	---
3756.625	28	M(oxi)	2	221.072
---	29	A	1	74.036

Figure S9. The EThcD mass spectra of D184–A212 modified with HexNAc at T194 and T201 (top), and HexNAcHexNeuAc2 at S194 and HexNAc at T201 (bottom).

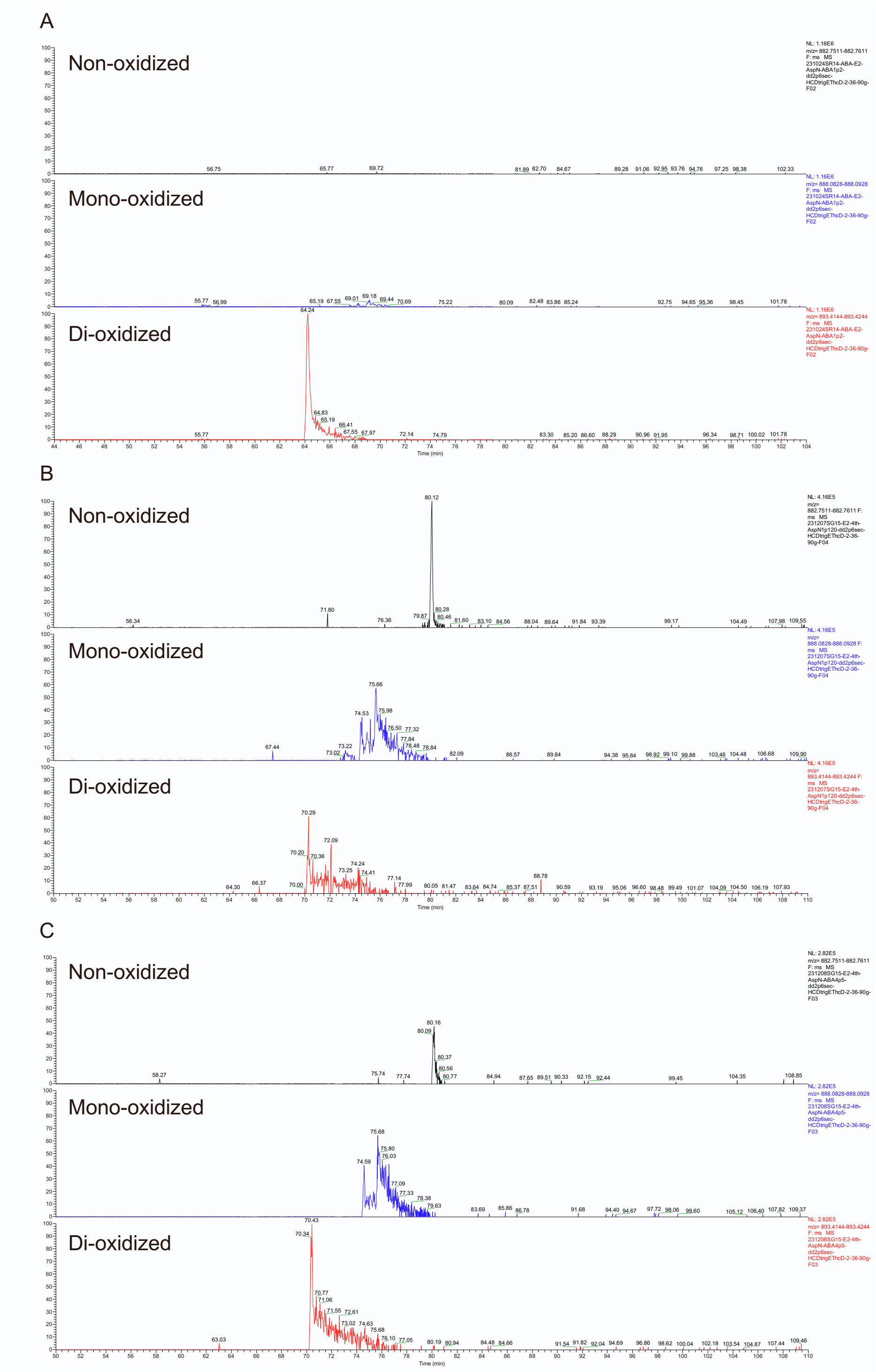

Figure S10. XICs of D184-A212 modified by non- (*m/z* 882.7561 ± 0.005; top), mono- (*m/z* 882.0878 ± 0.005; middle), and di-oxidized form (*m/z* 893.4194 ± 0.005; bottom) in (A) the ABA-bound fraction of the peptides digested from ABA-bound rAAV6 with the S-trap column, (B) the peptides digested from ABA-bound rAAV6 without the S-trap column, and (C) the peptides digested from ABA-bound rAAV6 with the S-trap column. The non-oxidized form was abundant for rAAV6 digested without the S-trap digestion, whereas the ratio of mono- and di-oxidized form to non-oxidized form increased after the S-trap digestion. We therefore suspect that some reagent used in the S-trap digestion may accelerate the oxidation of methionine.

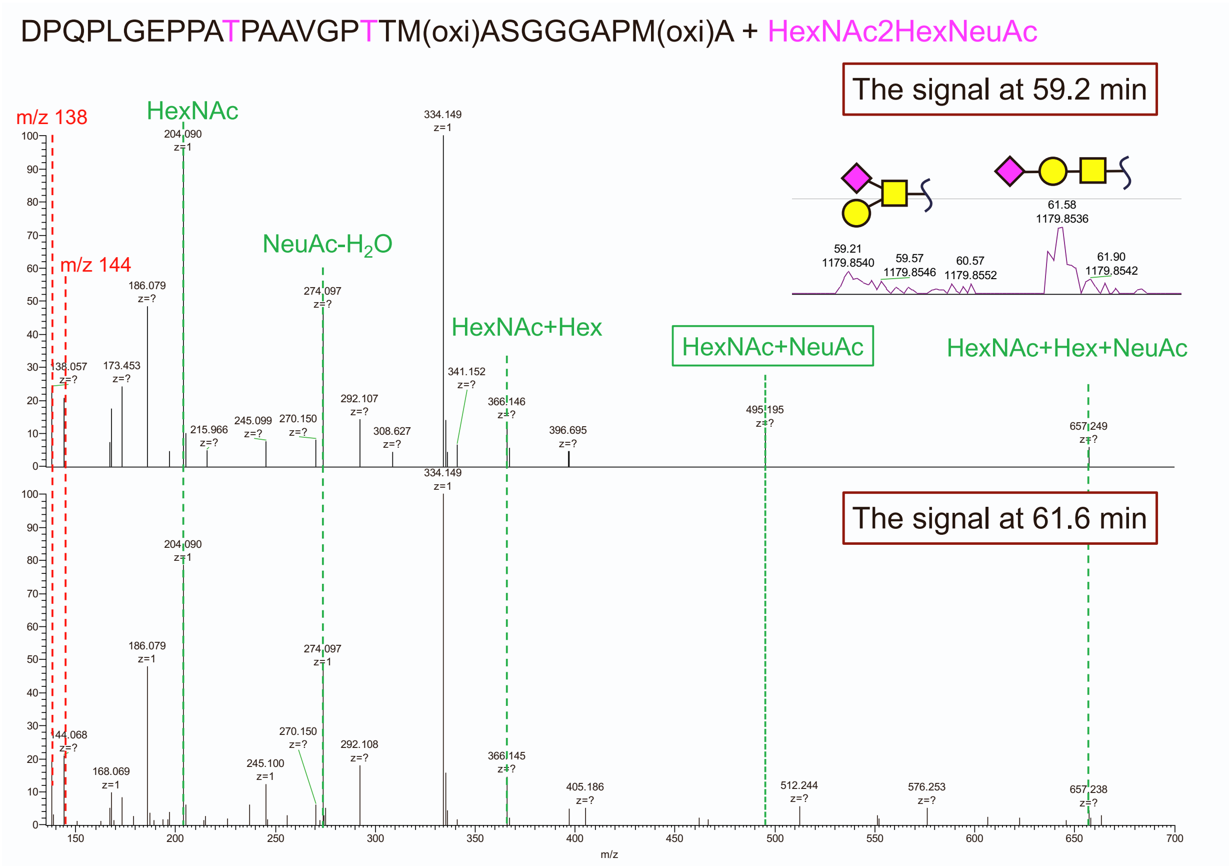

Figure S11. The HCD mass spectra of the signals at 59.2 min and 61.6 min for D184–A212 modified with HexNAc2HexNeuAc. The signals *m/z* 138 and *m/z* 144 showed similar intensities.

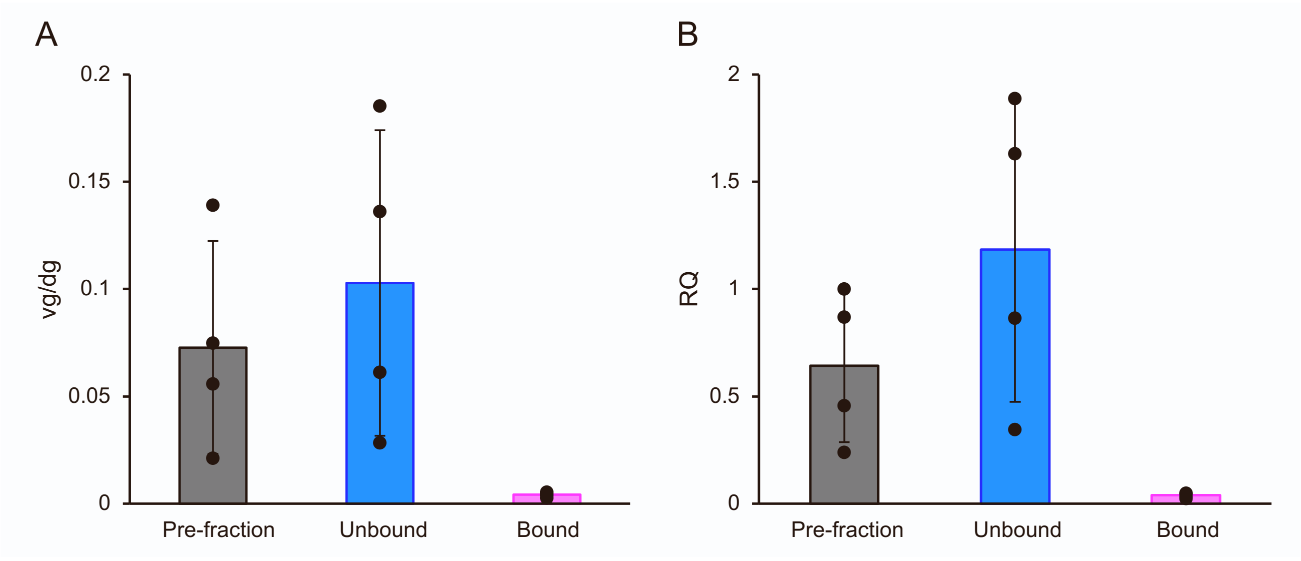

Figure S12. (A) rAAV6 genomes per mouse diploid genome (vg/dg) and (B) *hFIX* mRNA levels in liver tissue were determined using qPCR 8 weeks after rAAV6 administration. Individual points are shown in black circles, and error bars shows the SD value for n = 4.

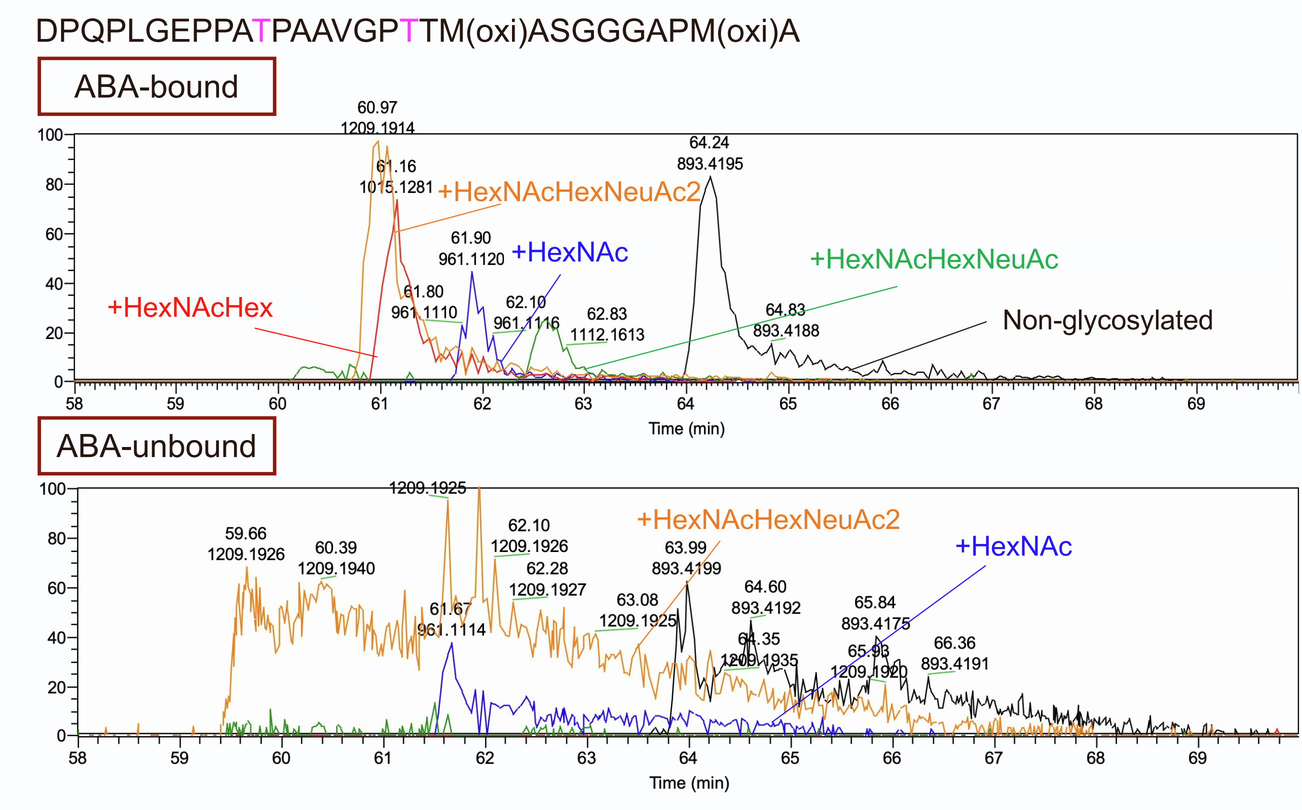

Figure S13. XICs of D184–A212 modified with mucin-type *O*-glycan clusters. The signal of non-glycosylated peptide (*m/z* 893.4194 ± 0.005) is colored black, HexNAc (*m/z* 961.1025 ± 0.005) is blue, HexNAcHex2 (*m/z* 1015.1231 ± 0.005) is red, HexNAcHexNeuAc (*m/z* 1112.1619 ± 0.005) is green, and HexNAcHexNeuAc2 (*m/z* 1209.1937 ± 0.005) is orange.
